# Recent Advances in the Chemistry of Metal Carbamates

**DOI:** 10.3390/molecules25163603

**Published:** 2020-08-07

**Authors:** Giulio Bresciani, Lorenzo Biancalana, Guido Pampaloni, Fabio Marchetti

**Affiliations:** Department of Chemistry and Industrial Chemistry, University of Pisa, Via G. Moruzzi 13, I-56124 Pisa, Italy; giulio.bresciani@dcci.unipi.it (G.B.); lorenzo.biancalana@unipi.it (L.B.)

**Keywords:** carbon dioxide activation, amines, carbamate, metal complexes, catalysis, material chemistry

## Abstract

Following a related review dating back to 2003, the present review discusses in detail the various synthetic, structural and reactivity aspects of metal species containing one or more carbamato ligands, representing a large family of compounds across all the periodic table. A preliminary overview is provided on the reactivity of carbon dioxide with amines, and emphasis is given to recent findings concerning applications in various fields.


**Index:**


**1. Introduction**
2
**2. The Reactivity of Carbon Dioxide with Amines and other *N*-Donors**
2 2.1. CO_2_/Amine Equilibria in Aqueous Solution3 2.2. Amine/CO_2_ Interaction: Isolation and Characterization of Carbamato Salts5 2.3. Stabilization of Carbamates by Superbases6
**3. Synthesis, Structure and Reactivity of Metal Carbamates**
10 3.1. Homoleptic Carbamato Complexes11 3.2. Heteroleptic Carbamato Complexes15 3.3. Dynamics and Reactivity of Metal Carbamato Complexes22 3.4. Crystallographic and Spectroscopic Features of Carbamato Ligands26
**4. Catalysis with Metal Carbamates**
29 4.1. CO_2_ Activation Routes29 4.2. Other Catalytic Processes32
**5. Other Applications**
33
**6. Conclusions**
38
**References**
39

## 1. Introduction

Carbon dioxide is a an easily available and non-toxic chemical, and at the same time, it is implicated in environmental, energy and sustainability issues [1,2,3,4]. Thus, the last two decades have witnessed an unprecedentedly intense effort of academic and industrial research in two main directions, i.e., to exploit CO_2_ as a C_1_ synthon for organic synthesis [5,6,7,8,9,10,11] and to develop more and more efficient systems able to capture and store CO_2_ [12,13]. The former goal is challenging for a variety of reactions, and a wide number of metal catalysts have been proposed to access valuable organic compounds and materials via CO_2_ fixation strategies, which, however, require harsh conditions (high temperature and CO_2_ pressure) in several cases [14,15]. It is worthy to note that, differently to other small molecules such as its relative carbon monoxide, carbon dioxide is a “bad” ligand for transition metals; therefore, examples of simple coordination compounds are relatively rare [16,17,18,19,20,21] and, accordingly, metal catalysts working in CO_2_ activation routes usually exert their action without the intermediacy of metal-CO_2_ adducts. The “weak point” in the apparently unscratchable robustness of carbon dioxide is the susceptibility to nucleophilic attacks at the carbon atom [22,23,24]. Thus, a range of nucleophilic reagents, including neutral *N*-heterocyclic carbenes [25,26], are known to react with CO_2_ even under mild conditions, and some chemistry at transition metal centers is provided by the possibility of CO_2_ insertion into the bond between a metal atom and a suitable anionic ligand, e.g., alkyl, allyl, alkoxide and hydride [27,28,29,30,31,32]. In this context, amines are key reactants towards carbon dioxide, and indeed carbon dioxide/amine systems have been intensively investigated in the field of capture/storage [33,34,35] and exploited for the incorporation of the CO_2_ moiety within organic structures [36,37,38,39]. Furthermore, CO_2_ is also prone to insertion reactions into a variety of metal-amide bonds, generating a carbamato ligand; however, metal complexes containing carbamato ligands are easily available through diverse synthetic routes not requiring the use of pressurized CO_2_. It is remarkable that the preliminary formation of a magnesium-carbamato adduct generated from a lysine residue is widely exploited by the universal Rubisco enzyme to incorporate CO_2_ in biomolecules [40,41]. Notably, metal carbamates (either homoleptic or not) have been reported for a wide number of elements throughout the periodic table and, due to this systematicity and their intriguing properties that will be described below, the chemistry of metal carbamates has seen a significant advance in the recent times. This review follows up a previous review on the same topic published in 2003 by Calderazzo, Pampaloni and coworkers [42]; herein, we will summarize fundamental concepts regarding the reactivity of CO_2_ with amines, then we will discuss synthetic, structural and reactivity aspects of metal carbamates and their potential in various applications, with a particular focus on the findings appeared in the literature after 2003.

## 2. The Reactivity of Carbon Dioxide with Amines and other *N*-Donors

**Scheme 1:**
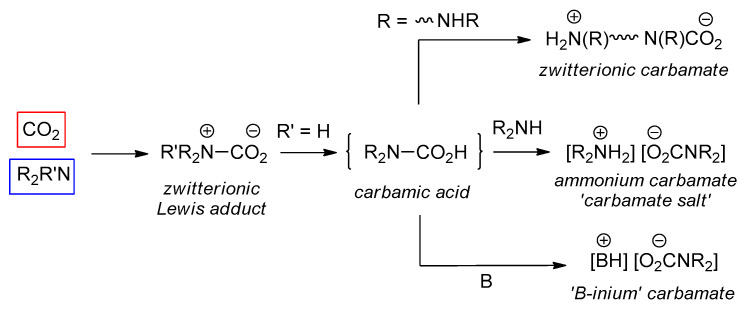
Overall reactivity of CO_2_/amine systems.

An overview of the reactivity of CO_2_ with amines is depicted in Scheme 1. When the lone pair on the nitrogen atom attacks the carbon atom of CO_2_, a zwitterionic Lewis acid/base adduct can be formed. At this step, a hydrogen atom can migrate from nitrogen to oxygen, affording the elusive carbamic acid. The detection of this species is extremely difficult [43,44,45,46] and its isolation rare [47]. The fate of the carbamic acid depends on the nature of the amine and the reaction environment. Most frequently, a second equivalent of *N*-donor can act as a Brønsted base affording an ammonium carbamate salt. In some cases, deprotonation of a carbamic acid may be operated by a second basic group present in the structure of the employed amine (e.g., diamine), affording a zwitterionic carbamate. Furthermore, the deprotonation can be forced when an external base, B, is added to the system, producing a “B-inium” carbamate.

In principle, ammonia and primary amines hold the potential to undergo respectively three and two carbonations per molecule; this kind of reactivity was proposed for ethylamine and cyclohexylamine in acetonitrile in the presence of penta-alkylguananidines, based on a ^15^N-NMR study [48], but no other data have been reported as of today.

In agreement with the aim of this review, this section intends to summarize concepts reported in the previous review [42] and to provide information about recent developments in the study of CO_2_/amine systems. The first part describes the carbonation of amines in aqueous solution and the most recent progress in this field achieved by using natural α-amino acids (Section 2.1). Next, the isolation and characterization of pure carbamate salts will be discussed (Section 2.2). Finally, recent advances in the stabilization of CO_2_ adducts by using amidine/guanidine ‘superbases’ [49] and the CO_2_/amine/superbase system is presented (Section 2.3).

### 2.1. CO_2_/Amine Equilibria in Aqueous Solution

**Scheme 2:**
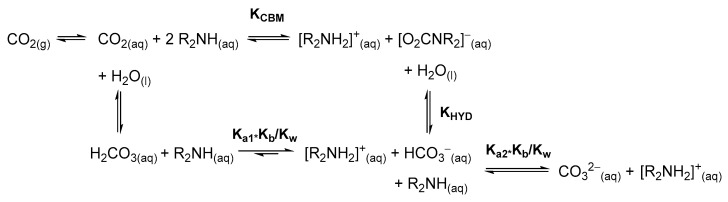
Equilibria involving carbon dioxide and amines in aqueous solution.

The CO_2_/amine system is finely governed by parameters such as the nature of the solvent, temperature and solution pH. Thus, the concentration of carbamate in solution strictly depends on the experimental conditions. Many reactions involving amines and CO_2_ have been performed in water, where the competition between the formation of the carbamato species and that of carbonate/bicarbonate anions must be taken into account (Scheme 2). Despite this complication, kinetic and thermodynamic aspects have been widely investigated [35,50,51], also in view of the search for optimal substrates and conditions for industrial CO_2_ capture.

A collection of thermodynamic parameters concerning the carbonation of amines in water is compiled in Table 1, including data from the most recent studies [42,52,53,54,55,56,57,58] and relevant to amino acids [59,60,61,62,63]. Additional data, including kinetic constants, are provided in Table S1 (Supplementary Information).

In most of the cases, either using ammonia, primary or secondary amines, the equilibrium constants regulating the hydrolysis of carbamate to bicarbonate (**K_HYD_** in Scheme 2) are lower than 1, while equilibrium constants for the formation of carbamate (**K_CBM_** in Scheme 2) are >10^3^. The only exception to this trend (**K_HYD_** >> **K_CBM_**) was observed for aniline [64], while the formation of carbamato salts derived from diisopropylamine [65], di-*sec*-butylamine [66] and 2-amino-2-methyl-1-propanol (AMP) [52] was not assessed. Piperazine may form a bis-carbamate, but the generation of the second carbamato unit is unfavorable (**K_HYD_** > **K_CBM_**) [53].

Overall, **K_CBM_** and **K_HYD_** values outline the absence of a clear correlation with the Brønsted basicity (pK_b_) of the amine function [42]. Among other factors, the Lewis basicity of the corresponding amide, R_2_N^−^, appears to play a significant role in the stabilization of the ammonium carbamate.

In the last decades, the capture of CO_2_ has attracted the interest of the scientific community, and the possibility of storing and releasing carbon dioxide using non-toxic or even natural products represents an intriguing prospect [12,67,68,69,70]. In this context, the use of amino acids as adsorbers for PCC (post-combustion capture) of CO_2_ has been intensively investigated [51,71]; nevertheless, thermodynamic and kinetic studies on the carbonation of amino acids did not receive adequate attention. Equilibrium constants for the formation (K_CBM_) and hydrolysis (K_HYD_) of amino acid carbamates were extrapolated from experimental data collected on sodium or potassium aminocarboxylates in aqueous solution (Table 1). In particular, Jensen and Faurholt [61] reported that β-alanine reacts with CO_2_ approximately 1.5 times faster than α-alanine, and its carbamate is more stable toward hydrolysis. The formation and hydrolysis constants of lysine carbamate were recently calculated [63]. The presence of a second amino group on the side chain of lysine allows the formation of two carbamato moieties, but the second carbonation occurs under high CO_2_ loading, making the hydrolysis predominant.

### 2.2. Amine/CO_2_ Interaction: Isolation and Characterization of Carbamato Salts

The previous considerations are valid for aqueous solutions, and are not extensible to other solvents, including the use of the amine itself as solvent. In fact, bulky amines are not reactive to CO_2_ in water, whereas the same amines may be able to generate the corresponding carbamate under anhydrous, non-competitive conditions [47]. The uptake of CO_2_, measured under anhydrous conditions and at atmospheric pressure in neat amines NHR_2_ (R = Bu, ^i^Pr, Cy), corresponds to a CO_2_/amine molar ratio of approximately 0.5, as expected for the formation of the ammonium carbamate [NH_2_R_2_][O_2_CNR_2_]. Under such conditions, pure alkylammonium alkylcarbamates of several primary and secondary amines were isolated as colorless solids [42].

Since 2003, many other alkylammonium alkylcarbamates of cyclic amines [74,75,76,77], substituted amines [78,79,80,81,82] and diamines [83,84,85] have been isolated and characterized by IR and NMR spectroscopy, and by X-Ray diffraction in a number of cases. Noteworthy, some of these carbamates were obtained upon air exposure, revealing the capability of the respective amines of trapping CO_2_ from the environment [74,75,76,81,82,83,84]. All the compounds cited above show intense bands due to C=O vibrations in the IR spectral region between 1650 and 1400 cm^−1^.

**Scheme 3:**
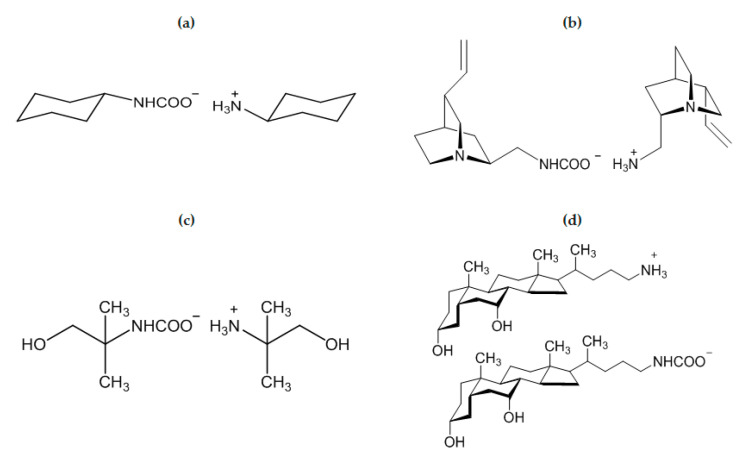
Structures of alkylammonium alkylcarbamate derived from cyclohexylamine (**a**) [75], 2-amino-2-methyl-1-propanol, AMP (**b**) [82], quincoridine (**c**) [80], C24 amine derivative of chenodeoxycholic acid (**d**) [81].

In the course of a study on the crystallization of amines assisted by 1,5-dichloro-trans-9,10-diethynyl-9,10-dihydroanthracene-9,10-diol (DDDA), Mondal and Bhunia [75] found that cyclohexylamine (Scheme 3a), cycloheptylamine and piperidine undergo aerial carbonation affording the corresponding ammonium carbamate. Interestingly, no carbonation was observed for cyclopentylamine, even in the presence of DDDA. This result was attributed to the envelope conformation of cyclopentylamine, which is more rigid respect to the chair conformations of other cycloamines.

The first examples of chiral ammonium carbamates derived from chiral primary amines were described by Neda et al. [80], as obtained by the treatment of amino derivatives of quincorine and quincoridine with carbon dioxide in diethyl ether (Scheme 3b). The isolated colorless solids are stable for several days in solution at ambient temperature and thermally stable until 120 °C in a solid state. Above this temperature, the compounds lose carbon dioxide re-converting into amines, and the overall procedure represents a convenient purification method of quincorine and quincoridine. As previously discussed, AMP carbamate (AMP = 2-amino-2-methyl-1-propanol) has a very low formation constant in water and its hydrolysis is rapid, thus, only traces of this compound were detected by NMR in aqueous solution (see Table 1 and related discussion). On the other hand, when neat AMP was exposed to air for five days, AMP carbamate was recovered as a white solid (Scheme 3c). It survives in air for a limited time (max. 10 days) [82]. The crystal structure reveals asymmetric units composed of AMPH^+^ and AMP carbamate, both involved in intermolecular hydrogen bonds. Notably, aerial CO_2_ capture was also observed for “amino bile acids” [81], probably enhanced by the presence of OH groups in the structure (Scheme 3d). In general, synthesis and crystallization of carbamates are usually facilitated for precursors containing hydrogen bonding groups [74,75,76,79,82].

**Scheme 4:**
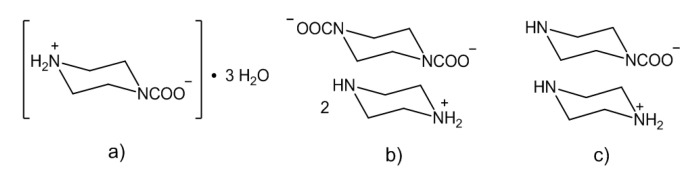
Carbonation products of piperazine: zwitterionic carbamate (**a**), di-carbamate (**b**) and “ammonium” carbamate (**c**).

In theory, a diamine/CO_2_ system should lead to either a zwitterionic carbamate, a diammonium dicarbamate salt or a mixture of both species (Scheme 1) [86,87]. In addition to the two polymorph structures of the zwitterionic ethylenediamine carbamate [^+^NH_3_(CH_2_)_2_NHCOO^−^] [86], only few crystallographic data are available for this class of compounds [79,83,84,85], in particular when compared to the number of structurally characterized ammonium carbamates (see [42] and references above). The diamine/CO_2_ system described above has been recently investigated [85], and both the zwitterionic carbamate (Scheme 4a) and the ammonium dicarbamato species were isolated as crystalline materials from water and a 1:1 water/ethanol mixture (Scheme 4b); the “classical” ammonium carbamate was also obtained (Scheme 4c). In summary, the stability of ammonium carbamates benefits from the presence of sterically hindered or non-flexible substituents on the amine, and this feature may find application for the capture of carbon dioxide from the environment.

### 2.3. Stabilization of Carbamates by Superbases

**Scheme 5:**
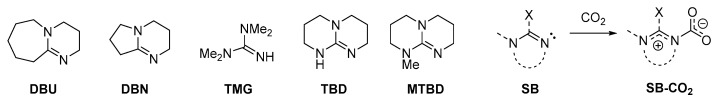
Structures of commonly employed amidines/guanidines “superbases” (SB) and reaction with CO_2_ affording a zwitterionic carbamate. **DBU** = 1,5-diazabiciclo(5.4.0)undec-7-ene; **DBN** = 1,5-diazabicyclo[4.3.0]non-5-ene; **TMG** = 1,1,3,3-tetramethylguanidine; **TBD** = 1,5,7-triazabicyclo[4.4.0]dec-5-ene; **MTBD** = 7-methyl-1,5,7-triazabicyclo(4.4.0)dec-5-ene; **SB** = superbase (general structure).

The interaction of a tertiary alkylamine or pyridine with CO_2_ is expected to lead to a zwitterionic Lewis acid-base adduct (Scheme **1**). However, this type of compounds has never been experimentally observed, even for *N*-donors bearing a significant nucleophilicity (e.g., DABCO, quinuclidine, 4-dimethylaminopyridine) [88]. In the light of the mechanistic implications for CO_2_ activation, considerable efforts have been directed to the exploration of the topic in the last 15 years. These studies have outlined that zwitterionic CO_2_ adducts may increase their stability when the *N*-donor is an amidine or guanidine (Scheme 5). Indeed, amidines and guanidines, the most famous representatives of each category being 1,5-diazabiciclo(5.4.0)undec-7-ene (DBU) and 1,1,3,3-tetramethylguanidine (TMG), are labeled as “superbases” (SB), in that they possess higher Brønsted basicity respect to common alkylamines [49]. Delocalization of the positive charge within the NC(N) system may compensate unfavorable energetics for charge separation in the zwitterion [24].

**Scheme 6:**
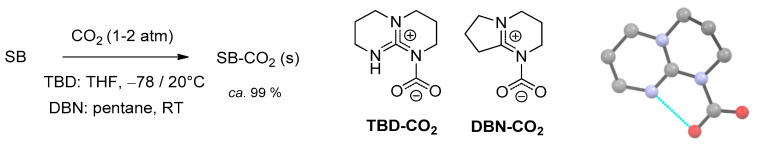
Synthesis of TBD-CO_2_ and DBN-CO_2_, and view of the X-ray structure of TBD-CO_2_ (H atoms omitted, hydrogen bond evidenced as cyano line) [91,92].

The first experimental evidences of SB-CO_2_ adducts were collected analyzing the solid materials obtained from the reactions of 1,5,7-triazabicyclo[4.4.0]dec-5-ene (TBD), 1,5-diazabicyclo[4.3.0]non-5-ene (DBN) and related systems with carbon dioxide in acetonitrile [89,90]. A ^13^C-NMR signal occurring at circa 150 ppm was attributed to the NCO_2_ moiety, whereas a signal around 160 ppm is related to the co-presence of bicarbonate ions. Unambiguous identification of the zwitterionic carbamates TBD-CO_2_ and DBN-CO_2_ (Scheme 6) was later provided by single crystal X-ray diffraction [91,92].

The TBD-CO_2_ adduct is stable in the solid state up to 70 °C under CO_2_ atmosphere and for over 1 month at ambient temperature under Ar. This remarkable stability is ascribable to a hydrogen bonding interaction in the solid state, involving a carbamato oxygen and the neighboring N-H unit. Accordingly, DBN-CO_2_ is less stable and must be conserved under CO_2_ atmosphere.

**Scheme 7:**
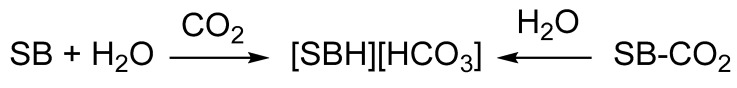
Formation of bicarbonates by reaction of CO_2_ with wet superbases (SB) or by hydrolysis of pre-formed SB-CO_2_ adducts.

However, both TBD-CO_2_ and DBN-CO_2_ have to be prepared and subsequently manipulated under rigorously anhydrous conditions, being extremely sensitive to moisture. As a matter of fact, several attempts to isolate CO_2_ adducts of amidines or guanidines were hampered by the presence of adventitious water in the reaction systems, leading to the crystallization of the corresponding bicarbonates (Scheme 7) [89,91,93,94,95]. This reactivity, common to ordinary alkylamines, is probably enhanced by the higher Brønsted basicity of the “superbases”.

Some amidines and guanidines do not form zwitterionic carbamates, despite being effective in activating CO_2_, most notably DBU [92,96,97]. A DBU-CO_2_ adduct has been frequently proposed as an intermediate in CO_2_-transfer reactions [93,98,99,100], but DBU does not form a carbamate even under a CO_2_ pressure of 57 bar in anhydrous conditions [96]. Instead, DBU immediately reacts with traces of moisture under CO_2_ atmosphere to afford the bicarbonate [DBUH][HCO_3_].

**Scheme 8:**
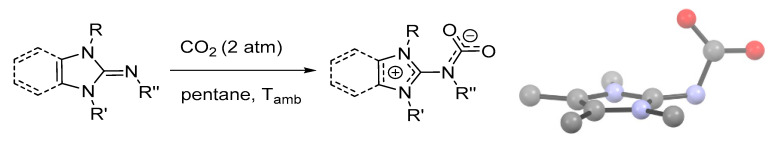
Carbonation of 1,3-dihydro-2*H*-imidazol-2-imines and benzo[*d*]imidazole analogues, and view of the crystal structure of the CO_2_ adduct of 1,3-di-tert-butyl-*N*-methyl-1,3-dihydro-*2H*-imidazol-2-imine (R = R′ = *^t^*Bu; R″ = Me) as a representative example. Drawing based on published structural data, H atoms omitted for clarity [92].

Electronic and steric effects may play a crucial role in the stabilization of zwitterionic carbamates, as recently demonstrated by the preparation and X-ray characterization of a series of CO_2_ adducts of *N*-heterocyclic imines (Scheme 8) [92]. Some of these derivatives display remarkable thermal stability (up to 70 °C under Ar) and resistance towards hydrolysis. In these compounds, the carbamato group is perpendicular to the *N*-heterocyclic ring, at variance to other SB-CO_2_ adducts (e.g., TBD-CO_2_, DBN-CO_2_) that are planar molecules. This conformation promotes π-delocalization in the NCO_2_ fragment, as indicated by the shortening of the N-C bond, while the positive charge is stabilized by the aromaticity of the five-membered imidazolium ring. A collection of bond angles and distances is given in Table S2.

**Scheme 9:**
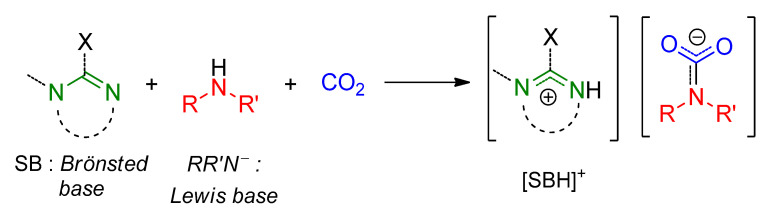
Formation of amidinium/guanidinium carbamates in the amine/superbase/CO_2_ system.

Amidines and guanidines are capable of activating CO_2_ not only by direct interaction (i.e., formation of zwitterionic adducts) but also indirectly, e.g., in combination with alkylamines. In such reactions, the superbase behaves as a Brønsted base and the amide, generated upon deprotonation of the amine, acts as a Lewis base, resulting in the formation of amidinium/guanidinium carbamates (Scheme 9). Amidinium/guanidinium carbamates [SBH][R_2_NCO_2_] are considerably more stable with respect to related alkylammonium alkylcarbamates [R_2_NH_2_][R_2_NCO_2_], by virtue of the higher *p*K_a_ (lower acidity) of the associated cation (amidinium/guanidinium vs. alkylammonium) [101].

**Scheme 10:**
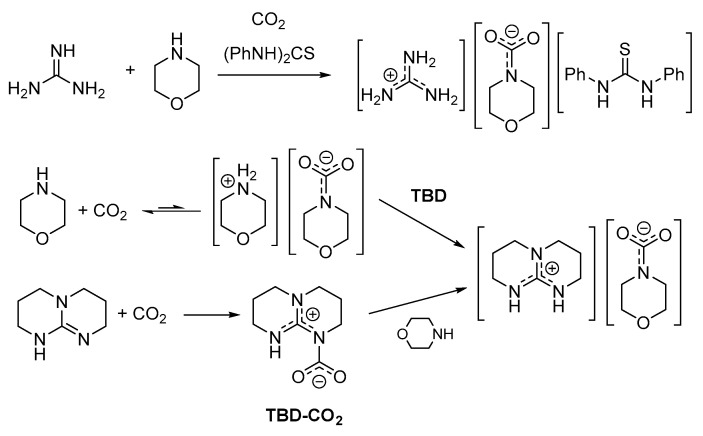
Three different syntheses of a guanidinium carbamate of morpholine.

Spectroscopic evidences (^15^N-NMR) that DBU, TMG and related systems favor the formation of carbamates of primary and secondary amines in organic solvents were presented almost 30 years ago [48,102,103]. The in situ formed carbamates were used for the synthesis of carbamato esters. However, more recently, such concept gained increasing attention for its broader implications. In a series of papers between 2005–2008, it was reported that equimolar mixtures of amidines and NH/OH donors, such as alcohols [104], primary alkylamines [105], α-aminoalcohols [106] or α-aminoesters [107], rapidly react with CO_2_ at ambient pressure quantitatively yielding the respective amidinium alkylcarbonate or carbamate. Most of these amidinium salts are liquids at ambient temperature (or low-melting solids) and their formation can be reversed by heating or by bubbling an inert gas through the liquid phase. Hence, these systems have been classified as “switchable ionic liquids,” with CO_2_ as the element of reversibility. Later, the substrate scope has been extended to the use of guanidines (TMG, TBD) as superbases and secondary alkylamines or α-aminoacids as NH donors [108,109,110,111,112,113,114,115]. These reactions can be carried out by exposing a neat superbase/*N*-donor 1:1 mixture to CO_2_ atmosphere or by using classical inert organic solvents. In principle, either two components of the superbase/amine/CO_2_ system can be preliminarily mixed and then allowed to react with the third component, as exemplified by the case of morpholine (Scheme 10) [112,114].

**Scheme 11:**
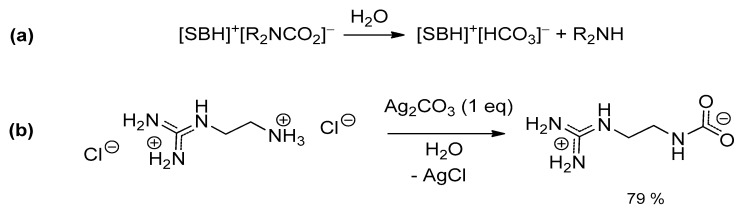
Hydrolysis of amidinium/guanidinium carbamates (SB = superbase) (**a**); synthesis of (2-amminoethyl)guanidine carbamate (**b**).

It appears that the sensitivity to hydrolysis as well as the overall stability (and associated “switchable” properties) of amidinium/guanidinium carbamates is very much dependent on the superbase/amine combination. In some cases, the “water tolerance” has been referred to the persistence of the (ionic) liquid phase after exposure of the system to air/moisture, even though the formation of bicarbonates was readily observed (Scheme 11a) [105,107].

It is noteworthy that (2-ammoniumethyl)guanidinium dichloride reacts with Ag_2_CO_3_ directly in water, affording the corresponding guanidinium carbamate in high yield (Scheme 11b) [116]. This is a rare example of indirect generation of a carbamate (i.e., not using CO_2_) and where the superbase and the amine belong to the same molecule. An extensive network of H-bonding is present in the solid state structure, presumably contributing to the stabilization of the system (vs. bicarbonate formation).

**Scheme 12:**
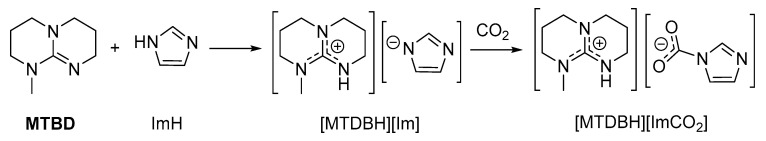
Reaction of MTBD and imidazole, affording the “protic ionic liquid” [MTDBH][Im], and the subsequent CO_2_ uptake in the form of a carbamate.

The high proton affinity of superbases can stabilize carbamates formed by NH-donors more acidic (and thus less Lewis basic) than alkylamines. In this setting, 1:1 mixtures of amidines/guanidines and azoles or pyrrolidone react in a Brønsted acid/base fashion, forming “protic ionic liquids” (PILs) (Scheme 12). These systems are capable of absorbing significant amounts of CO_2_ (generally ≤ 1 equivalent) and spectroscopic data (IR, ^13^C-NMR) agree with the formation of carbamates [117,118,119,120,121].

In conclusion, the study of the interaction of superbases with CO_2_ has led to the isolation of unique zwitterionic carbamato adducts and has highlighted new pathways for CO_2_ activation. Importantly, the synergistic role of amidines/guanidines in combination with ordinary alkylamines (or other NH-donors) allows the stabilization of a wide range of carbamates and enables their use for stoichiometric and catalytic CO_2_ transfer reactions.

## 3. Synthesis, Structure and Reactivity of Metal Carbamates

**Scheme 13:**
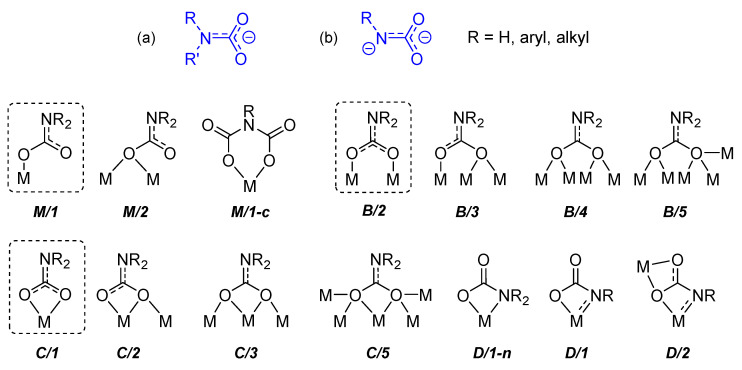
General structure of a carbamato (**a**) and a carbamyldiide (**b**) ligand and coordination motifs observed in solid-state structures (the most common ones are circled).

Carbamato ligands, as previously defined [42], are monoanionic species with general formula R_2_NCO_2_^(−)^ (R = H, alkyl or aryl group), resulting from the combination of carbon dioxide with ammonia or most frequently primary/secondary amines. Such anions usually behave as *O*-donors towards metal centers, giving rise to metal carbamato complexes (Scheme 13a). In the present review, we will adopt a general definition of carbamato ligands, which includes those derived from other *N*-donors (e.g., pyridines and related systems) and also dianionic carbamyldiide species, RN^(−)^CO_2_^(−)^ (Scheme 13b).

Carbamates are versatile ligands, offering a wide variety of coordination modes to metal centers, as recognized in solid state structures. The most frequent coordination modes are those wherein the carbamato ligand is bonded to a metal center in monodentate (***M/1***) or chelating (***C/1***) fashion or is bridged between two metal centers (***B/2***). Other possibilities (***C/3***, ***C/5***, ***B/3***, ***B/4*** and ***B/5***) arise from binding additional metal(s) per oxygen atom. Special coordination modes are available to dianionic carbamyldiide ligands, involving the nitrogen atom in the coordination (***D/1*** and ***D/2***).

Over 380 publications describing the preparation and/or application of circa 1000 metal carbamato complexes have appeared in the literature hitherto, some of them described in 2003 [42]. Herein, we will present a concise but comprehensive description of the preparative methods, structures and reactivity of metal carbamato complexes, with specific reference to the most recent results and novelties.

The first Section 3.1 gives an overall description of the preparative routes and structural aspects of homoleptic metal carbamato complexes, i.e., coordination compounds possessing only carbamato ligands within their coordination sphere. The second Section 3.2 describes the synthetic methodologies employed to introduce (a) carbamato ligand(s) on a generic metal scaffold, thus covering ‘heteroleptic’ metal complexes. The third Section 3.3 is dedicated to the dynamics and reactivity of carbamato complexes, taking homoleptic complexes as prototypical examples. The final Section 3.4 focuses on spectroscopic and crystallographic data related to carbamato ligands and their coordination modes.

### 3.1. Homoleptic Carbamato Complexes

**Scheme 14:**
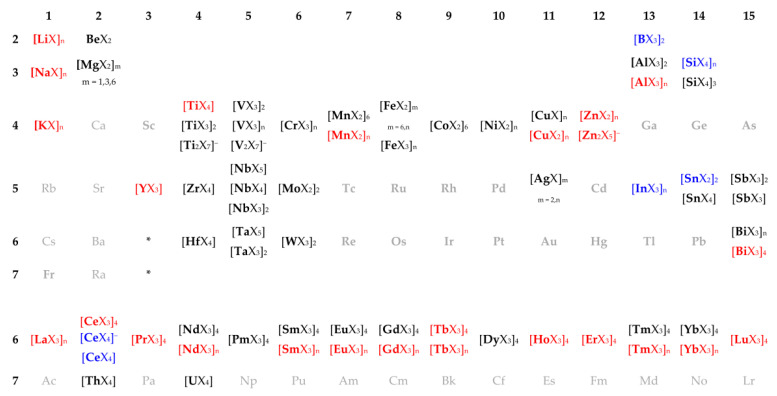
Periodic table of homoleptic carbamato complexes [MX_n_]_m_^0/−^ (X = O_2_CNR_2_; R = H or alky/aryl group); only isolated and characterized compounds are included. Examples not covered in the previous review are highlighted in red (new compounds of already-known types) or in blue (first metal carbamate ever reported). n = unknown nuclearity or polymeric.

Homoleptic carbamato complexes have been reported for a great number of metallic (or semimetallic) elements in the periodic table (Scheme 14). The vast majority of such derivatives are neutral species, complemented by few anionic complexes, and can be represented with the general formula [M(O_2_CNR_2_)_n_]_m_^0/−^, where m represents the nuclearity of the system. Homoleptic carbamato complexes are generally associated to the most common oxidation state for M; moreover, they have also been reported for the same metal in different oxidation states (e.g., Ce, Ti, Nb, Ta, Sn). Their distribution in the periodic table reflects a preference for ‘hard’ oxophilic metal centers, being carbamates effective *O*-donor ligands. However, it has to be considered that a more extended coverage of metals and oxidation states is achieved including a suitable ligand in the coordination sphere, e.g., as for mixed chlorido-carbamato or amino-carbamato complexes (see Section 3.2) [42].

**Scheme 15:**
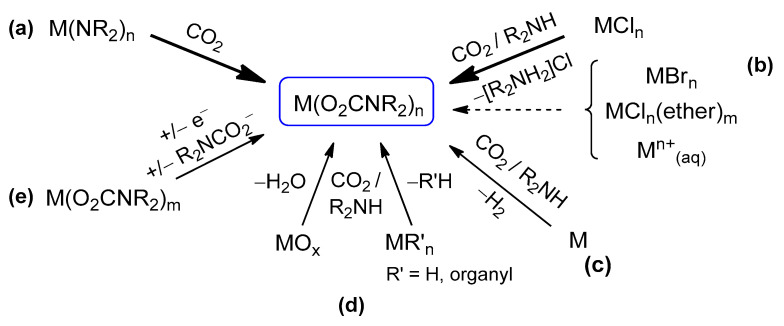
Synthetic routes to homoleptic metal carbamato complexes: (**a**) carbonation of a metal amide, (**b**) ligand substitution reaction from a metal chloride or related species, (**c**) reaction of CO_2_/R_2_NH with oxidation of elemental metal, (**d**) reaction of CO_2_/R_2_NH with a metal oxide or a metal hydride/organyl, (**e**) redox reaction and/or ligand transfer.

Preparative methods. The main synthetic routes to homoleptic metal carbamato complexes are outlined in Scheme 15. They encompass different reactivities, often relying on the in situ generation of the carbamato ligand from the amine/CO_2_ system. The possibility and convenience to use one method or another depend on the availability and reactivity of the required metal precursor as well as solubility issues. The various methods are described below, with selected examples taken from the recent literature.

**Scheme 16:**
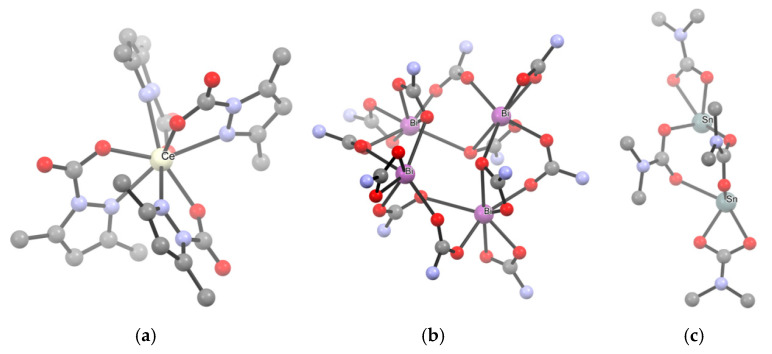
View of the X-ray crystal structure of (**a**) [Ce(O_2_CMe_2_pz)_4_], (**b**) [Bi(O_2_CN*^i^*Pr_2_)_3_]_4_ (^i^Pr groups omitted for clarity), (**c**) [Sn(O_2_CNMe_2_)_2_]_2_. Drawings based on published data [134,135,136], H atoms omitted for clarity.

The first method entails the carbonation of a metal amide (Scheme 15a). Indeed, [Ti(O_2_CNMe_2_)_4_], the first carbamato complex to be reported, was obtained by exhaustive carbonation of the *N*,*N*-dimethylamido complex of titanium(IV), Ti(NMe_2_)_4_ [122]. Since then, the M(NR_2_)_n_/CO_2_ route has been successfully employed in the preparation of homoleptic complexes of the main group metals [42,123], early transition elements [124,125,126,127,128,129,130,131], uranium(IV) and thorium(IV) [132]. In 2010, Kennedy et al. applied this methodology to obtain Li^+^, Na^+^ and K^+^ 2,2,6,6-tetramethylpiperidine (TMP) carbamates [133]. A few years ago, the same method was applied to synthesize homoleptic cerium carbamates [Ce_3_(O_2_CMe_2_pz)_3_]_4_ and [Ce(O_2_CMe_2_pz)_4_] (Me_2_pz = 3,5-dimethylpyrazole) from the respective amides [Ce(Me_2_pz)_3_]_4_ and [Ce(Me_2_pz)_4_]_2_ (Scheme 16a) [134]. These compounds are peculiar in that they contain only ligands of the same type (hence they are ‘homoleptic’) but one nitrogen atom of the pyrazole ring is also involved in coordination (vide infra).

Concerning p-block metals, carbonation of metal amides was recently used to obtain [Bi(O_2_CN*^i^*Pr_2_)_3_]_4_ from Bi(N*^i^*Pr_2_)_3_ [135] and [Sn(O_2_CNMe_2_)_2_]_2_ from Sn(NMe_2_)_2_ [136] (Scheme 16b,c). Most notably, these complexes represent the first examples of structurally characterized bismuth carbamate and homoleptic Sn(II) carbamate, respectively.

The instability of some metal *N*,*N*-dialkylamides and the difficulties in their preparation, particularly with aryl or complex alkyl groups, prompted the development of alternative synthetic methods. Thus, in 1978, Calderazzo et al. reported the synthesis of uranium(IV) *N*,*N*-dialkylcarbamates, [U(O_2_CNR_2_)_4_], starting from the corresponding anhydrous metal chloride as precursor and NHR_2_ saturated with CO_2_ as the carbamato source [137]. Since then, the R_2_NCO^−^/Cl^−^ metathetical reaction (Scheme 15b) has been employed for the synthesis of a vast number of metal carbamates, including heteroleptic derivatives (see Section 3.2), and can be regarded as the most general synthetic method [42,138]. In the case of secondary amines, the reaction is conveniently carried out in toluene or aliphatic hydrocarbons, wherein the insoluble dialkylammonium chloride byproduct can be easily filtered off, leaving a solution of the metal carbamato product.

**Scheme 17:**
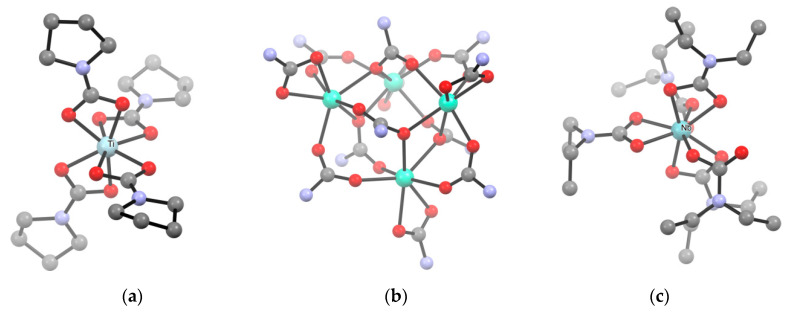
View of the X-ray crystal structure of (**a**) [Ti(O_2_CNPyr)_4_] (Pyr = pyrrolidine), (**b**) [Ln(O_2_CN^i^Pr_2_)_3_]_4_ (Ln = La, Pr, Nd, Sm, Eu, Gd, Dy, Ho, Er, Yb, Lu) (*^i^*Pr groups omitted for clarity), (**c**) [Nb(O_2_CNEt)_5_]. Drawings based on published data [23,144,145,146,147,171], H atoms omitted for clarity.

Recent examples of homoleptic carbamates obtained by ligand substitution from the respective metal chlorides include [Ti(O_2_CNPyr)_4_], being the first pyrrolidine-based metal carbamate (Scheme 17a) [139], [Cu(O_2_CN(allyl)_2_)_2_] [140], [NH_2_^i^Pr_2_][B(O_2_CN^i^Pr_2_)_4_] and [B_2_(O_2_CN^i^Pr_2_)_6_] [141] and [In(O_2_CNEt_2_)_3_] [142].

Drawbacks along the MCl_n_/R_2_NH/CO_2_ route may occur in the case of poorly soluble, unreactive metal chlorides, and can be overcome by employing metal bromide or metal chloride adducts with labile ligands, such as MCl_n_(DME)_m_ or MCl_n_(THF)_m_, as precursors. For instance, MnCl_2_(THF)_1.6_ and FeCl_2_(THF)_1.5_ were used in the syntheses of [M(O_2_CNEt_2_)_2_]_6_ and [M(O_2_CN^i^Pr_2_)_2_]_n_ (M = Fe, Mn) [143]. In the case of lanthanides, the use of LnCl_3_(ether)_x_ allowed straightforward preparation of [Ln(O_2_CN^i^Pr_2_)_3_]_4_ (Scheme 17b) and [Ln(O_2_CNBu_2_)_3_] (Ln = La, Ce, Pr, Nd, Sm, Eu, Gd, Dy, Ho, Er, Yb, Lu) [23,144,145,146,147].

When high valent metal centers and amines bearing alkyl chains longer than C_2_ are involved, activation of the amine and reduction of the metal may be observed. Thus, during the purification of Nb(O_2_CNEt_2_)_5_ from hot heptane, the pale yellow mixture turned blue with evolution of CO_2_ and Nb(O_2_CNEt_2_)_4_ was isolated in high yield. The analogous thermal treatment of Nb (O_2_CNMe_2_)_5_ afforded only small amounts of reduction product after 48 h at circa 100 °C [131]. In agreement with the generally observed higher stability of the higher oxidation states going down a group of transition elements, Ta(O_2_CNEt_2_)_5_ does not undergo appreciable reduction to Ta (IV) under the same conditions.

The observed thermal behavior of M(O_2_CNR_2_)_5_ parallels that of the Nb(V) and Ta(V) amides, M(NR_2_)_5_: these species are stable at ambient temperature in the case of tantalum [148], but easily reduce to the +4 oxidation state in the case of niobium [149], the reduction extent increasing on increasing the length of the alkyl group. Both steric and electronic effects play an important role [149].

Even bare aquo-complexes can be used as precursors for the preparation of homoleptic complexes by ligand substitution [150]. In this regard, Armelao, Belli Dell’Amico and co-workers reported, in 2014, an innovative method for the preparation of [Ln(O_2_CNBu_2_)_3_]_n_ (Ln = Nd, Eu, Tb) [151]. In this procedure, the preformed ammonium carbamate in heptane was used to extract the metal ion from an aqueous solution of Ln^3+^ (obtained by dissolving Ln_2_O_3_ in HCl). The rapid formation of the carbamato complex and the balanced lipophilicity provided by the amine allowed its extraction in the organic layer while retaining the [R_2_NH_2_]Cl co-product in the aqueous phase. The extraction method was later extended to Ce(III) [152], Tm(III) [153] and Y(III) [154] carbamato complexes and to the hetero-trimetallic derivative [Tm_3/4_Tb_3/4_Eu_3/4_(O_2_CNBu_2_)_12_] [153].

In some cases, homoleptic complexes can be prepared directly from the elemental metal and the R_2_NH/CO_2_ system in coordinating solvents (e.g., THF, acetonitrile) (Scheme 15c). This methodology is effective for alkali, alkali-earth metals [155,156] and zinc [157]. Clearly, these reactions may proceed through the formation of the metal amide in situ. A recent example of this reactivity is represented by [Zn(O_2_CNH*^i^*Bu)_2_], obtained by treating a suspension of Zn powder in 2-methoxyethanol with a stoichiometric amount of [*^i^*BuNH_3_][O_2_CNH*^i^*Bu] [158]. In this context, we also mention the preparation of the non-homoleptic [Cu(O_2_CNMe_2_)(Me_2_NH)_2_] from copper metal and [NMe_2_H_2_][O_2_CNMe_2_], in the presence of O_2_ as external oxidant [157].

Alternative precursors of homoleptic complexes are metal oxides [159], alkoxides [159], metal alkyls, Grignard reagents [160,161,162] and MnCp_2_ [163] (Scheme 15d). All these species react with amines and CO_2_ under different conditions, by combining an acid/base reaction with the coordination of the in situ generated carbamato ligand.

In this regard, silver carbamates can be prepared from a suspension of Ag_2_O, treated with amine under CO_2_ atmosphere [159]. This method has not a general applicability, due to unfavorable thermodynamics, but it is not unique to Ag_2_O. Both the neutral polymeric [Zn(O_2_CNMe_2_)]_n_ and the dimeric anionic carbamate [Me_2_NH_2_][Zn_2_(O_2_CNMe_2_)_5_] were isolated from the reaction of ZnO with [Me_2_NH_2_][O_2_NMe_2_] in MeCN [164]. However, the same reactions did not work with other dialkylamines.

Concerning metal alkyls, lithium *N*,*N*-dialkylcarbamates were recently prepared starting from *^n^*BuLi and CO_2_ in the presence of diisopropylamine or pyrrole [165]. A related reaction was reported with NaH, providing a more convenient pathway to Na(O_2_CNEt_2_) compared to the use of sodium sand [166]. Additionally, in these cases, intermediacy of the in situ formed metal amide is conceivable.

Finally, a preformed carbamato complex can be exploited to obtain homoleptic derivatives (Scheme 15e). This is related to trans-metalation procedures [167] or redox reactions, the latter possibly accompanied by ligand transfer (e.g., reduction of Nb^V^, Ta^V^ or Ti^IV^ to lower-valent derivatives of Nb^IV^, Ta^III^ and Ti^III^, respectively) [131,167,168,169,170] (Scheme 15e).

Structural aspects. Homoleptic metal carbamates exhibit a wide range of nuclearities in the solid state, ranging from monometallic complexes to oligomeric and even polymeric structures. The aggregation is realized by bridging coordination of the carbamato moieties, as well as metal-metal bonding in some cases, and can be regulated by the nitrogen substituents, with bulkier groups usually favoring a lower degree of aggregation. For instance, [Ti(O_2_CNR_2_)_4_] (R = Me, Et, ^i^Pr, pyrrolidine; Scheme 17a) and [Nb(O_2_CNR_2_)_5_] (R = Me, Et; Scheme 17c) are examples of mononuclear complexes featuring only chelating (***C/1***) or chelating (***C/1***) and monodentate (***M/1***) carbamates, respectively [130,171,172,173,174]. On the other hand, homoleptic diethyl or dimethyl carbamato derivatives of W(III), Mo(II) and Sn(II) (Scheme 16c) are dinuclear, featuring bridging ligands (***B/2***) and the first two complexes also M-M bonding [136,175,176]. The structure of [Bi(O_2_CN*^i^*Pr_2_)_3_]_4_ is tetrameric, each Bi being coordinated by one chelating (**C/1**), one bridging (***B/2***) and one bridging-chelating (***C/2***) carbamato groups in a distorted pentagonal bipyramidal environment (Scheme 16b) [135]. Instead, *N*,*N*-diisopropyl carbamates of Ln(III) (Ln = Pr, Nd, Sm, Eu, Gd, Dy, Ho, Er, Yb, Lu) are isostructural and exhibit a tetrameric structure with C_2_ symmetry and heptacoordinated metal centers (Scheme 17b) [23,145,146,147]. In these complexes, the carbamato ligands adopt three different coordination modes, binding one (***C/1***), two (***B/2***) or three (***B/3***) metal centers, respectively. From the collection of all the presented structures, Belli Dell’Amico et al. highlighted a parabolic trend in the decrease of Ln-O bond distances over the lanthanide series [145]. The only exception to this structural motif is represented by the Ce(III) derivative, showing a less symmetrical structure in which it is possible to observe five different coordination modes (***C/1***, ***C/2***, ***B/2***, ***B/3***, ***B/4***) [144]. This particular arrangement leads to a packing in which the metal atoms are not completely surrounded by the ligands, allowing the favorable oxidation to Ce(IV) by means of O_2_ (vide infra). Hexanuclear structures are adopted by Mg(II), Mn(II) and Co(II) diethyl carbamates [42].

In some cases, the nuclearity of homoleptic complexes in the solid state has not been determined and a polymeric structure was suggested, based on the insolubility in non-coordinating solvents (e.g., benzene or toluene). This feature is common to several alkali metal carbamates [156,177], including those of bulky 2,2,6,6-tetramethylpiperidine [133]. On the other hand, the lithium diisopropyl derivative [(LiO_2_CN^i^Pr_2_)_12_(^i^Pr_2_NCOOH)_2_] is a dodecanuclear cluster decorated with a rare carbamic acid ligand [47,84,178], whose formation has been ascribed to adventitious hydrolysis [165].

Unusual coordination environments have been recognized for cerium 3,5-dimetylpyrazole (Me_2_pz) carbamates, also due to the chelation of the metal center by the non-carbonatated pyrazole nitrogen [134]. The structure of [Ce(O_2_CMe_2_pz)_3_]_4_ comprises a 9-coordinate Ce(III) atom displaying three different coordination modes for the ligands (***M/1***, ***C/2***, ***B/3***), while the corresponding Ce(IV) complex (Scheme 16a) and the anionic Ce(III) derivative [Bu_4_N][Ce(O_2_CMe_2_pz)_4_] are mononuclear based on 8-coordinate cerium center.

### 3.2. Heteroleptic Carbamato Complexes

Heteroleptic metal carbamato complexes are those including additional ligands in the coordination sphere. Such classification encompasses many derivatives that are closely related to the parent homoleptic compounds, such as mixed chlorido-carbamates or amino/amido-carbamates (vide infra). These can be viewed as intermediate products along the preparative routes that (in principle or in practice) lead to the homoleptic congeners. On the other hand, a large number of complexes presents a single carbamato unit. These include most organometallic derivatives or coordination compounds featuring very sophisticated, multidentate ligands. In such cases, the carbamato ligand is ancillary with respect to the properties and reactivity of the compound itself.

**Scheme 18:**
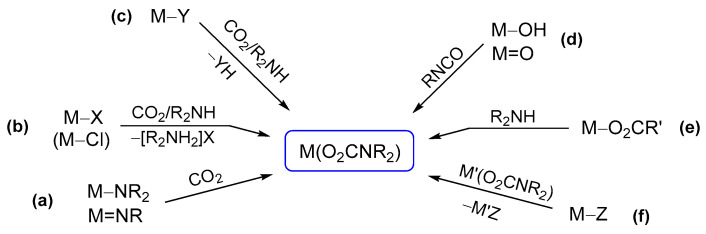
Main synthetic routes for the assembly of a carbamato ligand on metal complexes: (**a**) carbonation of an M−N bond, (**b**) chloride (or another anionic ligand) substitution, (**c**) coupled ligand substitution/proton transfer reaction, (**d**) coupling of a hydroxido (or oxido) ligand with organic isocyanates, (**e**) reaction of an amine with a metal carboxyl complex and (**f**) ligand transfer route.

Given the vast and heterogeneous amount of information, the present discussion will provide an overview of the various synthetic methods available for the introduction of a carbamato ligand (Scheme 18), with selected examples taken from the most recent literature. This approach will highlight the applicability and limitations of each method along the periodic table.

**Scheme 19:**
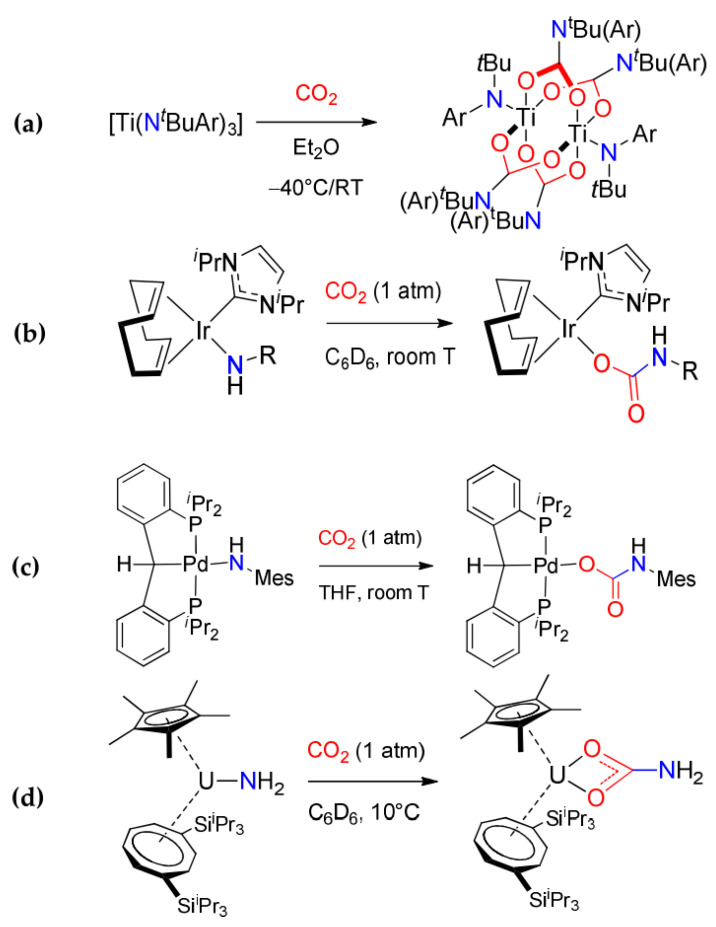
Syntheses of metal carbamates obtained by carbonation of amido ligands: (**a**) dinuclear Ti(III) amido-carbamates obtained by partial carbonation of the homoleptic amides, (**b**) organometallic Ir(I) carbene, (**c**) Pd(II) PCP-pincer and (**d**) primary U(IV) carbamate complexes.

Carbon dioxide insertion into M−N bonds (Scheme 18a). The reaction of an alkylamido group with CO_2_, leading to the generation of a carbamato ligand, has found widespread use for s-block [42] and early d-transition metals [125,126,128,129,130,179]. In this regard, new fascinating examples have been recently reported [133,180,181,182,183,184,185,186]. The simplest cases are represented by homoleptic metal amido complexes undergoing selective carbonation, leading to mixed amido-carbamato derivatives. For instance, Cotton et al. reported the partial carbonation of Ti(III) amido, resulting in dimeric amido-carbamato compounds, showing diamagnetic behavior due to anti-ferromagnetic coupling [180] (Scheme 19a).

On the other hand, the insertion of CO_2_ into M−N bonds was only sparingly applied to lanthanides, actinides and late transition metals [132,187,188,189] in the beginning. The scenario changed over the last 15 years, as several carbamato complexes obtained by this methodology were reported, and especially organometallic species. These include late d-block metals such as iridium [190,191], nickel [192,193,194,195,196,197], palladium [198,199], gold [200] and zinc [201,202,203,204], f-block metals cerium [134] and uranium [205,206,207,208,209] and p-block metals tin [210] and gallium [211,212]. Furthermore, in situ-formed amide complex of Zn(II) and alkyl-amide of Mg(II) were mixed under carbon dioxide atmosphere to afford a heterobimetallic Zn/Mg alkyl-carbamato derivative [213]. Selected cases are represented in Scheme 19b–d, including a rare type of a primary carbamato ligand in the U(IV) derivative [Cp*(COT^TIPS2^)U(O_2_CNH_2_)] [206].

**Scheme 20:**
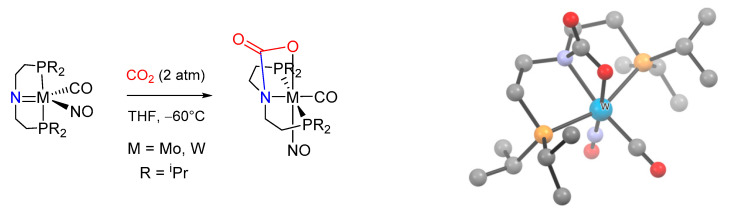
Carbonation of the diphosphino amide ligands within Mo(0) and W(0) complexes, resulting in the unusual *N*,*O*-bound carbamate ligand, and crystal structure of the tungsten isopropyl derivative (H atoms omitted for clarity). Drawing based on published data [185].

The generally accepted mechanism [214,215] for low-valent d-transition metals involves a direct nucleophilic attack of the metal-coordinated nitrogen to carbon dioxide, with the generation of an intermediate *N*-bound carbamato/carbamic acid moiety. Subsequent rearrangement provides the typical *O*-coordinated carbamato ligand [187,188,191,192,216]. A further confirmation was recently reported for Mo(0) and W(0) complexes bearing a diphosphino amide pincer ligand (Scheme 20) [185]. In this case, the geometry of the ligand forces the nitrogen atom to remain coordinated after CO_2_ addition, thus stabilizing a rare example of monoanionic *N*,*O*-chelating carbamato ligand (***D/1-n***).

Some of the late transition metal complexes featuring a monodentate carbamate are quite unstable towards decarboxylation, either in solution or in the solid state, especially under vacuum [190,191,192,198,200]. Among other factors, the relatively strong metal-nitrogen bond may contribute to the reversibility of the CO_2_ insertion process.

**Scheme 21:**
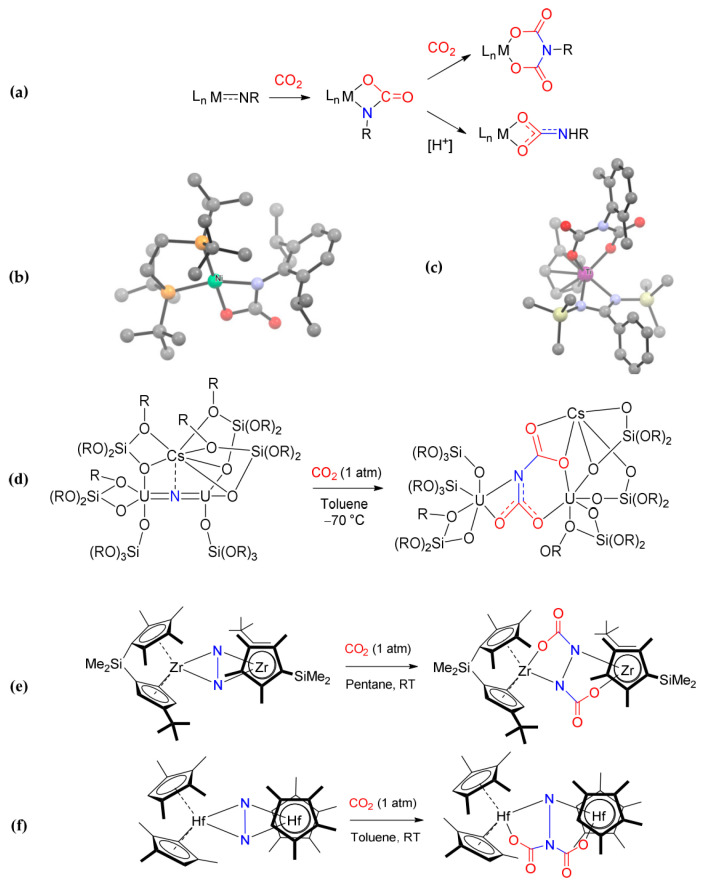
Insertion of CO_2_ into different M−N bonds: (**a**) general reactivity scheme for a metal imido complex with CO_2_ and crystal structures of [Ni(ditbpe)(κ^2^*N*,*O*-O_2_CNXyl)] (**b**) and [Ti(η-C_5_H_4_Me){(O_2_C)_2_N(-2,6-C_6_H_3_Me_2_)}{PhC(SiMe_3_)_2_}] (**c**); double carbonation of a diuranium bridging nitride ligand (**d**); metal dinitrogen metallocene complexes of zirconium (**e**) and hafnium (**f**). Drawings based on published data [220,221,225,227,228], H atoms omitted for clarity.

The insertion of CO_2_ into M−N bonds has also been reported for Sc(III) [217], Ti(IV) [218,219,220], Ni(II) [221], Pd(II) [222] and Ir(III) [223,224] imido complexes. The dianionic carbamyldiide ligand thus generated usually remains coordinated to the metal via N,O (Scheme 21a). Additionally, in this case, a mechanism involving the direct nucleophilic attack of nitrogen to carbon dioxide appears to be favored over a [2 + 2] cycloaddition [185,222]. The cyclic metallacarbamato ligand may undergo further reactions, i.e., a second CO_2_ insertion to produce a bis-carbamato (azadicarboxylato) ligand [220,221] or proton abstraction from another ligand, affording an ordinary carbamato ligand [224].

Formation of exotic polyanionic carbamato ligands was observed by addition of CO_2_ to a nitrido-bridged diuranium(IV) complex [225,226], and to zirconocene and hafnocene dinitrogen complexes [227,228] (Scheme 21). These systems are prone to double CO_2_ insertion, either on the same or on different nitrogen atoms [229].

Ligand substitution reactions (Scheme 18b). Partial ligand substitution along the MCl_n_/CO_2_/R_2_NH pathway gives access to mixed species such as metal chlorido-carbamates or amino-chlorido-carbamates [42]. For instance, the reaction of ZnCl_2_ with Et_2_NH/CO_2_ in THF afforded [Zn_2_(μ-O_2_CNEt_2_)_3_Cl(Et_2_NH)], displaying a paddlewheel structure with bridging (***B/2***) carbamato ligands [230]. A very recent example of partial substitution from a metal chloride is the reaction of TiCl_4_ with one equivalent of preformed [TMG][O_2_CNEt_2_] (TMG = tetramethylguanidine), leading to the trinuclear [TiCl_2_(O_2_CNEt_2_)_2_]_3_ [109]. Interestingly, the co-product is not the expected guanidinium chloride but the hexachlorometalate [TMG]_2_[TiCl_6_] (probably formed via addition of chlorides to unreacted TiCl_4_), which can be easily removed by filtration.

**Scheme 22:**
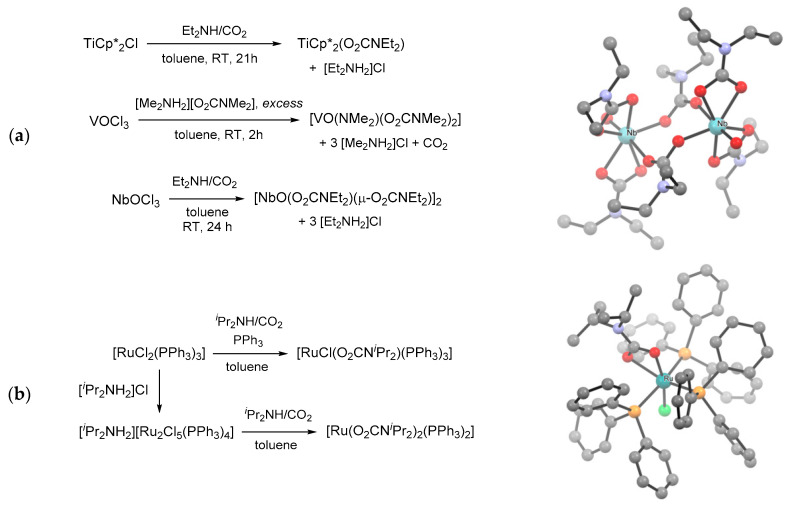
Metal carbamato complexes obtained by Cl^−^/R_2_NCO_2_^−^ exchange: reactions of TiCp*_2_Cl, V(V) and Nb(V) oxido-chlorides with amines/CO_2_ and X-ray structure of [NbO(O_2_CNMe_2_)_3_]_2_ (**a**); preparation of *mer*-[RuCl(O_2_CN^i^Pr_2_)(PPh_3_)_3_] and [Ru(O_2_CN^i^Pr_2_)_2_(PPh_3_)_2_] from Ru(II) chloride precursors and X-ray structure of the former (**b**). Drawings based on published data (H atoms omitted for clarity) [171,231].

More in general, metal chlorido complexes can be used as precursors for the installation of carbamato unit(s) [166,171,231,232,233]. In this setting, treating TiCp*_2_Cl, VOCl_3_ and NbOCl_3_ with pre-carbonated amines respectively allowed the isolation of [TiCp*_2_(O_2_CNEt_2_)] [234], [VO(NMe_2_)(O_2_CNMe_2_)_2_] [166] and [NbO(O_2_CNEt_2_)_3_]_2_ [171] (Scheme 22a). Similarly, ruthenium(II) complexes *mer*-[RuCl(O_2_CN^i^Pr_2_)(PPh_3_)_3_] and [Ru(O_2_CN^i^Pr_2_)_2_(PPh_3_)_2_] were prepared by chloride/carbamate exchange from [RuCl_2_(PPh_3_)_3_] and the chloro-bridged dimer [Ru_2_Cl_5_(PPh_3_)_4_]^−^, respectively (Scheme 22b) [231].

**Scheme 23:**
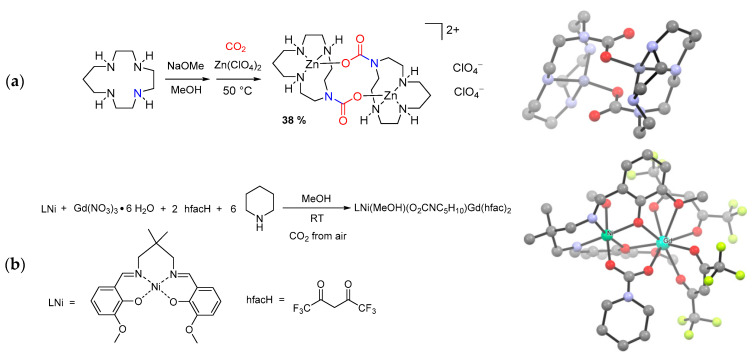
Synthesis and views of the X-ray crystal structures of: (**a**) a Zn(II) macrocyclic carbamate obtained from [13]aneN4 (1,4,7,10-tetra-azacyclotridecane), Zn(ClO_4_)_2_ and CO_2_; (**b**) a heterobimetallic Ni/Gd complex with bridging morpholine carbamato ligands. Drawing based on published data (H atoms omitted for clarity) [242,246].

Other metal compounds, such as Dy(III) [235], Fe(II) [236], Cu(II) [237,238,239,240,241], Ni(II) [239] and Zn(II) [240,241,242,243] perchlorates, nitrates and sulfates, uranyl diacetate [244] or [Pd(MeCN)_4_]^2+^ [245] were used as starting materials for the preparation of carbamato derivatives. The reactions are usually carried out in polar organic solvents (MeOH, MeCN) in the presence of amines, polyamines or aza-macrocyclic ligands under CO_2_ atmosphere (Scheme 23a). Most importantly, some cases of CO_2_ fixation directly from ambient air have been reported [233,235,237,238,239,241,244,246,247].

The combination of two different salts allowed the preparation of heterobimetallic 3d/4f carbamato compounds, also in a one-pot fashion (Scheme 23b) [246,247]. Such compounds aroused great interest within the scientific community for their magnetic properties.

**Scheme 24:**
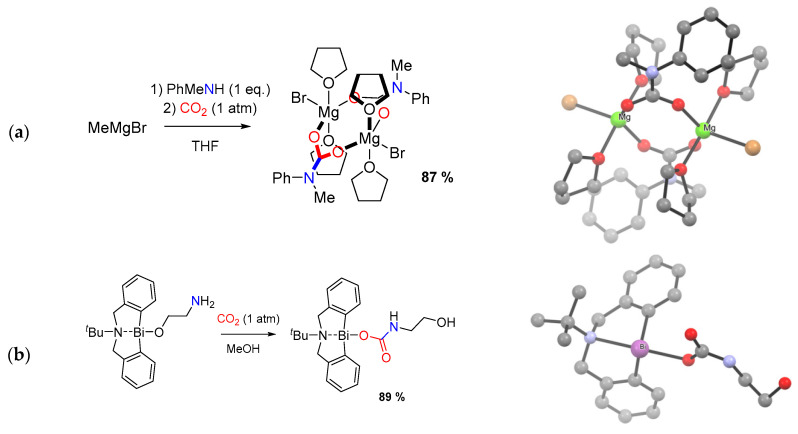
Synthesis and views of X-ray structures of: (**a**) dinuclear Mg carbamate from Grignard reagent; (**b**) Bi(III) carbamato complex from carbonation of amino-alkoxide ligand. Drawings based on published data (H atoms omitted for clarity) [248,250].

Coupled ligand substitution/proton transfer reactions (Scheme 18c). In principle, a metal complex bearing a ligand that can be removed upon protonation by amines (or in situ formed carbamic acids/ammonium carbamates) can be a precursor for the installation of a carbamato ligand. Organolithium compounds, Grignard reagents and other d-block and p-block alkyls can be employed to this purpose, hence the generation of the carbamate may proceed through the intermediacy of a metal-amide unit [42]. These reactions are usually conducted in coordinating solvents (e.g., THF, Et_2_O), which are often found incorporated in the final complex [165,248]. For instance, the reaction of MeMgBr with *N*-methylaniline and CO_2_ in THF leads to the dimer [Mg(O_2_CN(Me)Ph)(THF)_2_Br]_2_, where magnesium shows a trigonal bipyramidal coordination geometry [248] (Scheme 24a).

The use of metal oxides or oxido/alkoxido complexes as precursors, although less frequent, may also be included in this category [42]. For instance, octanuclear carbonato-carbamates of general formula [Me_2_NH_2_]*_n_*[Mg_8_(CO_3_)_2_(O_2_CNMe_2_)_(12+*n*)_] (n = 0–3) were obtained by treating magnesium oxide with [Me_2_NH_2_][O_2_CNMe_2_] in toluene, the value of n depending on the reaction conditions. In particular, [Me_2_NH_2_]*_3_*[Mg_8_(CO_3_)_2_(O_2_CNMe_2_)_15_] converted into [Mg_8_(CO_3_)_2_(O_2_CNMe_2_)_12_] (n = 0) by heating under vacuum [249]. A basic example of the present synthetic procedure is provided by a Bi(III) complex with an amino-alkoxido ligand [250]: carbonation of the pendant amino group is followed by intramolecular proton transfer and ligand slippage, yielding the carbamato moiety (Scheme 24b).

**Scheme 25:**
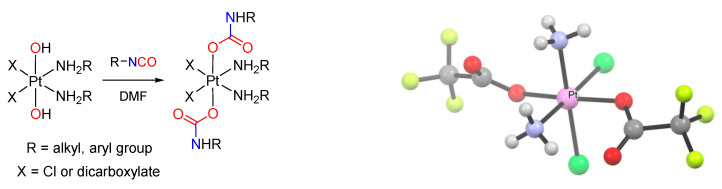
General reaction of a Pt(IV) (bis)hydroxo species with alkyl/aryl isocyanate affording a Pt(IV) (bis)carbamate and view of the X-ray crystal structure of *cis*,*cis*,*trans*-[PtCl_2_(NH_3_)_2_(O_2_CNCF_3_)_2_]. Drawing based on published data [260].

Reaction of hydroxido(oxido) ligands with organic isocyanates (Scheme 18d). The reaction of a M–OH (or M=O) moiety with an organic isocyanate represents a further possibility to build a carbamato ligand. In the past, only a few metal carbamato complexes were obtained by this route, namely Hg(II) [251], Os(II) [252] and Pt(IV) [253] derivatives. Recently, the family of metal carbamates generated in this way have greatly expanded, including lanthanide compounds [254,255], Ti(IV)-oxido [220], Re(I)-carbonyl [256], Co(III)-pentamine [257] and Ni(II) pincer complexes [258,259]. Moreover, this method has gained increasing importance in the preparation of Pt(IV) compounds (Scheme 25). More precisely, coupling of a Pt(IV) (bis)hydroxo species with an alkyl or aryl isocyanate affords the corresponding (bis)carbamate. Following optimization [260], allowing the reaction to be conducted in dimethylformamide and not in neat isocyanate, more than 60 different Pt(IV) species were thus prepared, arousing interest as possible anticancer agents (see Section 5) [261,262,263,264,265,266,267,268].

**Scheme 26:**
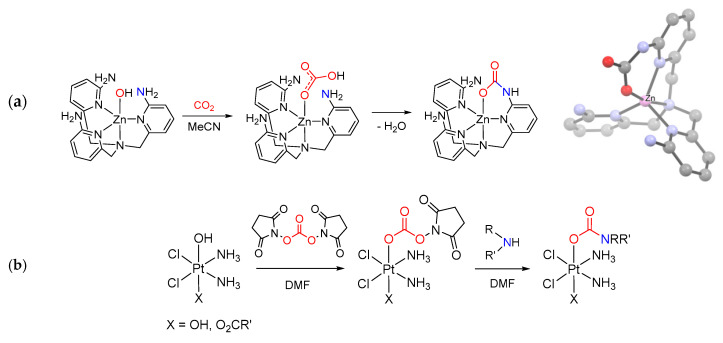
Metal carboxyl complexes as precursors of metal carbamates. (**a**) Formation and view of the X-ray crystal structure of a Zn(II) carbamate complex containing a *N*,*N*,*N*,*N*-tetradentate ligand, via intramolecular amine/HCO_3_ reaction. Drawing based on published data (H atoms omitted for clarity) [269]. (**b**) Reaction of *cis*,*cis*,*trans*-[PtCl_2_(NH_3_)_2_(OH)X] with bis(2,5-dioxopyrrolidin-1-yl)carbonate followed by amine addition to the activated ester.

Reaction of an amine with a metal carboxyl complex (Scheme 18e). Another type of synthesis comprises the reactions of amines (or *N*-donors in general) with a metal carboxyl complex, i.e., bicarbonate [269], formate [270,271], carbonate ester [272] or a CO_2_ adduct directly [273]. As an example, the intramolecular attack of an amine group belonging to a tetradentate pyridylamine ligand onto a Zn(II)-HCO_3_ species resulted in carbamate generation [269] (Scheme 26a). In this regard, a novel synthetic approach consisting in the addition of amines to a Pt(IV)-carbonate ester has been recently proposed (Scheme 26b). This method offers the opportunity for preparing a number of Pt(IV) carbamato derivatives, including some related to secondary amines, thus extending the scope provided by the hydroxide/isocyanate coupling [272].

**Scheme 27:**
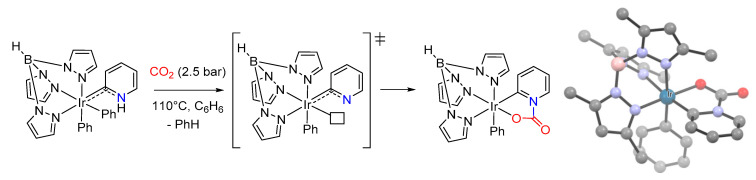
Synthesis and view of the X-ray crystal structure of Ir(Tp) carbamate. Drawing based on published data (H atoms omitted for clarity) [274].

Other methods. Within the plethora of organometallic complexes, another strategy for the installation of a carbamato ligand relies upon the in situ generation of suitable unsaturated fragments [274,275,276]. For instance, reductive elimination of benzene from a phenyl Ir(III) trispyrazolyl borate compound generated a 16e^−^ intermediate capable of incorporating CO_2_ as a carbamate, with the aid of an ancillary metallapyridine ligand (Scheme 27) [274]. A closely related example is represented by the addition of indole-1-carboxylic acid on a formally 16e^−^ Cp*Ir(III) amido complex [277].

A peculiar ligand-assisted addition of CO_2_ was reported for a Fe(II) compound containing a pyridyl-amine ligand. In this case, carbonation of the amino group belonging to a bidentate ligand resulted in the formation of an eight-membered pyridyl-carbamate. The reaction is readily reversible by heating [278].

The formation of a heterodinuclear Fe/In carbonyl-carbamato complex was reported starting from an iron carbonyl-carbamoyl precursor by reaction with InMe_3_. The oxygen transfer to the carbamoyl ligand forming the carbamate is possible due to the decomposition of a second carbamoyl unit [279].

**Scheme 28:**
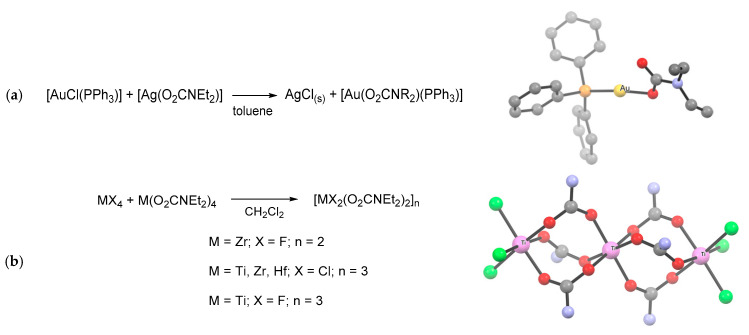
Synthesis and views of X-ray crystal structures of metal carbamato complexes prepared by ligand transfer reactions: (**a**) [Au(PPh_3_)(O_2_CNEt_2_)] obtained by trans-metalation with silver carbamate; (**b**) combination of Group 4 metal halides and their homoleptic carbamates, affording mixed halido-carbamato complexes and structure of [Ti_3_Cl_6_(O_2_CNEt_2_)_6_]. Drawings based on published data (H atoms and Et groups in the Ti complex omitted for clarity) [109,159].

Ligand transfer routes (Scheme 18f). Over the years, the trans-metalation reaction has been extensively employed for the targeted synthesis of various metal carbamates, especially organometallics [42]. Typically, this method exploits silver or alkali metal carbamates as transfer reagents, taking advantage of the precipitation of the metal halide side-product (e.g., NaCl, LiCl, AgCl, AgBr) as the driving force of the reaction (Scheme 28a) [109,133,159,166,212,280]. Attempts to realize trans-metalation based on other combinations of metals may result in carbamato ligand transfer [42] or in the formation of a hetero-bimetallic product [281,282].

Another type of ligand transfer reaction can be performed by mixing equimolar amounts of a metal halide and its corresponding homoleptic carbamate, selectively affording a mixed halido-carbamato complex (Scheme 28b) [109,172].

### 3.3. Dynamics and Reactivity of Metal Carbamato Complexes

**Scheme 29:**
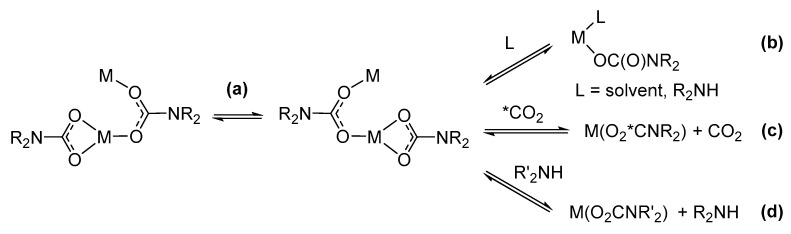
Dynamics of carbamato ligands in solution: (**a**) intramolecular ligand exchange, (**b**) ligand/solvent (reversible) addition, (**c**) CO_2_ exchange with external carbon dioxide, (**d**) transamination reaction.

Carbamato ligand dynamics. Metal carbamates may manifest a dynamic behavior, through the occurrence of processes summarized in Scheme 29. Homoleptic metal carbamato complexes have been widely investigated from this perspective.

Carbamato ligands in metal complexes are usually able to exchange their position and their coordination modes in solution, as demonstrated by variable temperature NMR measurements [127,138,248,283,284,285]. For instance, the two carbamato ligands in [Mg_2_Br_2_(μ-O_2_CNMePh)_2_] are fluxional, rapidly shifting from bridging (***B/2***) to bridging-chelating (***C/2***) mode down to 0 °C, when the exchange turns slow on the NMR time scale [248].

The fluxionality of the carbamato ligand, jointly with the possibility of turning from bidentate to monodentate coordination, permits the entrance of additional ligands in the metal coordination sphere. The possible subsequent decrease in nuclearity of the metal system may lead to dissolution of the otherwise insoluble metal carbamate. The first observation of such a behavior was reported in 1988 by Calderazzo et al. [286]. Some Cu(II) *N*,*N*-dialkylcarbamates, obtained by the typical CuCl_2_/amine/CO_2_ route in heptane, were no longer soluble in the same solvent following their isolation in the solid state. The authors suggested that the initial solubility could be ascribed to the coordination of one or more amine molecules, present in excess in the reaction mixture. Other examples of metal *N,N-*dialkylcarbamates changing their solubility in the presence or absence of amines were later reported [143,287,288]. Analogous equilibria can also explain the solubility of metal carbamates in coordinating solvents [42]. In this regard, THF or TMEDA adducts were recently isolated growing crystals of lithium carbamates [133,165].

Another aspect related to the dynamics of metal carbamato complexes in solution is their ability to interchange the carboxyl unit with external CO_2_ while maintaining their structures intact [42,125,127]. This property was confirmed in 2005 by McCowan and Caudle by ^13^CO_2_ uptake experiments on zinc derivatives [289]. Furthermore, metal carbamates might be susceptible to transamination, exchanging the internal R_2_N group with that coming from an external amine. This feature was exploited for the preparation of [Al(O_2_CNEt_2_)_3_] [290], [Nd(O_2_CNEt_2_)_3_] [145], [Eu(O_2_CNBn_2_)_3_] and [Sm(O_2_CNBn_2_)_3_] [151], taking advantage of the volatility of the outgoing amine and/or the lower solubility of the metal carbamato product.

**Scheme 30:**
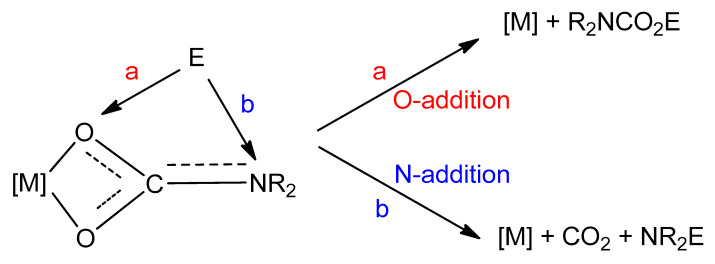
Possible reactions of an electrophile E with a carbamato ligand.

Reactivity with electrophiles. Metal carbamato complexes can be quite reactive towards electrophilic agents. Indeed, carbamato ligands are generally prone to electrophilic attack on the nitrogen or oxygen atom(s). In all cases, the M–O(carbamate) bond(s) is detached, but products vary depending on the type of electrophilic agent, i.e., organic electrophiles or protic species (Scheme 30).

The reactivity with carbon-based electrophiles has been widely studied [42]. The addition of the electrophile to the oxygen atom(s) forms a carbamato ester, while addition to the nitrogen leads to a derivatized amine with release of gaseous CO_2_, Scheme 29a.

**Scheme 31:**
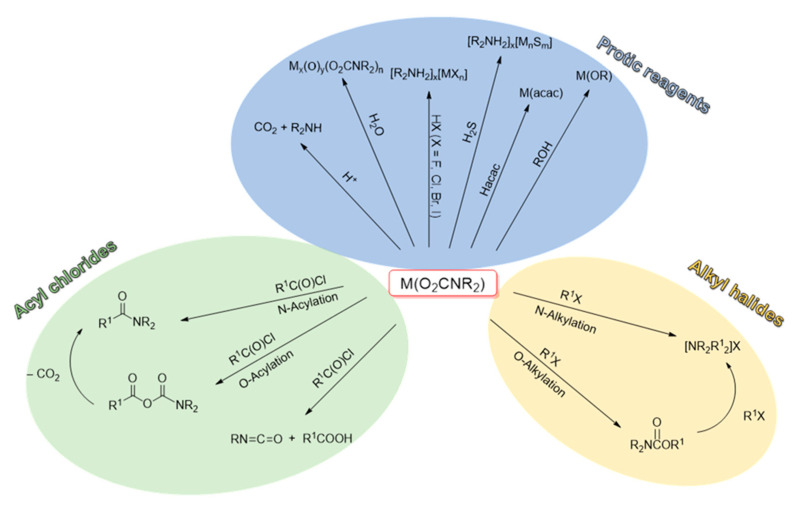
Reactivity patterns for metal carbamato complexes with electrophilic species.

On these grounds, *O*-addition compounds are kinetic products but they are synthetically relevant since their formation represents a net incorporation of carbon dioxide. For instance, stoichiometric reactions with alkyl halides or acyl halides afford the corresponding urethanes and carbamic-carboxylic anhydrides (Scheme 31) [42]. In general, the regioselectivity of these reactions (*O* vs. *N* addition) is variable depending on the system. Urethanes can undergo a second alkylation giving the ammonium salt, while the carbamic-carboxylic anhydride, in the presence of Cu(II) and Fe(III), decomposes affording the corresponding amide via CO_2_ elimination. On the other hand, the use of an *N*-alkylcarbamate in combination with acyl chlorides gives a mixture of isocyanate and carboxylic acid (Scheme 31). A recent example of this reactivity is supplied by the formation of organylsilylurethanes R_n_Si(O_2_CNR′_2_)_(4-n)_ from the reaction of Sn(IV) tetracarbamates with organylchlorosilanes (R_n_SiCl_(4-n)_) [291].

In summary, the carbamato ligand in transition metal complexes is a versatile platform to carry out diverse metal-mediated organic transformations, constituting the conceptual basis for the use of metal carbamates in catalysis. The organic chemistry of carbamato ligands will be covered in more detail in Section 4, with a focus on catalytic processes.

Conversely, the addition of H^+^ from any protic source usually determines the disruption of the carbamato moiety, with the consequent release of the amine and CO_2_. On thermodynamic grounds, the formation of gaseous carbon dioxide (∆G°_f_ = −394.4 kJ/mol at 25 °C) is the driving force for these degradation reactions.

Water is the simplest protic species and hydrolytic reaction(s) have been observed with reference to almost all the categories of carbamato complexes. Therefore, the presence of water is normally undesired, and moisture sensitivity is the “Achille’s heel” of many metal carbamates [42].

**Scheme 32:**
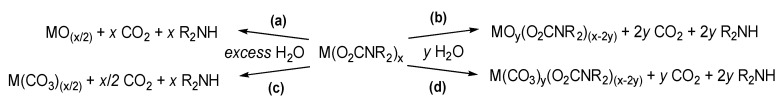
Hydrolytic processes of metal carbamato complexes: exhaustive hydrolysis to metal oxides (**a**) or carbonates (**c**) or partial hydrolysis to oxido-carbamato (**b**) or carbonato-carbamato (**d**) species.

Hydrolysis of metal carbamates in the presence of an excess of water generally leads to the metal oxide (or hydroxide) (Scheme 32a). Some of these oxides, or mixed metal oxides, find relevance in the field of material chemistry, a topic that will be discussed in Section 5. Notwithstanding, the reactivity of carbamato complexes with water can be modulated for preparative purposes, avoiding the exhaustive hydrolysis of the metal-carbamato linkages. Thus, the reactions with a carefully controlled amount of water in organic solvents is exploited for the synthesis of well-defined mixed ligand complexes, such as oxido-carbamato species (Scheme 32b).

**Scheme 33:**
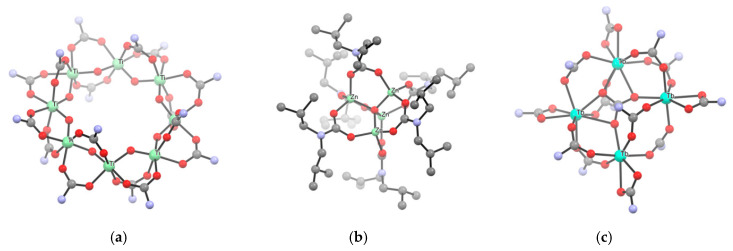
Views of X-ray crystal structures of mixed oxido-carbamato complexes obtained by controlled hydrolysis: (**a**) [Ti_8_O_8_(O_2_CNEt_2_)_16_]·[NH_2_Et_2_]·[O_2_CNEt_2_] (Et groups, ammonium cation and *N*,*N*-diethylcarbamate anion omitted for clarity); (**b**) [Zn_4_(μ_4_-O)(O_2_CN^i^Bu_2_)_6_] (H atoms omitted for clarity); (**c**) [NH_2_Bu_2_]_2_[Tb_4_(CO_3_)(O_2_CNBu_2_)_12_] (Bu groups and ammonium cation omitted for clarity). Drawings based on published data [151,283,292].

As a representative example, the octa-titanium complex [Ti(µ-O)(O_2_CNEt_2_)_2_]_8_ [NH_2_Et_2_][O_2_CNEt_2_] was generated by hydrolysis of [Ti(O_2_CNEt_2_)_4_] in 1,2-dimethoxyethane using a metal/water ratio of 1 [292]. The structure of the complex shows a chain of almost co-planar titanium atoms encapsulating a diethylammonium cation, while the carbamato anions are located outside the cycle (Scheme 33a). Instead, [Nb(O_2_CNEt_2_)_5_] treated with circa 1 eq. of water in toluene afforded a product of presumable formula [Nb_2_O_3_(O_2_CNEt_2_)_4_], being reminiscent of the structurally characterized [Ta_2_(µ-O)_3_(O_2_CNEt_2_)_4_]_4_ based on IR and NMR spectra [171].

Controlled hydrolysis of [M(O_2_CNR_2_)_2_]_n_ (M/H_2_O ratio 4:1) and [ZnR’(O_2_CNR_2_)]_4_ in toluene or THF produced the tetranuclear oxido-carbamate [Zn_4_(μ_4_-O)(O_2_CNR_2_)_6_] (R = Me, Et, *^i^*Pr, *^i^*Bu; Scheme 33b) and octanuclear [M_4_(μ_4_-O)(O_2_CN*^i^*Pr_2_)_6_]_2_ (M = Fe, Zn) [143,164,202,203,283]. In the case of [Zn_4_(μ_4_-O)(O_2_CNMe_2_)_6_], the reaction can be reversed by protonating the oxido ligand with [Me_2_NH_2_][O_2_NMe_2_], which is rather uncommon [143]. A related tetranuclear cage structure is displayed by [La_4_(µ_4_-O)(O_2_CN^i^Pr_2_)_10_], being the first lanthanide µ-oxido carbamato complex obtained by controlled hydrolysis of the corresponding homoleptic derivative [146]. The bismuth species [Bi_8_O_6_(O_2_CN*^i^*Pr_2_)_12_] was serendipitously crystallized from a solution of the homoleptic [Bi(O_2_CN*^i^*Pr_2_)_3_] upon prolonged air exposure [135].

In some cases, the interaction of metal carbamates with dioxygen is an equivalent to the hydrolytic treatment, except for the additional oxidation of the metal centers. For instance, the reaction of [Ce(O_2_CN^i^Pr_2_)_3_]_4_ with dioxygen proceeded with Ce(III) to Ce(IV) oxidation and moderate structure rearrangement to give the µ-oxido derivative [Ce_4_(µ_3_-O)_2_(O_2_CN^i^Pr_2_)_12_]. The same reaction was also performed in the solid state [144]. Oxygenation of the homoleptic Fe(II) carbamate or controlled hydrolysis of the homoleptic Fe(III) carbamate led to the μ-oxido Fe(III) carbamate [Fe_2_(μ-O)(O_2_CNR_2_)_4_] (R = Et, ^i^Pr) [143]. Conversely, [Mn(O_2_CN^i^Pr_2_)_2_]_n_ is air stable in the solid state, although its complete conversion to the Mn(III) derivative [Mn_4_O_3_(O_2_CN^i^Pr_2_)_6_] is viable in the presence of water; this result suggests that the preliminary formation of μ-oxido moieties might trigger the subsequent oxidation of the metal center.

Hydrolysis of rare earth carbamato complexes often results in the formation of a carbonate (Scheme 32c,d) [293]. Indeed, the extraction method described above related to homoleptic lanthanide complexes (Section 3.1) proceeds to the formation of mixed carbonato-carbamato species, [NH_2_Bu_2_]_2_[Ln_4_(CO_3_)(O_2_CNBu_2_)_12_] (Ln = Tb, Sm, Eu, Tm) (Scheme 33c), in case the separated organic phase is not promptly dried. This is probably ascribable to the quite fast reaction of carbamato ligands with residual traces of water [151,153]. Compounds [NH_2_Bu_2_]_2_[Ln_4_(CO_3_)(O_2_CNBu_2_)_12_] can be further converted into [Ln_4_(CO_3_)(O_2_CNBu_2_)_10_] (Ln = La, Sm) upon evaporation under vacuum [146,151]. Complexes [Ln_2_(CO_3_)(O_2_CN^i^Pr_2_)_4_] (Ln = Nd, Eu, Gd) were obtained by controlled hydrolysis (M/H_2_O ratio = 2) of the corresponding homoleptic metal carbamates [146]. Accordingly, the exhaustive hydrolysis of some lanthanide carbamates (Ln = Ce, Nd, Eu, Gd, Tb) and [Y(O_2_CNBu_2_)_4_] led to the carbonates M_2_(CO_3_)_3_ and not the oxides [146,152,154].

The reactivity of metal carbamato complexes with other protic species (HX) can be used to install different ligands (X^−^) on the metal center. These include alcohols, β-diketonates and hydrogen halides, providing access to metal alkoxides, diketonates and halometallates (Scheme 31) [42]. For instance, reactions of the tin alkylcarbamates [SnR′_n_(O_2_CNR_2_)_(4-n)_] in neat alcohol (R″OH) at high temperature produces the corresponding alkoxystannanes [SnR′_n_(OR″)_(4-n)_] in moderate to high yields [294]. Homoleptic lanthanide carbamates were recently reported to react with pentafluorophenol in the presence of 1,10-phenanthroline to form [Ln(OC_6_F_5_)_3_(phen)_3_] (Ln = Nd, Tb) [295]. A further example is given by the β-diketonato complex [Tb(dbm)_3_], which was obtained upon reaction of [Tb(O_2_CNBu_2_)_3_] with dibenzoylmethane (Hdbm) [153]. A new entry into this category is represented by terminal alkynes: [Sn(O_2_CNEt_2_)]_4_ reacts with phenylacetylene in refluxing toluene, to afford the homoleptic Sn(IV) alkynyl derivative [Sn(CCPh)_4_] [296].

Silanol groups (≡Si−OH) on the surface of silica and other similar oxides also offer a reactive site for metal carbamates, leading to the derivatization of such materials with metal carbamato fragments (see Section 5 for details).

Reactivity with nucleophiles. In contrast to the reactivity of carbon dioxide, the CO_2_ moiety within a carbamato ligand is not susceptible to nucleophilic attack and this feature enables specific modifications to the coordinative sphere of metal carbamato complexes. For instance, carbonylation [42], hydrogenation [231] and ligand exchange reactions using methyl lithium or pyridines [278,297] have been performed without affecting the integrity of the carbamato ligand(s).

The reactions of the tetrameric Zn alkylcarbamates [ZnR′(O_2_CNR_2_)]_4_ (R = *^i^*Pr, *^i^*Bu; R = Me, Et) with various *N*-donors provide a striking example of the versatility of the coordinated carbamato fragment. In fact, pyridine addition gives the dinuclear [ZnMe(O_2_CNR_2_)(py)]_2_ with switching of the carbamato coordination from triply (***B/3***) to doubly bridging (***B/2***) [201]. Differently, addition of diamines or guanidines gives the mononuclear complexes [Zn(N)(N)(O_2_CNR_2_)_2_], featured by chelating (***C/1***) or monodentate (***M/1***) carbamates, according to the electronic properties of the *N*-ligand [298,299].

### 3.4. Crystallographic and Spectroscopic Features of Carbamato Ligands

**Scheme 34:**
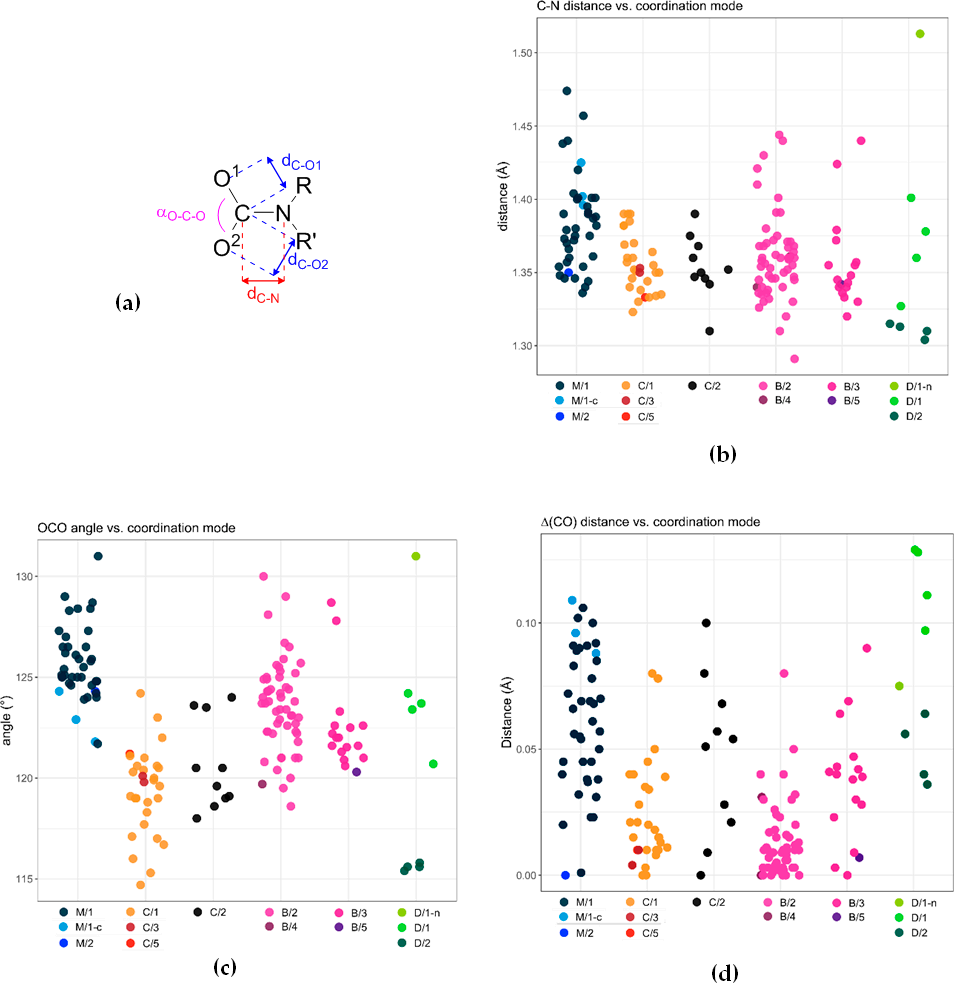
Selected structural parameters for carbamato ligands (**a**) and their distribution according to the coordination mode (**b**–**d**). Data taken from Table S3 (ESI). See Scheme 13 for the ***M***/***C***/***B***/***D*** nomenclature adopted.

A collection of crystallographic data for metal carbamato complexes published from 2004 to 2020 is reported in Table S3 (ESI). We selected C−O and C−N distances, as well as the O−C−O angle (Scheme 34a), as diagnostic structural parameters to be discussed with respect to the different coordination modes (see Scheme 13 for the ***M***/***C***/***B***/***D*** nomenclature adopted). Clearly, this analysis does not include the electronic/geometric effects exerted by different metal centers as well as the nature of substituents on the nitrogen atom.

The majority of carbon-nitrogen bond distances within carbamato ligands are distributed within the range 1.33–1.41 Å (Scheme 34b), suggestive of a substantial delocalization of the nitrogen lone pair on the CO_2_ moiety. Remarkably, such N → CO_2_ interaction is not significantly influenced by the denticity of the carbamato ligand (compare ***B/2***, ***B/3***, ***B/5*** and ***C/1***, ***C/2***, ***C/3***, ***C/5***).

Conversely, the C−O distances and the O−C−O angle are affected by the ligand coordination mode. As expected, the CO_2_ angle is smaller in chelating carbamates (‘***C***’ series in Scheme 13; 120 ± 2°), compared to the other bonding situations (123−126 ° range) (Scheme 34c). The two C–O bond lengths are quite different in monodentate carbamato ligands (‘***M***’ series in Scheme 13), averaging 1.29 ± 0.02 Å and 1.23 ± 0.02 Å; this feature reflects a prevailing double bond character of the C–O bond not involved in coordination. The difference between the two types of C–O distances (∆d_C-O_, Scheme 34d) is reduced to a few pm for chelating (***C/1***) and, particularly, bidentate bridging ligands (***B/2***; ∆d_C-O_ = 0.01 ± 0.01 Å). Binding to an additional metal center breaks the symmetry of the system and ∆d_C-O_ increases (compare ***C/1*** with ***C/2*** and ***B/2*** with ***B/3***).

Dianionic carbamyldiide ligands (***D/1*** and ***D/2*** modes in Scheme 13) possess crystallographic features that are markedly different from those of ordinary carbamato ligands. For instance, in the *N*,*O*-chelating mode (***D/1***), the two C−O bond lengths (1.32–1.37 Å and 1.22–1.24 Å) reveal a net double and single bond character, whereas a rather small O–C–O angle (≈116°) is observed when both oxygen atoms are involved in coordination (***D/2***).

From a spectroscopic point of view, diagnostic features of carbamato ligands are the ^13^C-NMR resonance and the IR absorptions related to the NCO_2_ moiety. A collection of solution NMR and solid-state IR data for metal carbamato complexes, along with the coordination mode(s) of the carbamato ligand in the solid state, can be found in Table S4 (ESI). Trends emerging from the analysis of structurally-characterized compounds, with due caution, can be a guidance for the characterization of further compounds.

**Scheme 35:**
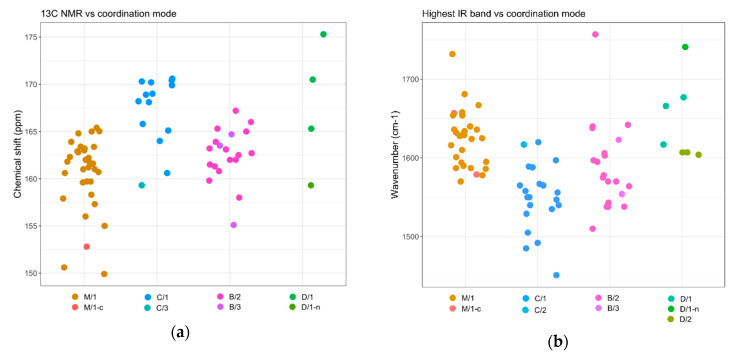
Selected spectroscopic data for metal carbamato complexes and their distribution according to the coordination mode: ^13^C-NMR chemical shift of the carbamyl carbon (**a**) and highest IR band ascribable to the NCO_2_ moiety (**b**). Data refer to Table S4 (ESI).

The ^13^C-NMR chemical shift of the carbamyl carbon in metal carbamato complexes is typically around 160 ppm. In cases where a single coordination mode was determined, signals ascribable to monodentate carbamates (***M/1*** mode) were reported in the 156−164 ppm range. Signals belonging to chelating (***C/1***) and bridging (***B/2***) carbamato ligands fall in the upper half of this interval and even beyond (up to 170 ppm) (Scheme 35a). However, it has to be considered that the number and the position of the ^13^C-NMR resonances in solution systems might not be discriminating where multiple coordination modes are adopted. Indeed, quite often only an average value is observed at ambient temperatures, due the rapid exchange between carbamato ligands in solution [210]. In such cases, ^13^C-NMR measurements at low temperatures provide distinct chemical shifts for the carbamates in the different coordination fashions [138,284]. The few spectroscopic data available for carbamyldiide ligands, in the chelating *N*,*O* mode (***D/1***), show a downfield-shifted ^13^C-NMR signal (165–175 ppm).

Metal carbamato complexes generally show multiple medium/strong IR bands in the region 1300−1700 cm^−1^, which are ascribable to stretching vibrations of the NCO_2_ moiety [42]. The highest-frequency IR band has been typically assigned to the C=O stretching/CO_2_ antisymmetric stretching, in analogy to metal-carboxylates [300,301]. However, the involvement of the N atom in the π-system makes the signal assignment far less clear-cut than in metal carboxylates [242]. As previously pointed out [42], the position of the IR bands may give some indications on the coordination mode of the carbamate. In this regard, the wavenumber of the highest IR absorption versus the coordination mode, for compounds having a univocal association between the two, can be visualized in Scheme 35b.

Monodentate ligands (***M/1****)* are featured by an intense absorption generally ranging from 1600 to 1660 cm^−1^, and occasionally higher. Conversely, lack of a band above 1600 cm^−1^ has been interpreted in terms of an absence of terminal ligands, although in some cases hydrogen bonding involving the uncoordinated oxygen atom may reduce the wavenumber below 1600 cm^−1^ [42]. On the other hand, chelating (***C/1***) and bidentate (***B/1***) ligands display their highest-frequency band in the 1535–1600 cm^−1^ range, associated to a second strong band around 1490–1530 cm^−1^. However, the positions of these absorptions do not allow a clear distinction between the two coordination modes [42].

## 4. Catalysis with Metal Carbamates

Despite that carbamato complexes have been known for more than 50 years [122] and their reactivity has been widely studied during this time, a systematic investigation on their catalytic behavior began only recently. As a matter of fact, homoleptic carbamates and related systems possess a number of properties that make them attractive candidates for applications in catalysis: in particular, they are easily available from relatively cost effective and nontoxic chemicals all across the periodic table, and exhibit a considerable structural diversity (Section 3). Basically, two aspects mentioned above constitute the key to the interest in the potential use of metal carbamato complexes as catalysts. First, the formation of the carbamato unit is a way to fix CO_2_, which is also exploited in nature with reference to some Ni(II) [302] and Zn(II) [303,304,305] enzymes. Trapped CO_2_ can be used as a C1 synthon in organic synthesis, and the stoichiometric reactions of metal carbamates with organic electrophiles can lead to CO_2_ incorporating products (Section 3.4). This result can be achieved even using the metal species in a catalytic amount, with the carbamato ligand(s) playing an active role in the process. The second aspect has a broader significance, and is related to the possible generation of a vacant site on the metal center, due to both the flexibility of the carbamato ligand adapting from bi- to monodentate coordination, and to its ability to behave as a leaving group, following the interaction with proton-active substances.

In this section, the relevance of carbamato complexes as catalytic precursors and/or intermediates in organic reactions will be discussed, starting from CO_2_ activation reactions. The proposed mechanisms will be outlined, in order to highlight the presumable role of the carbamato ligand in the catalytic cycle.

### 4.1. CO_2_ Activation Routes

The formation of carbamato complexes is considered a key step in metal-mediated reactions such as the CO_2_/aziridine coupling [306,307,308] and the CO_2_ cycloaddition to propargyl amines [113,309,310,311,312] or aminoalcohols [306]. The production of oxazolidinones (cyclic carbamates) via CO_2_/aziridine coupling is one of the most widely investigated carbon dioxide fixation processes, and many species have been evaluated as catalytic precursors, such as Al(III) [313], Cr(III) [314] and Co(III) [315] salen complexes, and Cu(II) [316] and Zn(II) [317] porphyrin complexes. Regarding the cycloadditon of CO_2_ to propargylamines, catalysts based on late transition metals are privileged since they offer the possibility to activate the alkyne reactant via η^2^-coordination [113,318]. The synthesis of cyclic carbamates starting from amino-alcohols and CO_2_ is a less explored route if compared with the other ones, and a limited number of catalysts have been studied in this regard, based on cesium, silicon and tin [319,320,321].

**Scheme 36:**
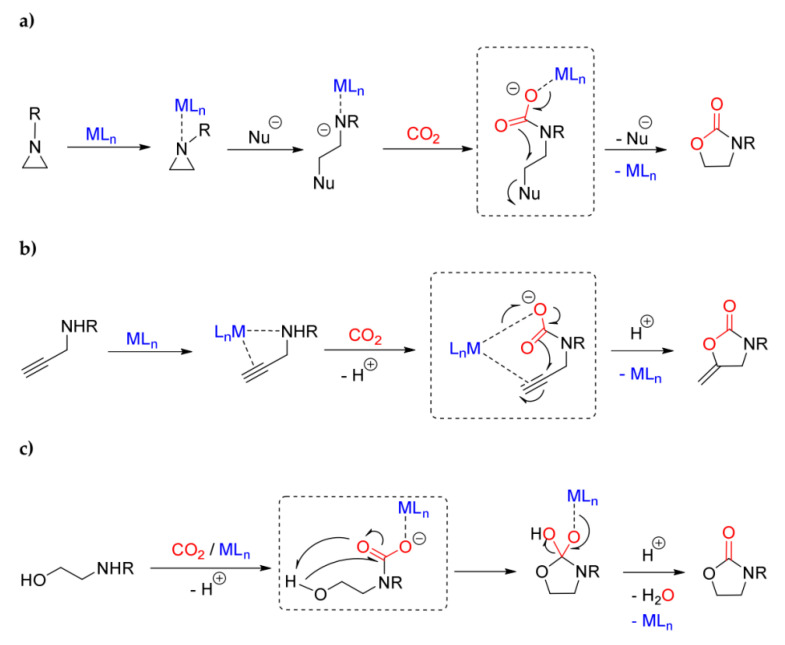
Generally-accepted mechanisms for metal-mediated aziridine/CO_2_ coupling (**a**), CO_2_ cycloaddition to propargylamines (**b**) and aminoalcohols (a 2-aminoalcohol is drawn as example) (**c**). The intermediate carbamate in each pathway is circled.

The generally accepted mechanisms for these reactions are outlined in Scheme 36a–c. In all cases, the intermediate generation of a carbamato ligand is postulated upon interaction of the *N*-donor and CO_2_ with the metal center. Afterwards, electrophilic attack on the oxygen atoms (Scheme 36a,b), combined with a nucleophilic attack on the carbon atom (Scheme 36c), generates the product.

In addition to the above mentioned reactions, Jiang et al. reported the involvement of Cu(II) and Cu(III) carbamates in the oxidative coupling of arylboronic acids [322] and in the cyclization of enynes [323] from amines and CO_2_. In order to support the hypothesis of an in situ formed Cu(II) carbamate, the catalytic activity of [Cu(O_2_CNBn)_2_(NHBn_2_)_2_] was evaluated, providing moderate to good results in term of product yields.

**Scheme 37:**
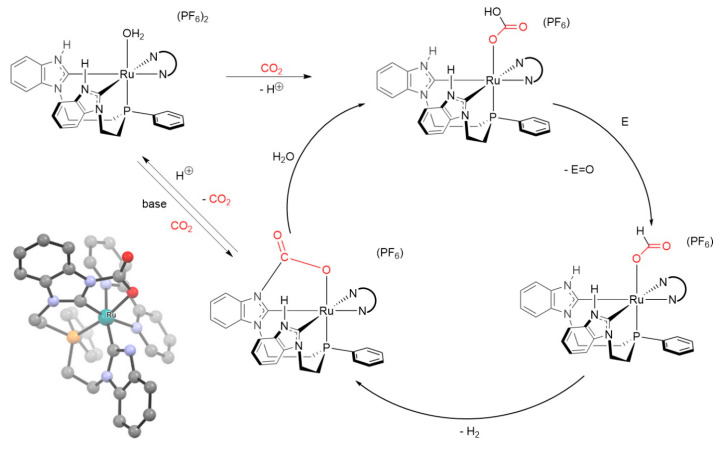
H_2_O to H_2_ conversion catalyzed by a Ru(II) complex with the assistance of CO_2_ and view of the X-ray crystal structure of the supposed metal carbamato intermediate. Drawings based on published data (H atoms omitted for clarity) [270].

An interesting case of CO_2_ activation was reported in 2016 by Norris et al. The authors described the role of carbon dioxide in the evolution of H_2_ from water using a Ru(II) complex. In this particular system, CO_2_ works as a co-catalyst in association with the ruthenium complex, generating a carbamate as a reaction intermediate (Scheme 37) [270].

**Scheme 38:**
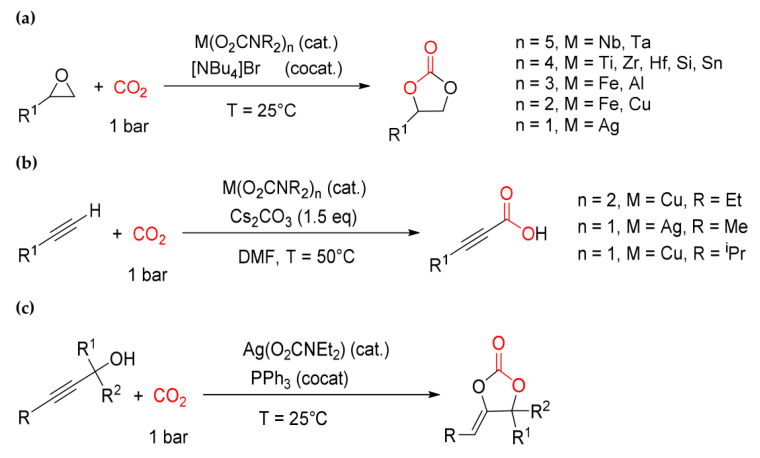
Cycloaddition of CO_2_ to epoxides (**a**), carboxylation of terminal alkynes (**b**) and cycloaddition of CO_2_ to propargyl alcohols (**c**) mediated by homoleptic metal carbamates.

Although the formation of a metal carbamate represents a generally accepted step in many carbon dioxide activation reactions, the direct use of carbamato complexes as catalytic precursors is a recent approach. Thus, a systematic screening of the catalytic activity of homoleptic carbamates of silicon, tin and some d-transition metals for the CO_2_/epoxide coupling reaction was performed, in conjunction with tetrabutylammonium bromide as a co-catalyst, under solvent-free and ambient conditions [134,290,324]. Then, Ag(I), Cu(I) and Cu(II) carbamates were tested as catalysts for the carboxylation of terminal alkynes [325] and in the cycloaddition reaction of CO_2_ to propargyl alcohols [326] (Scheme 38).

**Scheme 39:**
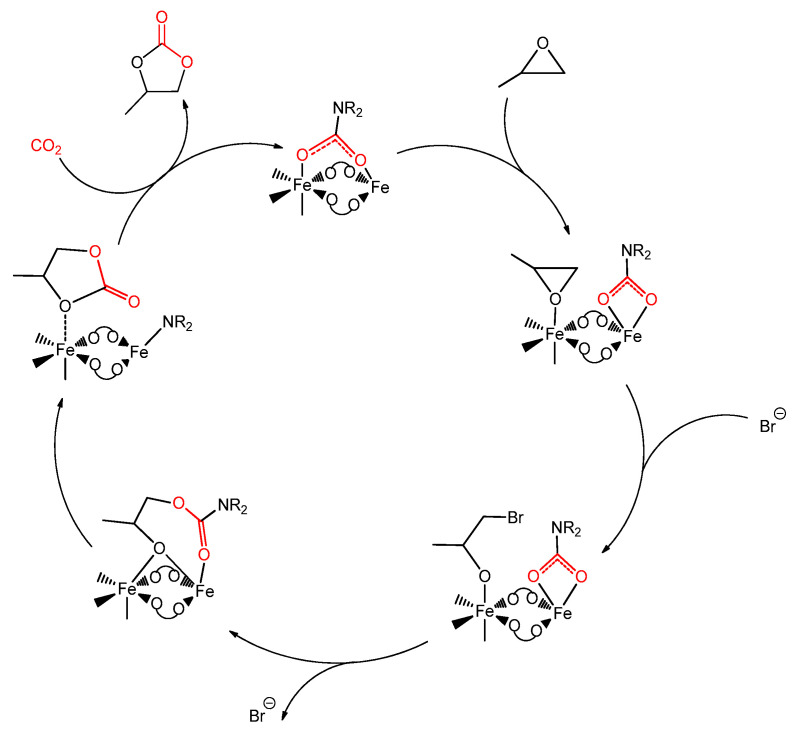
Proposed mechanism for the epoxide/CO_2_ coupling via dynamic trapping of carbon dioxide into an iron(III) carbamate. The remaining carbamato ligands in the structure are omitted for clarity.

Interestingly, [Fe(O_2_CNR_2_)_3_] (R = Et, ^i^Pr, Bn) revealed a promising catalytic activity for the production of cyclic carbonates from CO_2_ and epoxides [324]. It is remarkable that an inexpensive catalyst, based on a nontoxic metal element and working at ambient temperature and CO_2_ pressure, is an appealing requisite in terms of sustainability [327]. The catalytic mechanism was elucidated by NMR and DFT analyses, suggesting the occurrence of an unusual dynamic CO_2_ pre-activation, possibly responsible for the activity of the complex in mild conditions (Scheme 39). The CO_2_ pre-activation occurs through the preliminary incorporation of the CO_2_ reactant in a carbamato ligand, followed by transfer to the organic substrate and ready restoring of the carbamato unit guaranteed by external CO_2_. A similar pathway was recently proposed by Bayer et al., studying the CO_2_/epoxide coupling by means of dimethylpirazolate cerium amides and cerium carbamates [134].

In summary, metal carbamates are easy-to-synthesize complexes and their ligands can adapt their coordination mode or can be protonated to form a vacant site on the metal center. Moreover, carbamato ligands themselves represent a pre-activation form of carbon dioxide and can exchange the CO_2_ fragment within the ligand with external carbon dioxide (see Section 3.3), suggesting some potential in the dynamic activation of this molecule. All these characteristics, combined with the possibility of employing a nontoxic metal center, delineate metal carbamates as potential catalytic systems useful in CO_2_ activation reactions and deserving of further studies and progress.

### 4.2. Other Catalytic Processes

Polymerization. The first study concerning the catalytic activity of metal carbamates in a polymerization reaction was reported in 2009 [328]. More precisely, [Nb(O_2_CNR_2_)_5_] (R = Me, Et) were employed in the ring opening metathesis polymerization (ROMP) of norbornene in the presence of methylaluminoxane (MAO). Interestingly, such niobium catalysts are very active in chlorobenzene and especially [Nb(O_2_CNEt_2_)_5_] was tagged as the most active niobium catalyst ever reported for norbornene-ROMP. The increased steric hindrance around the metal center in the ethyl derivative is believed to favor α-hydrogen elimination and thus to accelerate the reaction.

Subsequently, [TiCl_2_(O_2_CNMe_2_)_2_] [139], [Ti(O_2_CNR_2_)_4_] (R = Me, Et, Pyrr) [139,172,329], [Nb(O_2_CNR_2_)_5_] (R = Me, Et), [Nb(O_2_CNEt_2_)_4_] and [Nb(O_2_CNEt_2_)_3_] [280] were studied in ethylene and propylene (homo)polymerization and ethylene/1-hexene copolymerization. Notably, the catalytic activities of these carbamato complexes were higher compared to those of the respective metal halide precursors. Concerning the ligand framework, the steric hindrance of the alkyl group enhances the catalytic performance in ethylene polymerization (Et > Me), presumably by inhibiting the formation of inactive polymetallic species. On the other hand, in propylene polymerization, an increase of steric hindrance around the metal center results in a drop of catalytic activity, imputable to the easier attack of the incoming monomer when the *N*-alkyl group is small [139].

**Scheme 40:**
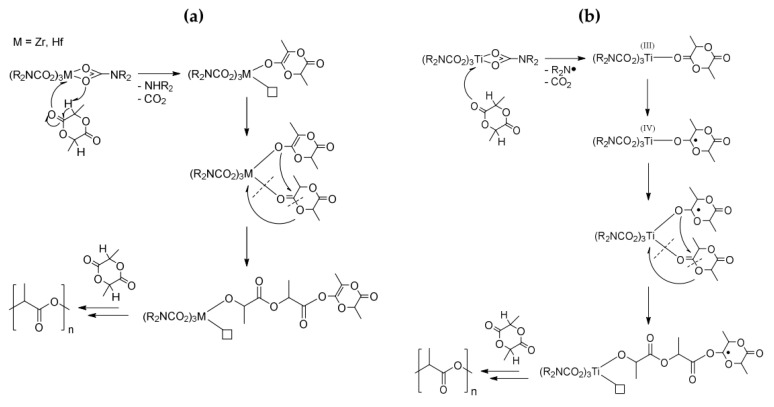
Proposed mechanisms for the *rac*-lactide polymerization promoted by homoleptic tetracarbamates of Zr, Hf (**a**) or Ti (**b**).

Group 4 metal tetrakis-carbamato complexes, i.e., [M(O_2_CNR_2_)_4_] (M = Ti, Zr, Hf; R = Et, ^i^Pr), were also studied as catalysts in the ring opening polymerization (ROP) of *rac*-lactide [330]. As already observed for other catalytic processes, the titanium compounds showed a lower activity compared to that of zirconium and hafnium and the best results were obtained with the most sterically hindered R group. The polymerization mechanism was enlightened by IR and NMR studies, revealing two different pathways depending on the catalyst type. For zirconium and hafnium derivatives, *rac*-lactide coordination to the metal center occurs following α-hydrogen elimination promoted by the basic character of the carbamato ligand (Scheme 40a). Conversely, in the case of Ti(IV), a radical polymerization mechanism is operative, triggered by an initial electron interchange between the carbamato ligand and the *rac*-lactide molecule (Scheme 40b).

Other catalytic processes. To the best of our knowledge, the first investigation on the catalytic activity of a metal carbamate was reported by Belli Dell’Amico et al. in 2004 [231]. Thus, Ru(II) carbamates of formula [Ru(O_2_CN*^i^*Pr_2_)_2_(PPh_3_)_2_] and [RuCl(O_2_CN*^i^*Pr_2_)(PPh_3_)_3_] were tested in the 1-octene H_2_ hydrogenation at atmospheric pressure, in toluene at ambient temperature. The catalysts were recovered unchanged at the end of the process, suggesting that the required alkene coordination during the catalytic cycle is ensured by a simple coordination switch of the carbamato unit(s) from chelating to monodentate.

In other cases, transition metal catalysts have been reported to work via intermediate formation of carbamato ligands [331,332]. For instance, the conversion of cyclobutanes to Z-enol carbamates catalyzed by Cp_2_Zr(CH_2_=CH_2_) was postulated to pass through a Zr(IV) carbamate [331]; additionally, a potassium carbamate was detected as an intermediate in the catalyzed Lossen rearrangement of hydroxamic acids to isocyanate [332].

## 5. Other Applications

Metal carbamato complexes have been investigated in several research areas beside catalysis, especially during the last decade. In material chemistry, the viable degradation of easily-accessible d/f metal homoleptic carbamato complexes has been exploited to obtain nanostructured metal oxides, whereas silver carbamato complexes have been used as precursors to nanomaterials. In addition, both homoleptic and heteroleptic carbamates have been employed to functionalize the shell of silica and other oxides, taking advantage of the controlled reactivity with surface hydroxyl groups. These and other aspects will be detailed in the following, focusing on the reactivity of the carbamato moiety and the properties of the complexes relevant to each application.

**Scheme 41:**
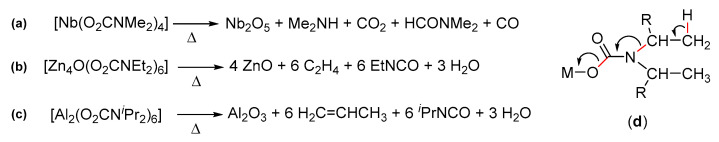
Products detected by pyrolysis of [Nb(O_2_CNEt_2_)_4_] (**a**); thermal decomposition reactions of [Zn_4_O(O_2_CNEt_2_)_6_] (**b**) and [Al_2_(O_2_CN*^i^*Pr_2_)_6_] (**c**) and proposed fragmentation mechanism (**d**).

Metal carbamates as precursors to nanostructured metal oxides. Over the last 15 years, carbamato complexes have been widely investigated for the preparation of nanostructured metal oxides, arousing interest for their electric, magnetic, optical and catalytic properties. The formation of a metal oxide from a metal carbamate basically takes place via either thermal degradation or exhaustive hydrolysis. The thermal degradation of homoleptic carbamates or oxido-carbamates under inert atmosphere usually proceeds quantitatively at temperatures below 500 °C with fragmentation of the organic groups, cleanly affording a metal oxide. The fate of the carbamato ligands during the pyrolytic process depends on the system; in general, multiple products have been detected in the gas phase, including CO_2_ and the dialkylamine [42,240,333,334] (Scheme 41a). In some cases, the preferential formation of alkyl isocyanates and alkenes has been recognized (Scheme 41b–d) [335,336].

Homoleptic *N*,*N*-dialkylcarbamato complexes, or oxido-carbamates, can be easily sublimed under reduced pressure, a fact that can be justified on the basis of their molecular structure and lack of strong intermolecular interactions (e.g., H-bonds) in the solid state [334]. Therefore, such compounds are ideal precursors for the Chemical Vapor Deposition (CVD) technique. Moreover, the use of a single component as oxide precursor (“single source CVD,” or SSCVD) is advantageous with respect to classical CVD methods requiring at least two reagents for the gas-phase reaction.

According to the SSCVD methodology, the metal *N*,*N*-dialkylcarbamate is volatilized at 150–200 °C under high vacuum (1–5 × 10^−6^ torr), or under an N_2_ flow, and then it is thermally decomposed on a silicon wafer (or another substrate material) around 500 °C. The gap between the two temperatures provides a suitable working window. Hence, thin films of ZnO [336,337,338,339], Al_2_O_3_ [335] and Bi_2_O_3_ [135] have been prepared from [Zn_4_O(O_2_CNEt_2_)_6_], [Al_6_(O_2_CN*^i^*Pr_2_)_12_] or [Bi(O_2_CN*^i^*Pr)_3_]. Similarly, heterobimetallic Zn/Mg carbamates of general formula [Zn_x_Mg_4−x_O(O_2_CN*^i^*Pr_2_)_6_] were useful to prepare Zn_x_Mg_(1−x)_O thin films [340,341]. The use of a bimetallic precursor is convenient respect to the co-deposition from two distinct compounds, because the sublimation occurs at a defined temperature, leading to a ratio between the two metals in the mixed oxide that is given by the precursor composition. Metal oxide films obtained through SSCVD can be as low as 200 nm thick and present a smooth surface, low carbon contamination, high density and a single preferred crystallite orientation.

The same principles can be applied to the preparation of metal oxide nanoparticles by thermal decomposition of metal carbamates at 200–300 °C and ordinary pressure. Thus, pyrolysis of [ZnEt(O_2_CNR_2_)]_4_ and [M(O_2_CNR_2_)_4_] (M = Zr, Hf, Nb; R = Me, Et, *^i^*Pr) gives ZnO, ZrO_2_, HfO_2_ and Nb_2_O_5_ nanoparticles [203,334], whereas Zn/Co and Zn/Mn heterobimetallic carbamates have been employed to obtain Zn_x_(Co, Mn)_(1−x)_O mixed oxide nanoparticles [281,282]. In these cases, the excessive volatilization of the metal carbamate is a possible disadvantage [334].

**Scheme 42:**
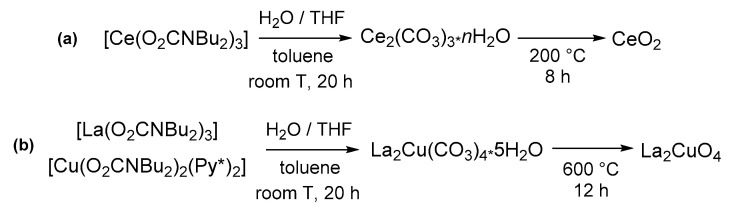
General protocol for the preparation of oxides from metal carbamates applied to the preparation of CeO_2_ (**a**) and mixed La/Cu oxide (**b**) nanoparticles. Py* = substituted pyridine.

Operating in the condensed phase, the transformation of a metal carbamate into its oxide can be realized by thermal degradation but also by reaction with protic species (Section 3.3). For instance, thin films of Al_2_O_3_ were deposited from solvothermal decomposition of [Al_6_(O_2_CN*^i^*Pr_2_)_12_] in dry benzene [342]. Additionally, hybrid procedures are known, involving a thermally-assisted degradation in the presence of protic species such as alcohols, oleic acid or hydrogen peroxide in organic solvents, to afford ZnO or CuO nanoparticles [158,343,344]. Among the reactions with protic species, the hydrolytic route is the privileged one and has been optimized to access various oxide nanoparticles. In order to control the reaction and to obtain a finely dispersed powder, a water/THF mixture is conveniently added to a solution of the metal carbamate in toluene or heptane under inert atmosphere. For compounds based on Y, Ce and other lanthanides, exhaustive hydrolysis produces the metal carbonate, which can be subsequently calcined to give the oxide (Scheme 42a) [152,154]. When the hydrolysis is carried out in the presence of additional metal precursor(s) (as a carbamate, or a hydrolysable species in general), a mixed carbonate is obtained with a composition regulated by the relative amounts of the reactants. Upon further thermal treatment, mixed metal oxide nanoparticles with a desired composition, such as Ce_1.65_Tb_0.35_O_3.82_, Ce_1.80_La_0.20_O_3.90_, La_2_CuO_4_, Y_3_Al_5_O_12_ and Y_2.98_Nd_0.02_Al_5_O_12_, are finally produced (Scheme 42b) [146,154,297].

**Scheme 43:**
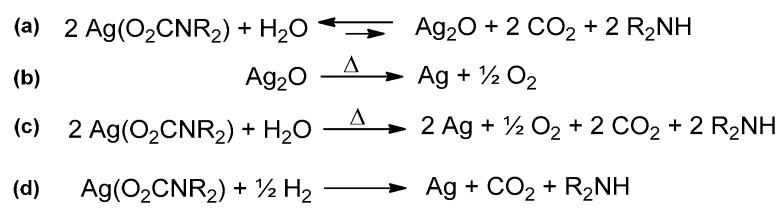
Hydrolysis (forward reaction) or preparation (backward reaction) of a silver carbamate (**a**); thermal decomposition of silver oxide (**b**); hydrolytic thermolysis (**c**) or reduction (**d**) of a silver carbamate.

Silver carbamates as precursors to silver metal nanoparticles. Silver carbamato complexes possess a peculiar chemistry. In fact, owing to the relatively low stability of Ag_2_O (∆G°_f_ = −11.2 kJ/mol at 25 °C), this is essentially the only metal oxide that can be used as precursor to homoleptic carbamates (see Section 3.1) (Scheme 43a). On the other hand, Ag_2_O is thermally unstable upon mild heating (Scheme 43b), and therefore, the hydrolytic thermolysis of silver carbamates generates metallic silver (Scheme 43c). Likewise, elemental silver is recovered from the reactions of silver carbamates with mild reducing agents (Scheme 43d) [159]. The deposition of silver is usually fast and quantitative, accompanied by the formation of volatile side products.

On account of these considerations, commercial alcoholic solutions of silver *N*-alkylcarbamates are used to access silver metal nanoparticles (AgNP). The decomposition is most commonly carried out by hydrolytic thermolysis with conventional or microwave heating or, in some cases, by using H_2_ or hydrazine as a reducing agent. The generated silver nanoparticles can be adsorbed on various matrices such as polymers (PVA, PMMA, PET), graphene, thiol-modified carbon nanotubes or fabrics (cotton, silk). The resulting silver-coated materials have been investigated for their conductive properties [345,346,347,348] and/or antibacterial/antimicrobial activity [349,350,351,352,353,354,355]. In addition, if the reduction is performed in the presence of ethyl cellulose, a silver paste is obtained, and this can be deposited on a suitable substrate and sintered to provide a silver conductive film [356,357,358].

The synthesis of silver nanoparticles (and related nanocomposites) by thermolysis of silver carbamates is preferable respect to more traditional protocols (i.e., reduction of AgNO_3_ with NaBH_4_ or sodium citrate), in that it does not require any reducing agent and thus avoids the presence of undesired inorganic species in solution. Moreover, the colloidal AgNP dispersion is stabilized by the ammonium carbamate co-product acting as surfactant (vide infra), and the spherical particles exhibit a rather narrow size distribution [352,357].

**Scheme 44:**
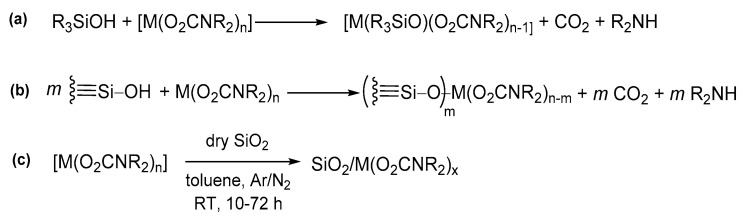
Reactions of a homoleptic metal carbamates with a model tri-organosilanol (**a**), with surface silanols groups on silica (“grafting reaction”) (**b**) and protocol for the chemical implantation on silica (**c**).

Metal carbamates as precursors for surface functionalization. Metal carbamato complexes are prone to react with a variety of protic species; these reactions are thermodynamically driven by the release of CO_2_ (Section 3.3). Alkyl and aryl silanols, although possessing a relatively mild Brönsted acidity, promptly react with metal carbamates affording the corresponding siloxide derivatives (Scheme 44a) [42,359]. This reactivity is replicated on the surface of silica due to the presence of {≡Si–OH} groups. The ensuing “grafting reaction” consists in the protonation of the carbamato moiety, with consequent release of CO_2_ and the amine, and binding of the metal atom to the silica as a siloxide unit (Scheme 44b). Despite a possible molar excess of surface silanols (vide infra), a limited number of carbamato ligands per metal complex are involved in the process, and solid-state spectroscopic measurements (IR, NMR, EPR) agree in that the final metal fragment retains its nuclearity and the geometry of the residual ligands [131,234,360,361]. In other words, the grafting reaction allows the chemical implantation of tailored metal fragments over the surface of silica.

Recently, the study of the reactivity of homoleptic *N*,*N*-dialkylcarbamates with silica has been extended to Cu(II), Nb(III), Nb(V), Ta(V), Tb(III) and Eu(III) derivatives [131,153,362,363]. Silica grafting has been also realized with non-homoleptic complexes of group 4 and 5 metals, wherein the carbamato ligands are the most reactive ones [166,234,290]. Even magnetite nanoparticles [364] and silica/zirconia [362] have been decorated with metal carbamates.

The protocol for the chemical implantation is commonly performed by suspending silica in a solution of the selected metal carbamate in toluene or heptane for a prolonged time (from several hours to days) (Scheme 44c). The reaction is conducted under inert atmosphere with anhydrous solvents and pre-dried silica, to limit the useless consumption of carbamato units by traces of water. A preliminary assessment of the silanol content of silica is useful to establish the minimum OH/metal ratio needed for a quantitative reaction, and thus, to avoid the waste of metal complex in solution.

The outcome of the grafting process is strongly dependent on the nature of the metal precursor. Indeed, multiple carbamato ligands within the same metal complex or additional basic ligands, i.e., cyclopentadienyls [234], may be involved in the reaction. The process can be featured by a variable selectivity, thus generating different metal fragments on the oxide surface. The average number of reacted carbamato ligands per metal center can be estimated by measuring the volume of CO_2_ released during the grafting process. Alternatively, a gas-volumetric titration of the final material provides the number of residual carbamato ligands.

Silica-grafted metal carbamato species have been investigated for their catalytic properties compared to the homogeneous congeners [290]. In addition, the grafted carbamato complex may undergo subsequent chemical modifications. For instance, noble metals such as Cu, Pd and Pt can be chemically or thermally reduced to give the respective silica-supported metal nanoparticles [361,362,365]. Another post-functionalization strategy entails the introduction of additional ligands by replacement of the residual carbamates, and for instance this method has been adopted to introduce chiral β-diketonates on lanthanide-grafted carbamates for chiroptical luminescence applications [153,363].

**Scheme 45:**
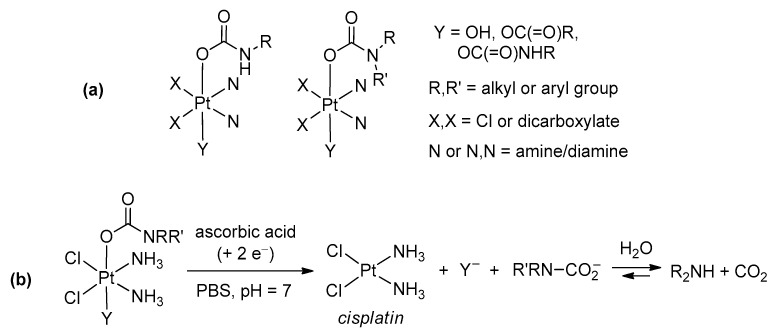
General structure of Pt(IV) carbamato complexes investigated as potential anticancer agents (**a**) and reduction to Pt(II) derivatives with release of the carbamate ion (**b**).

Platinum carbamates as anticancer agents. Carbamato complexes of Pt(IV) have been considered as potential anticancer agents (Scheme 45a), and synthetic details have been discussed in Section 3.2. Platinum(IV) carbamato complexes with simple organic substituents on the nitrogen revealed a considerable cytotoxicity against various cancer cell lines, comparable or superior to that of the reference Pt(II) drug cisplatin [260,262,263]. A C_16_ aliphatic chain (fatty acid-like) was introduced through the carbamato ligand to enhance cellular uptake [263,264,266,366,367,368]. Following a different approach, the incorporation of a maleimide fragment enabled specific interactions with proteins (e.g., addition to thiol residues) for compounds showing a promising anticancer activity in vivo [261,268,369]. The conjugation with suitable targeting groups, to enhance the pharmacological performance, can be achieved via modification of either the axial carboxylato co-ligand [367,370] or the carbamato ligand itself [272,371,372]. Remarkably, all of these Pt(IV) complexes are stable for several hours or even days in physiological aqueous solutions [260,261,263,272]. However, the reduction to Pt(II) by biological reductants triggers the release of the carbamato ligand, which is subsequently hydrolyzed to amine and CO_2_ (Scheme 45b) [272]. Thus, the synthetic design of a Pt(IV)-carbamato complex represents a strategy for a controlled and simultaneous release of a cytotoxic Pt(II) compound and (a) biologically active molecule(s) carrying an amino group into cells.

Metal carbamates for CO_2_ capture. The formation of metal carbamates from gaseous CO_2_ and a metal amine complex has direct implications in the CO_2_ capture and storage (CCS) technology [373,374,375,376,377]. In particular, amine-functionalized metal organic frameworks play an important role in the development of new solid sorbent materials [378].

**Scheme 46:**
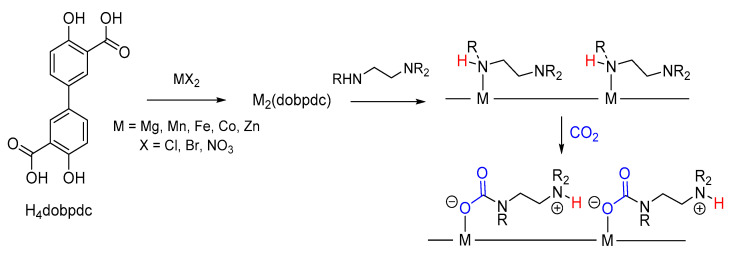
Preparation of diamine-appended MOFs and insertion of CO_2_ affording the ammonium carbamate.

The most intriguing results are related to recently-synthesized magnesium and manganese MOFs based on hydroxybenzoate ligands, and surface-derivatized with 1,2- and 1,3-diamines (Scheme 46) [376,379]. These systems reveal an extremely high affinity for CO_2_ with respect to other gases, even under atmospheric pressure (Pco_2_ = 0.39 atm), and are characterized by a peculiar step-shaped isotherm for CO_2_ uptake, allowing complete adsorption/desorption cycles in a narrow temperature range.

Contrary to the expected carbonation of the dangling amino group, spectroscopic and X-ray diffraction data agree in indicating the joint formation of a metal carbamato linkage and protonation of the diamine (“ammonium carbamate”) [376]. The use of a diamine, instead of a monoamine, provides cooperativity to the system, in that one end of the ligand undergoes reversible CO_2_ insertion while the other functions as a proton relay, stabilizing the adjacent unit via H-bonding/ion pairing. Such combination of Lewis/Brønsted acid-base reactivity resembles the one acting in amine/superbase systems (see Section 2.3). The synthesis of diamine-appended MOFs was extended to other transition metals (Fe, Co, Ni, Zn) and the related CO_2_ uptake isotherm varies according to the relative metal-amine/metal-carbamate bond strengths. Further adjustments can be accomplished by changing the *N*-substituents on the diamine [380,381], eventually supplying chirality [382].

Other applications. Some metal carbamato complexes have been investigated as additives to modify the physico-chemical properties of liquid systems. In this regard, bis-carbamates based on *N*,*N*-di(propylamino)dodecylamine produce under alkaline conditions a stable sodium bis-carbamate that is able to act as a surfactant [383,384]. This system is “switchable,” as it can be reversed by heating or bubbling N_2_. Moving to non-aqueous systems, homoleptic *N*,*N*-dialkylcarbamates of group 4 and 5 metals have been dissolved in bis(trifluoromethylsulfonyl)imide-based ionic liquids under anhydrous conditions [385]. These complexes showed a surprising solubility in such highly polar media, considering their neutral, nonpolar nature [131]. Spectroscopic measurements and DFT calculations outlined that the coordination sphere of the metal was preserved upon dissolution, paving the way to metal electrodeposition or catalytic processes.

Finally, some heterobimetallic, polynuclear Zn/Dy and Zn/Gd complexes featuring carbamato ligands have been investigated for their low-temperature magnetic properties [247,386].

## 6. Conclusions

The carbamato unit deriving from the basic combination of a non-tertiary amine with carbon dioxide can be effectively entrapped in molecular complexes of metal (or semimetal) elements across the periodic table. The synthetic procedures are generally straightforward and do not need high pressure equipment, thus allowing the easy access to both homoleptic and hybrid metal species. Several coordination modes are viable for the carbamato ligand, and the interconversion from bi- to monodentate coordination has been often observed, suggesting a versatile character. The reactivity of the metal-coordinated carbamato moiety is reversed respect to that of carbon dioxide; thus, metal carbamates are unreactive towards nucleophilic addition, but they may readily decompose upon treatment with electrophilic (protic) reagents, leading to the liberation of carbon dioxide and the amine. The peculiar and attracting features of this numerous and variegate class of metal compounds render them intriguing candidates in the perspective of various applications, and there has been a significant advance in recent years especially in the fields of catalysis and material chemistry. More specifically, the catalytic potential of metal carbamates appears promising and deserving of further and deeper developments in CO_2_-fixation organic routes; indeed, the possible dynamic exchange between the carbamato [CO_2_] fragment and external CO_2_ may constitute an unusual way for the pre-activation of carbon dioxide, accelerating the overall catalytic process. On the other hand, the facile degradation of the carbamato unit upon contact with acidic groups is a potent tool for the targeted synthesis of metal nanoparticles, films of metal oxides and for the controlled decoration of solid materials with metal units (including lanthanides and heterogeneous systems) providing special properties.

## Figures and Tables

**Table 1:** Carbamate formation rate (k) and equilibrium (K_CBM_) constants at 18 °C in water (Reaction: 2NHRR′ + CO_2_ ⇋ [NH_2_RR′][O_2_CNRR′]). Carbamate hydrolysis equilibrium constants (K_HYD_) at 18 °C in water (Reaction: NRR′CO_2_^−^ + H_2_O ⇋ HCO_3_^−^ + NHRR′). More data available in Table S1.

Amine	pK_b_^[a]^	K_CBM_	K_HYD_	Ref.
NH_3_	4.76	2.3 × 10^3^	4.4 × 10^−1^	[42]
NH_2_Me	3.38	4.0 × 10^6^	6.0 × 10^−3^	[42]
NHMe_2_	3.22	1.6 × 10^6^	2.2 × 10^−2^	[42]
NH_2_Et	3.19	2.0 × 10^6^	1.8 × 10^−2^	[42]
NHEt_2_	3.51	7.4 × 10^4^	2.4 × 10^−1^	[42]
NH_2_^i^Pr	3.37	3.6 × 10^5^	6.3 × 10^−2^	[42]
NH^i^Pr_2_	3.17	None	-	[42]
NH_2_^s^Bu	3.44	3.8 × 10^5^	4.9 × 10^−2^	[42]
NH^s^Bu_2_	-	None	-	[42]
NH_2_Ph	9.30	8.1 × 10^−3^	3.6	[42]
Piperidine	2.95	7.9 × 10^5^	8.1 × 10^−2^	[42]
3-MPD	3.12	6.9 × 10^6^	6.2 × 10^−3^	[58]
4-MPD	3.06	5.9 × 10^6^	8.3 × 10^−3^	[58]
Pyrrolidine	3.16	1.9 × 10^7^	2.0 × 10^−3^	[54,55]
MEA	4.42	6.0 × 10^4^	1.9 × 10^−2^	[42]
DEA	4.98	2.1 × 10^3^	1.5 × 10^−1^	[42]
1-AP ^[b]^	4.75	9.6 × 10^3^	1.1 × 10^−1^	[54]
2-AP ^[b]^	4.75	4.0 × 10^3^	2.5 × 10^−1^	[52]
MPA ^[b]^	4.18	2.5 × 10^5^	1.5 × 10^−2^	[52]
AMP ^[b]^	4.73	None	-	[52]
4-PIPDM ^[b]^	3.71	2.7 × 10^5^	4.1 × 10^−2^	[54,55]
4-PIPDE ^[b]^	3.65	3.0 × 10^5^	4.2 × 10^−2^	[55]
Morpholine ^[b]^	5.78	1.4 × 10^3^	6.8 × 10^−2^	[54,55]
Thiomorpholine ^[b]^	5.57	9.3 × 10^2^	1.6 × 10^−1^	[54,55]
Piperazine	4.50	5.5 × 10^4^	3.2 × 10^−2^	[53]
	8.67	2.6 × 10^−1^	4.6 × 10^−1^	
MPIPZ	4.96	5.1 × 10^3^	1.2 × 10^−1^	[54]
4-AMTHP ^[b]^	4.37	1.9 × 10^5^	1.3 × 10^−2^	[56]
Taurine ^[b],[c]^	5.19	7.1 × 10^3^	5.1 × 10^−2^	[57]
*Amino acids*				
Glycine^[d]^	4.49	4.4 × 10^4^	4.2 × 10^−2^	[59]
Sarcosine ^[b],[d]^	4.22	3.3 × 10^4^	1.0 × 10^−1^	[60]
α-Alanine ^[d]^	4.40	1.8 × 10^4^	1.1 × 10^−1^	[61]
β-Alanine ^[d]^	3.86	1.9 × 10^5^	3.1 × 10^−2^	[61]
Proline ^[c]^	3.57	5.4 × 10^5^	2.8 × 10^−2^	[62]
Lysine ^[b],[c]^	4.44 ^[e]^	1.7 × 10^4^	1.2 × 10^−1^	[63]
	3.24 ^[f]^	6.3 × 10^4^	5.1 × 10^−1^	

More data on alkyl- and aryl-amines are reported in Table S1. ^[a]^ pK_b_ values are from ref. [42,52,53,54,55,56,57,58,59,62,63]. For a list of pK_b_ see ref. [72,73]. (K_w_ = 10^−14.27^); ^[b]^ Measured at 25 °C; ^[c]^ Potassium salt; ^[d]^ Sodium salt; ^[e]^ Deprotonation of the α-amino group; ^[f]^ Deprotonation of the amino group on the lateral chain; 3-MPD = 3-methylpiperidine, 4-MPD = 4-methylpiperidine, MEA = monoethanolamine, DEA = diethanolamine, 1-AP = 2-amino-1-propanol, 2-AP = 1-amino-2-propanol, MPA = 3-amino-1-propanol, AMP = 2-amino-2-methyl-1-propanol, 4-PIPDM = 4-piperidinemethanol, 4-PIPDE = 4-piperidineethanol, MPIPZ = 1-methylpiperazine, 4-AMTHP = 4-aminomethyltetrahydropyran.

## Supplementary Materials

The following are available online at https://www.mdpi.com/1420-3049/25/16/3603/s1, Table S1: Rate (k) and equilibrium (K_CBM_) constants for the carbamato formation and equilibrium constant (K_HYD_) for the carbamato hydrolysis at 18 °C in water; Table S2: Selected X-ray bond distances (Å) and angles (°) in amidinium/guanidinium CO_2_ adducts; Table S3: Selected bond distances (Å) and angles (°) for carbamato ligands in metal complexes; Table S4: NMR and IR data related to the NCO_2_ moiety in structurally characterized metal carbamato complexes.
