# Supplementary material for: Recent Advances in the Chemistry of Metal Carbamates
Source: Molecules. 2020 Aug 7;25(16):3603. doi: 10.3390/molecules25163603 (PMC7465543; doi:10.3390/molecules25163603)
Supplement: Supplementary file 1 [file molecules-25-03603-s001.pdf]

# Recent Advances in the Chemistry of Metal Carbamates

**Giulio Bresciani, Lorenzo Biancalana, Guido Pampaloni and Fabio Marchetti**

Dipartimento di Chimica e Chimica Industriale, Università di Pisa, Via G. Moruzzi 13, I-56124 Pisa, Italy

# SUPPORTING INFORMATION

**Table of contents** **Page(s)**

**Table S1.** Rate and equilibrium constants for carbamato formation and hydrolysis in water. **S2-S3**

**Table S2.** Selected X-ray bond distances (Å) and angles (°) in amidinium/guanidinium $CO_2$ adducts. **S4**

**Table S3.** Selected bond distances (Å) and angles (°) for carbamato ligands in metal complexes. **S5-S9**

**Table S4.** NMR and IR data related to the $NCO_2$ moiety in metal carbamato complexes. **S10-S13**

**References** **S14-S23**

**Table S1.** Rate (k) and equilibrium ($K_{CBM}$) constants for the carbamato formation and equilibrium constant ($K_{HYD}$) for the carbamato hydrolysis at 18 °C in water.

$$CO_{2(aq)} + 2\ R_2NH_{(aq)} \underset{}{\overset{k,\ K_{CBM}}{\rightleftharpoons}} [R_2NH_2]^+_{(aq)} + [R_2NCO_2]^-_{(aq)}$$

$$[R_2NCO_2]^-_{(aq)} + H_2O_{(l)} \overset{K_{HYD}}{\rightleftharpoons} [R_2NH_2]^+_{(aq)} + HCO_3^-{}_{(aq)}$$

| Amine | $pK_b$[a] | *k* ($M^{-1}\ min^{-1}$) | $K_{CBM}$ | $K_{HYD}$ | Ref. |
|---|---|---|---|---|---|
| $NH_3$ | 4.76 | $3.8 \times 10^3$ | $2.3 \times 10^3$ | $4.4 \times 10^{-1}$ | [1] |
| | | *Primary amines* | | | |
| $NH_2Me$ | 3.38 | $8.3 \times 10^4$ | $4.0 \times 10^6$ | $6.0 \times 10^{-3}$ | [1] |
| $NH_2Et$ | 3.19 | $1.5 \times 10^5$ | $2.0 \times 10^6$ | $1.8 \times 10^{-2}$ | [1] |
| $NH_2Pr$ | 3.41 | $1.6 \times 10^5$ | $1.5 \times 10^6$ | $1.3 \times 10^{-2}$ | [1] |
| $NH_2{}^iPr$ | 3.37 | $6.8 \times 10^4$ | $3.6 \times 10^5$ | $6.3 \times 10^{-2}$ | [1] |
| $NH_2Bn$ | 4.74 | $1.1 \times 10^5$ | $8.5 \times 10^4$ | $1.2 \times 10^{-2}$ | [1] |
| $NH_2Ph$ | 9.30 | $\approx 10^3$ | $8.1 \times 10^{-3}$ | 3.6 | [1] |
| $NH_2$allyl | 4.38 | $1.1 \times 10^5$ | $1.6 \times 10^5$ | $1.5 \times 10^{-2}$ | [1] |
| $NH_2Bu$ | 3.24 | $2.0 \times 10^5$ | $1.1 \times 10^5$ | $1.6 \times 10^{-2}$ | [1] |
| $NH_2{}^sBu$ | 3.44 | $7.1 \times 10^4$ | $3.8 \times 10^5$ | $4.9 \times 10^{-2}$ | [1] |
| $NH_2{}^iBu$ | 3.58 | $1.6 \times 10^5$ | $1.3 \times 10^6$ | $1.0 \times 10^{-2}$ | [1] |
| $NH_2{}^tBu$ | 3.55 | $1.9 \times 10^4$ | $1.1 \times 10^5$ | $1.0 \times 10^{-1}$ | [1] |
| | | *Secondary amines* | | | |
| $NHMe_2$ | 3.22 | $6.7 \times 10^5$ | $1.6 \times 10^6$ | $2.2 \times 10^{-2}$ | [1] |
| $NHEt_2$ | 3.51 | $2.9 \times 10^5$ | $7.4 \times 10^4$ | $2.4 \times 10^{-1}$ | [1] |
| $NHPr_2$ | 3.09 | $3.0 \times 10^5$ | $3.0 \times 10^5$ | $1.5 \times 10^{-1}$ | [1] |
| $NH{}^iPr_2$ | 3.17 | – | None | – | [1] |
| $NHBu_2$ | 2.54 | $3.1 \times 10^5$ | $4.6 \times 10^5$ | $1.9 \times 10^{-1}$ | [1] |
| $NH{}^sBu_2$ | – | – | None | – | [1] |
| $NH{}^iBu_2$ | 3.41 | $1.3 \times 10^5$ | $1.9 \times 10^5$ | $1.2 \times 10^{-1}$ | [1] |
| Piperidine | 2.95 | $7.8 \times 10^5$ | $7.9 \times 10^5$ | $8.1 \times 10^{-2}$ | [1] |
| 3-MPD | 3.12 | – | $6.9 \times 10^6$ | $6.2 \times 10^{-3}$ | [2] |
| 4-MPD | 3.06 | – | $5.9 \times 10^6$ | $8.3 \times 10^{-3}$ | [2] |
| Pyrrolidine | 3.16 | $3.5 \times 10^6$ | $1.9 \times 10^7$ | $2.0 \times 10^{-3}$ | [3,4] |
| | | *Substituted amines* | | | |
| MEA | 4.42 | $8.3 \times 10^4$ | $6.0 \times 10^4$ | $1.9 \times 10^{-2}$ | [1] |
| DEA | 4.98 | $1.0 \times 10^5$ | $2.1 \times 10^3$ | $1.5 \times 10^{-1}$ | [1] |
| 1-AP[b] | 4.75 | $3.2 \times 10^5$ | $9.6 \times 10^3$ | $1.1 \times 10^{-1}$ | [3] |
| 2-AP[b] | 4.75 | $6.0 \times 10^4$ | $4.0 \times 10^3$ | $2.5 \times 10^{-1}$ | [5] |
| MPA[b] | 4.18 | $5.4 \times 10^5$ | $2.5 \times 10^5$ | $1.5 \times 10^{-2}$ | [5] |

| | | | | | |
|---|---|---|---|---|---|
| AMP[b] | 4.73 | – | None | – | [5] |
| 4-PIPDM[b] | 3.71 | $1.3 \times 10^{6}$ | $2.7 \times 10^{5}$ | $4.1 \times 10^{-2}$ | [3,4] |
| 4-PIPDE[b] | 3.65 | – | $3.0 \times 10^{5}$ | $4.2 \times 10^{-2}$ | [4] |
| Morpholine[b] | 5.78 | $1.6 \times 10^{5}$ | $1.4 \times 10^{3}$ | $6.8 \times 10^{-2}$ | [3,4] |
| Thiomorpholine[b] | 5.57 | $1.9 \times 10^{5}$ | $9.3 \times 10^{2}$ | $1.6 \times 10^{-1}$ | [3,4] |
| Piperazine | 4.50 | – | $5.5 \times 10^{4}$ | $3.2 \times 10^{-2}$ | [6] |
| | 8.67 | – | $2.6 \times 10^{-1}$ | $4.6 \times 10^{-1}$ | |
| MPIPZ | 4.96 | – | $5.1 \times 10^{3}$ | $1.2 \times 10^{-1}$ | [3] |
| 4-AMTHP[b] | 4.37 | $3.6 \times 10^{5}$ | $1.9 \times 10^{5}$ | $1.3 \times 10^{-2}$ | [7] |
| Taurine[b],[c] | 5.19 | – | $7.1 \times 10^{3}$ | $5.1 \times 10^{-2}$ | [8] |
| | | | ***Amino acids*** | | |
| Glycine[d] | 4.49 | $3.2 \times 10^{5}$ | $4.4 \times 10^{4}$ | $4.2 \times 10^{-2}$ | [9] |
| Sarcosine[b],[d] | 4.22 | $1.1 \times 10^{6}$ | $3.3 \times 10^{4}$ | $1.0 \times 10^{-1}$ | [10] |
| a-Alanine[d] | 4.40 | $6.6 \times 10^{4}$ | $1.8 \times 10^{4}$ | $1.1 \times 10^{-1}$ | [11] |
| b-Alanine[d] | 3.86 | $1.1 \times 10^{5}$ | $1.9 \times 10^{5}$ | $3.1 \times 10^{-2}$ | [11] |
| Proline[c] | 3.57 | – | $5.4 \times 10^{5}$ | $2.8 \times 10^{-2}$ | [12] |
| Lysine[b],[c] | 4.44[e] | – | $1.7 \times 10^{4}$ | $1.2 \times 10^{-1}$ | [13] |
| | 3.24[f] | – | $6.3 \times 10^{4}$ | $5.1 \times 10^{-1}$ | |

[a]$pK_b$ values are from ref. [1–9,12,13]. For a list of $pK_b$ see ref. [14,15]. ($K_w = 10^{-14.27}$); [b]Measured at 25°C; [c]Potassium salt; [d]Sodium Salt; [e]Deprotonation of the a-amino group; [f]Deprotonation of the amino group on the lateral chain; 3-MPD = 3-methylpiperidine, 4-MPD = 4-methylpiperidine, MEA = monoethanolamine, DEA = diethanolamine, 1-AP = 2-amino-1-propanol, 2-AP = 1-amino-2-propanol, MPA = 3-amino-1-propanol, AMP = 2-amino-2-methyl-1-propanol, 4-PIPDM = 4-piperidinemethanol, 4-PIPDE = 4-piperidineethanol, MPIPZ = 1-methylpiperazine, 4-AMTHP = 4-aminomethyltetrahydropyran

**Table S2.** Selected X-ray bond distances (Å) and angles (°) in amidinium/guanidinium $CO_2$ adducts.

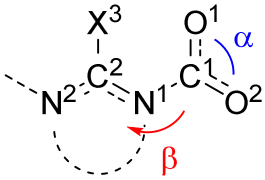


| Compound | $N^1$-$C^1$ (Å) | $C^1$-$O^1$ (Å) | $C^1$-$O^2$ (Å) | $N^1$-$C^2$ (Å) | $C^2$-$N^2$ (Å) | $C^2$-$N^3$ (Å) | $O^1$-$C^1$-$O^2$ (α) (°) | $C^1$-$N^1$-$C^2$-$N^2$ (β) (°) | Ref. |
|---|---|---|---|---|---|---|---|---|---|
| 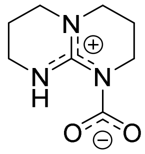 | 1.480(3) | 1.257(3) | 1.229(2) | 1.369(3) | 1.332(2) | 1.338(3) | 128.6(2) | 4.4(2) | [16] |
| 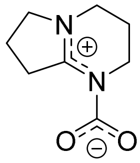 | 1.513(3) | 1.233(3) | 1.228(3) | 1.330(3) | 1.322(3) | - | 132.2(2) | 9.1(2) | [17] |
| 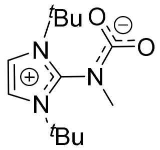 | 1.439(2) | 1.248(1) | 1.243(1) | 1.373(1) | 1.355(1) | 1.349(2) | 130.6(1) | 88.9(2) | [17] |
| 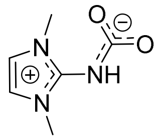 | 1.432(1) | 1.251(1) | 1.243(1) | 1.368(1) | 1.338(1) | 1.392(1) | 129.09(9) | 68.9(1) | [17] |

**Table S3.** Selected bond distances (Å) and angles (°) for carbamato ligands in metal complexes (2004-2020).

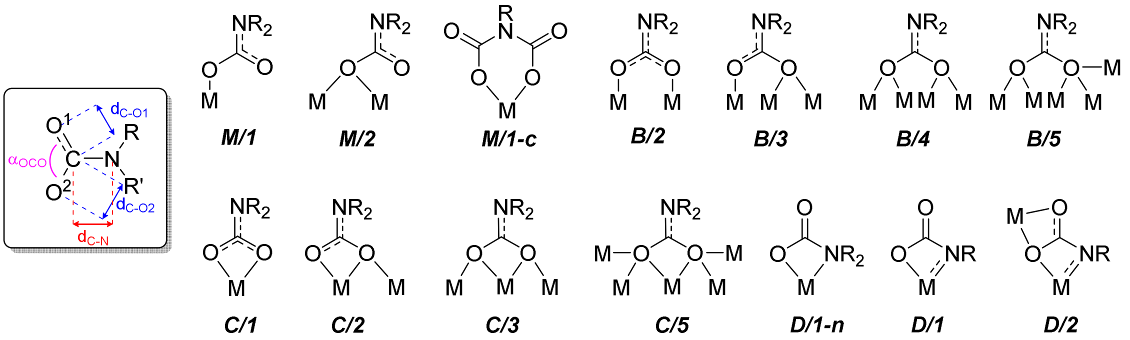


| Metal center | Carbamate coordination | C-O¹ (Å) | C-O² (Å) | C-N (Å) | O-C-O (°) | Ref. |
|---|---|---|---|---|---|---|
| Al(III) | *B/2* | 1.284(2) | 1.282(2) | 1.347(2) | 120.0(1) | [18] |
| Al(III) | *B/2* | 1.300(5) | 1.282(5) | 1.326(5) | 122.3(4) | [19] |
| Al(III) | *B/2* | 1.280(4) | 1.280(4) | 1.335(4) | 120.4(3) | [20] |
| Bi(III) | *M/1* | 1.295(5) | 1.250(6) | 1.348(4) | 123.9(4) | [21] |
| | *B/2* | 1.288(4) | 1.265(4) | 1.360(4) | 122.9(3) | |
| Bi(III) | *C/2* | 1.295(4) | 1.274(4) | 1.342(4) | 120.5(3) | [22] |
| | *C/1* | 1.299(4) | 1.260(4) | 1.355(4) | 119.9(3) | |
| | *C/1* | 1.28(1) | 1.26(2) | 1.35(2) | 120(1) | |
| | *B/2* | 1.28(1) | 1.26(1) | 1.34(1) | 121(1) | |
| Ce(III) | *C/2* | 1.28(1) | 1.28(1) | 1.35(1) | 119(1) | [23] |
| | *B/3* | 1.34(1) | 1.25(1) | 1.33(1) | 120.6(9) | |
| | *B/4* | 1.311(9) | 1.28(1) | 1.34(1) | 119.7(8) | |
| | *M/1* | 1.26(1) | 1.21(1) | 1.44(1) | 128.4(8) | |
| Ce(III) | *C/2* | 1.284(9) | 1.23(1) | 1.39(1) | 123.5(7) | |
| | *B/3* | 1.26(1) | 1.23(1) | 1.44(1) | 128.7(8) | |
| Ce(III) | *M/1* | 1.29(3) | 1.20(3) | 1.42(2) | 129.0(2) | [24] |
| Ce(IV) | *M/1* | 1.293(2) | 1.201(3) | 1.438(3) | 128.3(2) | |
| Ce(IV) | *B/2* | 1.253(4) | 1.243(4) | 1.421(4) | 128.1(2) | |
| | *C/1* | 1.269(8) | 1.254(7) | 1.357(5) | 118.3(5) | |
| Ce(IV) | *B/2* | 1.277(4) | 1.271(5) | 1.362(6) | 123.8(4) | [23] |
| | *C/2* | 1.279(7) | 1.270(4) | 1.346(7) | 119.6(4) | |
| Cu(II) | *M/1* | 1.30(3) | 1.20(3) | 1.39(3) | 131(2) | [25] |
| Cu(II) | *C/2* | 1.35(2) | 1.25(2) | 1.31(2) | 118(1) | [26] |
| Cu(II) | *B/2* | 1.269(6) | 1.259(6) | 1.375(6) | 126.7(4) | [27] |
| Cu(II) | *C/2* | 1.289(4) | 1.238(4) | 1.375(4) | 123.6(3) | |
| Cu(II) | *M/1* | 1.281(3) | 1.236(3) | 1.375(3) | 125.9(2) | [28] |
| | *B/2* | 1.280(3) | 1.280(3) | 1.367(3) | 125.2(2) | |

| | | | | | | |
|---|---|---|---|---|---|---|
| Dy(III) | ***C/1*** | 1.28(1) | 1.28(1) | 1.34(1) | 119.0(7) | [29] |
| | ***B/2*** | 1.275(8) | 1.27(1) | 1.36(1) | 122.7(7) | |
| | ***B/3*** | 1.293(9) | 1.25(1) | 1.372(8) | 121.3(6) | |
| Dy(III) | ***M/1*** | 1.272(6) | 1.235(7) | 1.373(8) | 125.0(5) | [30] |
| Er(III) | ***C/1*** | 1.295(8) | 1.260(9) | 1.369(9) | 120.5(6) | [31] |
| | ***B/2*** | 1.272(7) | 1.263(6) | 1.346(8) | 123.3(5) | |
| | ***B/3*** | 1.305(6) | 1.266(7) | 1.348(6) | 121.0(4) | |
| Er(III) | ***D/2*** | 1.333(6) | 1.277(6) | 1.315(6) | 115.6(4) | [32] |
| Eu(III) | ***C/1*** | 1.27(1) | 1.26(1) | 1.37(1) | 120.6(8) | [31] |
| | ***B/2*** | 1.278(8) | 1.254(6) | 1.336(7) | 123.1(5) | |
| | ***B/3*** | 1.300(7) | 1.253(6) | 1.340(8) | 122.2(5) | |
| Fe(III) | ***B/2*** | 1.280(5) | 1.269(5) | 1.346(6) | 125.3(4) | [33] |
| Fe(II) | ***M/1*** | 1.293(3) | 1.204(3) | 1.401(4) | 128.4(2) | [34] |
| Ga(III) | ***B/2*** | 1.283(4) | 1.281(5) | 1.345(5) | 122.2(3) | [35] |
| Ga(III) | ***C/1*** | 1.33(1) | 1.252(8) | 1.39(1) | 117.1(7) | [36] |
| | ***B/2*** | 1.30(1) | 1.30(1) | 1.37(1) | 122.3(7) | |
| Ga(III) | ***B/2*** | 1.283(3) | 1.276(3) | 1.354(3) | 121.0(2) | |
| Ga(III) | ***C/1*** | 1.293(3) | 1.290(3) | 1.351(3) | 114.7(2) | |
| Hf(IV) | ***C/1*** | 1.32(2) | 1.24(2) | 1.36(2) | 117(1) | [37] |
| | ***B/2*** | 1.29(1) | 1.26(2) | 1.31(2) | 121(1) | |
| Ir(I) | ***M/1*** | 1.300(9) | 1.23(1) | 1.36(1) | 125.5(8) | [38] |
| Ir(III) | ***M/1*** | 1.254(5) | 1.231(7) | 1.391(7) | 124.0(5) | [39] |
| Ir(III) | ***M/1*** | 1.252(7) | 1.251(6) | 1.401(8) | 124.8(5) | |
| Ir(III) | ***M/1*** | 1.26(1) | 1.22(1) | 1.395(8) | 124.2(7) | |
| Ir(III) | ***M/1*** | 1.275(3) | 1.219(2) | 1.474(3) | 128.7(2) | [40] |
| Ir(III) | ***C/1*** | 1.290(5) | 1.275(5) | 1.334(6) | 117.7(4) | [41] |
| Ir(III) | ***D/1*** | 1.366(4) | 1.237(4) | 1.327(4) | 120.7 | |
| La(III) | ***C/1*** | 1.25(1) | 1.25(1) | 1.39(1) | 122.0(9) | [42] |
| | ***C/2*** | 1.29(1) | 1.262(9) | 1.360(9) | 120.5(7) | |
| | ***C/3*** | 1.28(1) | 1.27(1) | 1.35(1) | 120.1(8) | |
| Li(I) | ***B/2*** | 1.261(2) | 1.255(2) | 1.401(2) | 124.3((1)) | [18] |
| Li(I) | ***B/2*** | 1.262(4) | 1.254(4) | 1.391(5) | 122.21(3) | |
| | ***B/3*** | 1.285(3) | 1.245(5) | 1.379(4) | 121.53(3) | |
| | ***B/4*** | 1.273(3) | 1.273(4) | 1.361(5) | 121.0(3) | |
| Li(I) | ***B/2*** | 1.264(4) | 1.262(4) | 1.338(5) | 123.1(3) | [43] |
| | ***B/3*** | 1.264(2) | 1.261(3) | 1.355(4) | 120.9(2) | |
| | ***B/5*** | 1.279(3) | 1.272(3) | 1.342(3) | 120.3(2) | |

| | | | | | | |
|---|---|---|---|---|---|---|
| | ***C/5*** | 1.261(3) | 1.251(4) | 1.333(3) | 121.2(3) | |
| Li(I) | ***B/3*** | 1.262(3) | 1.234(3) | 1.424(4) | 127.8(3) | |
| Lu(III) | ***C/1*** | 1.30(1) | 1.26(2) | 1.33(2) | 119(1) | [31] |
| | ***B/2*** | 1.26(1) | 1.25(1) | 1.36(1) | 124.0(9) | |
| | ***B/3*** | 1.309(9) | 1.24(1) | 1.34(1) | 121.6(8) | |
| Mg(II) | ***B/2*** | 1.267(3) | 1.266(3) | 1.368(3) | 124.9(2) | [44] |
| Mg(II) | ***B/2*** | 1.262(5) | 1.259(5) | 1.391(5) | 126.5(3) | |
| | ***C/3*** | 1.276(4) | 1.272(4) | 1.353(5) | 119.8(3) | |
| Mg(II) | ***B/2*** | 1.28(2) | 1.24(2) | 1.41(2) | 124.5(2) | [45] |
| | ***C/2*** | 1.30(2) | 1.22(2) | 1.368 | 124.0(1) | |
| | ***B/3*** | 1.28(2) | 1.28(2) | 1.32(2) | 122.0(1) | |
| | ***M/2*** | 1.28(2) | 1.28(2) | 1.35(2) | 124.3(1) | |
| Mn(II)/Mn(III) | ***B/2*** | 1.261(9) | 1.25(1) | 1.348(9) | 123.7(6) | [46] |
| | ***B/3*** | 1.267(5) | 1.258(4) | 1.333(4) | 122.6(3) | |
| Nb(V) | ***C/1*** | 1.306(6) | 1.295(8) | 1.323(9) | 115.3(5) | [47] |
| | ***B/2*** | 1.304(7) | 1.278(8) | 1.33(1) | 120.8(6) | |
| Nd(III) | ***C/2*** | 1.307(5) | 1.250(6) | 1.347(6) | 119.1(4) | [48] |
| Ni(II) | ***B/2*** | 1.264(3) | 1.255(3) | 1.368(3) | 129.0(2) | [49] |
| Ni(II) | ***B/2*** | 1.27(2) | 1.22(2) | 1.44(2) | 130(1) | |
| Ni(II) | ***M/1*** | 1.263(8) | 1.24(1) | 1.37(1) | 127.3(6) | [50] |
| Ni(II) | ***M/1*** | 1.286(4) | 1.254(3) | 1.361(4) | 124.6(3) | [51] |
| Ni(II) | ***D/1*** | 1.340(2) | 1.229(2) | 1.360(2) | 123.7(2) | [52] |
| Ni(II) | ***B/2*** | 1.325(7) | 1.322(8) | 1.291(8) | 118.6(5) | [53] |
| Ni(II) | ***M/1*** | 1.288(2) | 1.243(2) | 1.372(2) | 124.7(1) | [54] |
| Ni(II) | ***M/1*** | 1.280(3) | 1.211(4) | 1.388(4) | 126.5(3) | [55] |
| Ni(II) | ***M/1*** | 1.288(2) | 1.233(2) | 1.387(2) | 126.5(1) | [56] |
| Ni(II) | ***C/1*** | 1.284(4) | 1.239(4) | 1.385(4) | 124.2(3) | |
| Pd(II) | ***D/1*** | 1.316(2) | 1.219(2) | 1.401(3) | 124.2(2) | [57] |
| Pd(II) | ***M/1*** | 1.299(4) | 1.230(5) | 1.382(5) | 125.0(3) | [58] |
| Pt(IV) | ***M/1*** | 1.326(4) | 1.235(4) | 1.354(4) | 125.1(3) | [59] |
| Pt(IV) | ***M/1*** | 1.323(3) | 1.240(2) | 1.346(3) | 125.4(2) | |
| Pt(IV) | ***M/1*** | 1.335(4) | 1.233(4) | 1.344(4) | 125.0(3) | |
| Pt(IV) | ***M/1*** | 1.325(5) | 1.219(5) | 1.354(6) | 126.1(4) | |
| Pt(IV) | ***M/1*** | 1.321(3) | 1.236(3) | 1.340(3) | 125.8(2) | [60] |
| Pt(IV) | ***M/1*** | 1.310(4) | 1.242(5) | 1.346(5) | 124.6(4) | [61] |
| Pt(IV) | ***M/1*** | 1.327(4) | 1.249(5) | 1.336(6) | 125.0(3) | |
| Re(I) | ***M/1*** | 1.27(1) | 1.25(1) | 1.40(1) | 125.1(7) | [62] |

| | | | | | | |
|---|---|---|---|---|---|---|
| Rh(III) | ***C/1*** | 1.281(4) | 1.263(4) | 1.352(4) | 119.6(3) | [63] |
| Ru(II) | ***C/1*** | 1.28(3) | 1.24(3) | 1.39(4) | 123(3) | [64] |
| Ru(II) | ***M/1*** | 1.301(5) | 1.229(5) | 1.457(6) | 127.3(4) | [65] |
| Ru(II) | ***M/1*** | 1.281(2) | 1.243(2) | 1.390(2) | 126.5(1) | [66] |
| Sc(III) | ***M/1-c*** | 1.318(5) | 1.209(5) | 1.425(5) | 124.3(4) | [67] |
| Sm(III) | ***C/1*** | 1.289(6) | 1.255(7) | 1.364(9) | 120.4(5) | [23] |
| | ***B/2*** | 1.273(4) | 1.270(6) | 1.358(6) | 123.4(4) | |
| | ***B/3*** | 1.304(4) | 1.262(4) | 1.345(6) | 121.6(3) | |
| Sn(II) | ***M/1*** | 1.304(3) | 1.238(3) | 1.366(2) | 121.7(2) | [68] |
| Sn(II) | ***C/1*** | 1.280(3) | 1.272(3) | 1.338(3) | 118.8(2) | [69] |
| | ***B/2*** | 1.295(3) | 1.263(3) | 1.336(3) | 121.8(2) | |
| Tb(III) | ***C/1*** | 1.30(2) | 1.25(2) | 1.36(3) | 121.0(2)) | [70] |
| | ***B/2*** | 1.27(2) | 1.26(2) | 1.38(2) | 124.0(1) | |
| Ti(III) | ***B/2*** | 1.281(4) | 1.268(4) | 1.371(4) | 123.7(3) | [71] |
| Ti(IV) | ***C/1*** | 1.290(5) | 1.277(5) | 1.346(6) | 116.0(4) | [72] |
| Ti(IV) | ***C/1*** | 1.294(6) | 1.279(4) | 1.333(5) | 116.7(3) | [73] |
| Ti(IV) | ***D/1*** | 1.340(7) | 1.212(7) | 1.378(9) | 123.4(6) | [74] |
| Ti(IV) | ***M/1-c*** | 1.308(4) | 1.212(4) | 1.402(5) | 122.9(3) | [75] |
| Ti(IV) | ***B/2*** | 1.295(4) | 1.280(4) | 1.330(6) | 122.6(4) | [37] |
| U(IV) | ***C/1*** | 1.296(5) | 1.268(5) | 1.335(6) | 119.0(3) | [76] |
| U(IV) | ***M/1*** | 1.287(4) | 1.196(5) | 1.375(6) | 127.0(3) | [77] |
| U(IV) | ***C/1*** | 1.279(7) | 1.258(6) | 1.382(7) | 121.1(5) | |
| U(IV) | ***C/1*** | 1.283(9) | 1.262(8) | 1.344(8) | 120.6(5) | [78] |
| U(IV) | ***M/1-c*** | 1.317(4) | 1.229(6) | 1.396(6) | 121.8(4) | [79] |
| U(IV) | ***C/1*** | 1.273(7) | 1.263(6) | 1.382(7) | 120.3(5) | [80] |
| U(IV) | ***D/2*** | 1.349(4) | 1.285(5) | 1.304(5) | 115.8(3) | [81] |
| U(VI) | ***B/2*** | 1.33(3) | 1.25(3) | 1.43(3) | 123(2) | [82] |
| W(0) | ***D/1-n*** | 1.283(3) | 1.208(3) | 1.513(3) | 131.0(2) | [83] |
| Y(III) | ***D/2*** | 1.328(8) | 1.292(7) | 1.313(7) | 115.4(5) | [32] |
| Yb(III) | ***C/1*** | 1.27(1) | 1.26(1) | 1.35(1) | 119.1(9) | [31] |
| | ***B/2*** | 1.27(1) | 1.263(9) | 1.356(8) | 123.4(7) | |
| | ***B/3*** | 1.307(7) | 1.243(7) | 1.357(9) | 122.6(6) | |
| Yb(III) | ***D/2*** | 1.32(1) | 1.28(1) | 1.31(1) | 115.6(8) | [32] |
| Yb(III) | ***C/2*** | 1.308(6) | 1.240(6) | 1.352 (6) | 118.6(5) | [48] |
| Zn(II) | ***M/1*** | 1.276(5) | 1.245(5) | 1.357(6) | 124.8(4) | [28] |
| Zn(II) | ***B/2*** | 1.27(1) | 1.24(1) | 1.35(1) | 125.5(9) | [84] |
| Zn(II) | ***B/2*** | 1.268(3) | 1.265(4) | 1.352(3) | 124.4(4) | [85] |

| | | | | | | |
|---|---|---|---|---|---|---|
| Zn(II) | ***B/3*** | 1.299(3) | 1.261(3) | 1.343(3) | 122.0(2) | [86] |
| Zn(II) | ***B/3*** | 1.296(4) | 1.273(5) | 1.336(5) | 122.5(3) | |
| Zn(II) | ***B/2*** | 1.272(2) | 1.269(2) | 1.355(2) | 122.3(2) | |
| Zn(II) | ***B/2*** | 1.272(8) | 1.268(7) | 1.359(7) | 125.9(6) | [87] |
| Zn(II) | ***M/1*** | 1.286(7) | 1.225(9) | 1.401(8) | 124.0(6) | [88] |
| Zn(II) | ***M/1*** | 1.290(3) | 1.236(3) | 1.379(4) | 125.9(2) | [89] |
| Zn(II) | ***B/2*** | 1.276(3) | 1.273(3) | 1.360(3) | 122.7(2) | |
| Zn(II) | ***B/2*** | 126.8(3) | 126.8(3) | 1.364(5) | 125.7(4) | [90] |
| Zn(II) | ***B/2*** | 1.257(6) | 1.241(6) | 1.353(7) | 124.3(5) | [91] |
| Zn(II) | ***M/1*** | 1.287(6) | 1.230(7) | 1.404(7) | 125.0(5) | [92] |
| Zn(II) | ***B/2*** | 1.276(4) | 1.264(3) | 1.368(3) | 123.8(3) | [93] |
| Zn(II) | ***M/1*** | 1.287(4) | 1.249(5) | 1.380(5) | 126.2(3) | |
| Zn(II) | ***B/2*** | 1.269(3) | 1.259(4) | 1.372(4) | 124.9(3) | |
| | ***B/3*** | 1.294(3) | 1.253(4) | 1.355(4) | 123.3(3) | |
| Zn(II) | ***B/2*** | 1.276(5) | 1.259(4) | 1.371(4) | 124.2(3) | [94] |
| Zn(II) | ***B/2*** | 1.271 | 1.271 | 1.444 | 124.4 | [95] |
| Zn(II)/Dy(III) | ***B/2*** | 1.30(2) | 1.26(2) | 1.32(2) | 125.0(1) | [96] |
| Zn(II)/Tb(III) | ***B/2*** | 1.28(1) | 1.27(1) | 1.345(9) | 125.6(8) | |
| Zn(II)/Gd(III) | ***B/2*** | 1.28(1) | 1.27(1) | 1.34(1) | 125.0(1) | |
| Zr(IV) | ***B/2*** | 1.292(5) | 1.277(4) | 1.332(4) | 119.5(3) | [97] |

**Table S4.** NMR and IR data related to the $NCO_2$ moiety in structurally characterized metal carbamato complexes.

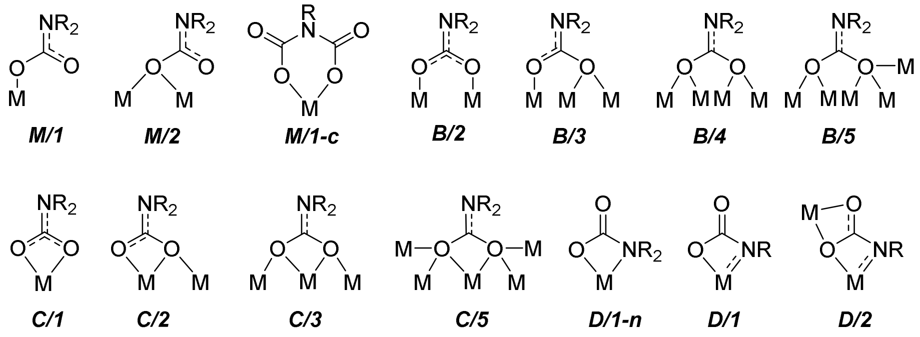


| Metal center | Carbamate coordination | $^{13}C\{^{1}H\}$ NMR: δ/ppm [a] | IR: $\tilde{\upsilon}$/cm$^{-1}$ [b] | Ref. |
|---|---|---|---|---|
| Ag(I) | *C/1* | | **1529**, 1478, 1434, 1414, 1368 | [1] |
| Ag(I) | *B/3* | | **1554**, 1520, 1466, 1386 | |
| Al(III) | *B/2* | 158.0 | **1595**, 1506 | [19] |
| Au(I) | *M/1* | 161 | **1578** | [98] |
| Au(I) | *M/1* | | **1601**, 1580, 1569, 1478, 1468, 1435, 1407, 1371 | [1] |
| Bi(III) | *B/2 ,C/2 ,C/1* | 163.6 | | [22] |
| Ce(III) | *C/1, B/2, C/2, B/3, B/4* | | **1576**, 1558, 1507, 1354, 1260 | [23] |
| Ce(III) | *M/1, C/2, B/3* | | **1725**, 1683, 1378, 1347, 1286 | [24] |
| Ce(IV) | *M/1* | 149.9 | **1732**, 1457, 1336 | |
| Ce(IV) | *C/1, B/2, C/2* | | **1562**, 1538, 1506, 1350, 1261 | [23] |
| Co(III) | *M/1* | 165.0 | | [99] |
| Cr(II) | *B/2* | | **1578**, 1560, 1510, 1498, 1460, 1433, 1378, 1318 | [1] |
| Cr(III) | *C/1* | | **1485**, 1378, 1337, 1322 | |
| Cu(II) | *M/1* | | **1570**, 1496, 1462, 1452, 1408, 1377, 1311 | |
| Cu(II) | *B/2* | | **1575**, 1550, 1480, 1460, 1420, 1375, 1300 | |
| Dy(III) | *M/1* | | **1658**, 1597, 1554, 1520, 1456, 1390, 1269 | [30] |
| Er(III) | *C/1, B/2, B/3* | | **1602**, 1540, 1490, 1355 | [31] |
| Eu(III) | *C/1, B/2, B/3* | | **1597**, 1533, 1488, 1354 | |
| Er(III) | *D/2* | | **1607**, 1580, 1502, 1260 | [32] |
| Fe(II) | *M/1* | 156 | **1636**, 1304 | [100] |
| Fe(II) | *M/1* | 162.2 | | [34] |
| Ga(III) | *B/2* | 165.0 | **1543**, 1452, 1342, 1317 | [35] |
| Ga(III) | *C/1* | 165.8 | | [36] |
| | *B/2* | 161.3 | | |
| Ga(III) | *B/2* | 165.3 | | |
| Ga(III) | *C/1* | 168.2 | | |

| | | | | |
|---|---|---|---|---|
| Gd(III) | ***C/1, B/2, B/3*** | | **1598**, 1537, 1485, 1355 | [31] |
| Hf(IV) | ***C/1*** | 170.2 | | [1] |
| Hf(IV) | ***C/1*** | | **1540**, 1510, 1480, 1350 | |
| Hf(IV) | ***C/1*** | | **1540**, 1510, 1480, 1350 | |
| Ho(III) | ***C/1, B/2, B/3*** | | **1601**, 1538, 1489, 1354 | [31] |
| Ir(III) | ***M/1*** | 161.7 | | [40] |
| Ir(I) | ***M/1*** | 163.2 | | [38] |
| Ir(I) | ***M/1*** | 159.7 | | |
| Li(I) | ***B/2*** | 167.2 | - | [18] |
| Li(I) | ***B/2, B/3, B/5, C/5*** | | **1594**, 1545, 1360, 1265 | [43] |
| Li(I) | ***B/3*** | 155.1 | **1623**, 1261 | |
| Lu(III) | ***C/1, B/2, B/3*** | | **1610**, 1530, 1490, 1354 | [31] |
| Mg(II) | ***B/2*** | 161.5 | | [44] |
| Mg(II) | ***B/2, C/3*** | 159.8 | | |
| | | 159.3 | | |
| Mg(II) | ***B/2, C/2, B/3, M/2*** | 162.8 | **1611**, 1582, 1515, 1283 | [45] |
| Mo(II) | ***B/2*** | | **1510**, 1435, 1380, 1315 | [1] |
| Nb(IV) | ***C/1*** | | **1558**, 1505, 1483, 1441, 1377, 1348 | |
| Nb(V) | ***C/1*** | 168.1 | **1620**, 1576, 1558, 1484, 1433 | [47] |
| | ***B/2*** | 162.0 | | |
| Nb(V) | ***C/1 , M/1*** | 162.3 | | [1] |
| Nd(III) | ***C/1, B/2, B/3*** | | **1593**, 1515, 1479, 1354 | [31] |
| Nd(III) | ***C/2*** | | **1616**, 1590, 1538, 1445, 1337 | [48] |
| Ni(II) | ***M/1*** | 161.2 | **1681** | [101] |
| Ni(II) | ***B/2*** | | **1597** | [49] |
| Ni(II) | ***B/2*** | | **1564** | |
| Ni(II) | ***M/1*** | 162.3 | **1629**, 1351 | [50] |
| Ni(II) | ***M/1*** | 160.6 | | [51] |
| Ni(II) | ***D/1*** | 170.5 | **1617** | [52] |
| | ***M/1*** | 157.9 | **1667**, 1624 | |
| Ni(II) | ***M/1*** | 159.6 | **1610** | [54] |
| Ni(II) | ***M/1*** | 162.9 | | [102] |
| Ni(II) | ***M/1*** | 157.3 | | [55] |
| Ni(II) | ***M/1*** | 158.3 | | [103] |
| Ni(II) | ***M/1*** | 159.7 | **1625** | [56] |
| Ni(II) | ***C/1*** | 160.6 | **1620** | [56] |
| Pd(II) | ***M/1*** | 150.6 | | [58] |

| | | | | |
|---|---|---|---|---|
| Pd(II) | ***D/1*** | 165.3 | **1677** | [57] |
| Pd(II) | ***M/1*** | | **1590**, 1555, 1475, 1455, 1410, 1375, 1325 | [1] |
| Pr(III) | ***C/1, B/2, B/3*** | | **1591**, 1527, 1518, 1353 | [31] |
| Pt(II) | ***M/1*** | | **1632** | [104] |
| Pt(II) | ***M/1*** | | **1586**, 1563, 1474, 1458, 1411, 1374, 1326 | [1] |
| Pt(IV) | ***M/1*** | 162.8 | **1640**, 1629 | [59] |
| Pt(IV) | ***M/1*** | 163.4 | **1629** | |
| Pt(IV) | ***M/1*** | 163.0 | **1628** | |
| Pt(IV) | ***M/1*** | 160.7 | **1654** | |
| Pt(IV) | ***M/1*** | 165.4 | | [60] |
| Pt(IV) | ***M/1*** | 164.8 | | [61] |
| Pt(IV) | ***M/1*** | 163.9 | | |
| Rh(III) | ***C/1*** | 165.1 | | [63] |
| Ru(II) | ***C/1*** | 164.0 | | [64] |
| Ru(II) | ***M/1*** | 155 | **1634**, 1602 | [65] |
| Ru(II) | ***M/1*** | 165.03, 163.37 | **1587** | [66] |
| Ru(II) | ***M/1*** | | **1594**, 1570, 1485, 1434, 1425, 1370, 1337 | [1] |
| Ru(II) | ***C/1*** | | **1565**, 1465, 1412 | |
| Ru(II) | ***C/1*** | | **1556**, 1460, 1438, 1380 | |
| Ru(II) | ***C/1*** | | **1505**, 1465, 1436, 1378, 1366, 1352 | |
| Sm(III) | ***C/1, B/2, B/3*** | | **1595**, 1531, 1508, 1353 | [105] |
| Sn(II) | ***M/1*** | 161.8 | **1595**, 1575, 1552, 1524, 1337 | [68] |
| Sn(II) | ***M/1*** | 161.6 | **1624,** 1554, 1526, 1517, 1238 | |
| Sn(II) | ***C/1, B/2*** | 164.5 | **1539**, 1471, 1384, 1259 | [69] |
| Ta(V) | ***C/1 , M/1*** | 161.7 | | [1] |
| Tb(III) | ***C/1, B/2*** | | **1584**, 1487, 1374, 1313, 1261 | [70] |
| Ti(IV) | ***C/1*** | | **1589**, 1512, 1503, 1408, 1266 | [72] |
| Ti(IV) | ***C/1*** | 168.9 | **1547**, 1501, 1475, 1455, 1346 | [73]. |
| Ti(IV) | ***D/1*** | 175.3 | **1666**, 1514, 1356, 1306 | [74] |
| Ti(IV) | ***M/1-c*** | 152.8 | **1657**, 1624, 1590, 1558, 1524, 1500, 1407 | [75] |
| Ti(IV) | ***B/2*** | 160.8 | **1538**, 1473, 1434, 1379, 1299, | [37] |
| Ti(IV) | ***C/1*** | 169.9 | | [1] |
| Ti(IV) | ***C/1*** | 169.0 | | |
| Ti(IV) | ***C/1*** | 170.6 | **1550**, 1500, 1460, 1320 | |
| Ti(IV) | ***C/1*** | | **1597**, 1575, 1496s, 1410vs | |

| | | | | |
|---|---|---|---|---|
| U(IV) | ***C*/1** | | **1588**, 1509,1456, 1448, 1421 | [76] |
| U(IV) | ***M*/1** | | **1654** | [77] |
| U(IV) | ***C*/1** | | **1492**, 1254 | [78] |
| U(IV) | ***M*/1-*c*** | -134.2 | **1579**, 1648, 1645. | [79] |
| U(IV) | ***C*/1** | | **2859**, 1451, 1378, 1290 | [80] |
| W(0) | ***D*/1-*n*** | 159.3 | **1741** | [83] |
| W(VI) | ***M*/1** | | **1636** | [1] |
| Y(III) | ***D*/2** | | **1607**, 1585, 1502, 1260 | [32] |
| Yb(III) | ***C*/1, *B*/2, *B*/3** | | **1604**, 1541, 1491, 1355 | [31] |
| Yb(III) | ***D*/2** | | **1604**, 1584, 1533, 1266 | [32] |
| Yb(III) | ***C*/2** | | **1617**, 1591, 1538, 1446, 1338 | [48] |
| Zn(II) | ***B*/2** | 163.9 | **1538**, 1402 | [84] |
| Zn(II) | ***B*/3** | 163.50 | | [86] |
| Zn(II) | ***B*/3** | 164.7 | | |
| Zn(II) | ***B*/2** | 163.2 | | |
| Zn(II) | ***B*/2** | 162.0 | | [91] |
| Zn(II) | ***M*/1** | 162 [c] | **1616**, 1596, 1348, 1288 | [92] |
| Zn(II) | ***B*/2** | 162.5, 163.1 | **1606**, 1564 | [93] |
| Zn(II) | ***M*/1** | 161.0 | **1587**, 1549 | |
| Zn(II) | ***B*/2, *B*/3** | 162.8 | **1553**, 1485 | |
| Zn(II) | ***B*/2** | 166.0 | 1603, 1592 | [94] |
| Zn(II) | ***B*/2** | | 1538, 1461, 1402 | [1] |
| Zn(II) | ***B*/2** | | 1570, 1510, 1460, 1430, 1380, 1320 | |
| Zn(II) | ***B*/2** | | 1570, 1504, 1430, 1378, 1326 | |
| Zn(II)/Dy(III) | ***B*/2** | | **1638** | [96] |
| Zn(II)/Tb(III) | ***B*/2** | | **1642** | |
| Zn(II)/Gd(III) | ***B*/2** | | **1640** | |
| Zr(IV) | ***B*/2** | 162.7 | **1757** | [97] |
| Zr(IV) | ***C*/1** | 170.4 | **1565**, 1505, 1450, 1380, 1325 | [1] |
| Zr(IV) | ***C*/1** | 170.3 | **1535**, 1500, 1380, 1360 | |

[a] Room temperature NMR in $CDCl_3$ or other solvent; [b] Solid-state IR data; 1800-1300 cm-1 range, weak bands not included. The highest-wavenumber absorption in bold.

**References.**


1. Dell'Amico, D.B.; Calderazzo, F.; Labella, L.; Marchetti, F.; Pampaloni, G. Converting Carbon Dioxide into Carbamato Derivatives. *Chem. Rev.* **2003**, *103*, 3857–3898, doi:10.1021/cr940266m.

2. Coulier, Y.; Lowe, A.R.; Coxam, J.Y.; Ballerat-Busserolles, K. Thermodynamic Modeling and Experimental Study of CO2 Dissolution in New Absorbents for Post-Combustion CO2 Capture Processes. *ACS Sustain. Chem. Eng.* **2018**, *6*, 918–926, doi:10.1021/acssuschemeng.7b03280.

3. Fernandes, D.; Conway, W.; Burns, R.; Lawrance, G.; Maeder, M.; Puxty, G. Investigations of primary and secondary amine carbamate stability by 1H NMR spectroscopy for post combustion capture of carbon dioxide. *J. Chem. Thermodyn.* **2012**, *54*, 183–191, doi:10.1016/j.jct.2012.03.030.

4. Conway, W.; Wang, X.; Fernandes, D.; Burns, R.; Lawrance, G.; Puxty, G.; Maeder, M. Toward rational design of amine solutions for PCC applications: The kinetics of the reaction of CO2(aq) with cyclic and secondary amines in aqueous solution. *Environ. Sci. Technol.* **2012**, *46*, 7422–7429, doi:10.1021/es300541t.

5. Conway, W.; Wang, X.; Fernandes, D.; Burns, R.; Lawrance, G.; Puxty, G.; Maeder, M. Toward the understanding of chemical absorption processes for post-combustion capture of carbon dioxide: Electronic and steric considerations from the kinetics of reactions of CO2(aq) with sterically hindered amines. *Environ. Sci. Technol.* **2013**, *47*, 1163–1169, doi:10.1021/es3025885.

6. Ermatchkov, V.; Pérez-Salado Kamps, Á.; Maurer, G. Chemical equilibrium constants for the formation of carbamates in (carbon dioxide + piperazine + water) from 1H-NMR-spectroscopy. *J. Chem. Thermodyn.* **2003**, *35*, 1277–1289, doi:10.1016/S0021-9614(03)00076-4.

7. Li, L.; Clifford, S.; Puxty, G.; Maeder, M.; Burns, R.; Yu, H.; Conway, W. Kinetic and Equilibrium Reactions of a New Heterocyclic Aqueous 4-Aminomethyltetrahydropyran (4-AMTHP) Absorbent for Post Combustion Carbon Dioxide (CO2) Capture Processes. *ACS Sustain. Chem. Eng.* **2017**, *5*, 9200–9206, doi:10.1021/acssuschemeng.7b02149.

8. Kumar, P.S.; Hogendoorn, J.A.; Timmer, S.J.; Feron, P.H.M.; Versteeg, G.F. Equilibrium Solubility of CO 2 in Aqueous Potassium Taurate Solutions: Part 2. Experimental VLE Data and Model. *Ind. Eng. Chem. Res.* **2003**, *42*, 2841–2852, doi:10.1021/ie020601u.

9. Jensen, A.; Jensen, J.B.; Faurholt, C.; Finsnes, E.; Sörensen, J.S.; Sörensen, N.A. Studies on Carbamates. VI. The Carbamate of Glycine. *Acta Chem. Scand.* **1952**, *6*, 395–397, doi:10.3891/acta.chem.scand.06-0395.

10. Xiang, Q.; Fang, M.; Yu, H.; Maeder, M. Kinetics of the reversible reaction of CO2(aq) and

HCO 3- with sarcosine salt in aqueous solution. *J. Phys. Chem. A* **2012**, *116*, 10276–10284, doi:10.1021/jp305715q.

11. Jensen, A.; Faurholt, C.; Faurholt, C.; Finsnes, E.; Sörensen, J.S.; Sörensen, N.A. Studies on Carbamates. V. The Carbamates of alpha-Alanine and beta-Alanine. *Acta Chem. Scand.* **1952**, *6*, 385–394, doi:10.3891/acta.chem.scand.06-0385.

12. Majchrowicz, M.E.; Brilman, D.W.F. Solubility of CO 2 in aqueous potassium l-prolinate solutions-absorber conditions. *Chem. Eng. Sci.* **2012**, *72*, 35–44, doi:10.1016/j.ces.2011.12.014.

13. Shen, S.; Zhao, Y.; Bian, Y.; Wang, Y.; Guo, H.; Li, H. CO2 absorption using aqueous potassium lysinate solutions: Vapor – liquid equilibrium data and modelling. *J. Chem. Thermodyn.* **2017**, *115*, 209–220, doi:10.1016/j.jct.2017.07.041.

14. Fernandes, D.; Conway, W.; Wang, X.; Burns, R.; Lawrance, G.; Maeder, M.; Puxty, G. Protonation constants and thermodynamic properties of amines for post combustion capture of CO 2. *J. Chem. Thermodyn.* **2012**, *51*, 97–102, doi:10.1016/j.jct.2012.02.031.

15. Christensen, J.J.; Izatt, R.M.; Wrathall, D.P.; Hansen, L.D. Thermodynamics of proton ionization in dilute aqueous solution. Part XI. pK, ΔH°, and ΔS° values for proton ionization from protonated amines at 25°. *J. Chem. Soc. A* **1969**, 1212–1223, doi:10.1039/J19690001212.

16. Villiers, C.; Dognon, J.-P.; Pollet, R.; Thuéry, P.; Ephritikhine, M. An Isolated CO2 Adduct of a Nitrogen Base: Crystal and Electronic Structures. *Angew. Chem. Int. Ed.* **2010**, *49*, 3465–3468, doi:10.1002/anie.201001035.

17. Wilm, L.F.B.; Eder, T.; Mück-Lichtenfeld, C.; Mehlmann, P.; Wünsche, M.; Buß, F.; Dielmann, F. Reversible CO 2 fixation by N-heterocyclic imines forming water-stable zwitterionic nitrogen-base-CO 2 adducts. *Green Chem.* **2019**, *21*, 640–648, doi:10.1039/c8gc02952k.

18. Kennedy, A.R.; Mulvey, R.E.; Oliver, D.E.; Robertson, S.D. Lithium and aluminium carbamato derivatives of the utility amide 2,2,6,6-tetramethylpiperidide. *Dalton Trans.* **2010**, *39*, 6190–6197, doi:10.1039/c0dt00118j.

19. Marchetti, F.; Pampaloni, G.; Patil, Y.; Galletti, A.M.R.; Renili, F.; Zacchini, S. Ethylene Polymerization by Niobium(V) N,N -Dialkylcarbamates Activated with Aluminum Co-catalysts. *Organometallics* **2011**, *30*, 1682–1688, doi:10.1021/om101187k.

20. Habereder, T.; Nöth, H.; Paine, R.T. Synthesis and Reactivity of New Bis(tetramethylpiperidino)(phosphanyl)alumanes. *Eur. J. Inorg. Chem.* **2007**, *2007*, 4298–4305, doi:10.1002/ejic.200700415.

21. Yin, S.-F.; Maruyama, J.; Yamashita, T.; Shimada, S. Efficient Fixation of Carbon Dioxide by Hypervalent Organobismuth Oxide, Hydroxide, and Alkoxide. *Angew. Chem. Int. Ed.* **2008**, *47*, 6590–6593, doi:10.1002/anie.200802277.

22. Cosham, S.D.; Hill, M.S.; Horley, G.A.; Johnson, A.L.; Jordan, L.; Molloy, K.C.; Stanton, D.C. Synthesis and materials chemistry of bismuth Tris-(di-i-propylcarbamate): Deposition of photoactive Bi2O3 thin films. *Inorg. Chem.* **2014**, *53*, 503–511, doi:10.1021/ic402499r.

23. Baisch, U.; Dell' Amico, D.B.; Calderazzo, F.; Labella, L.; Marchetti, F.; Vitali, D. Reaction of a tetranuclear N,N-di-iso-propylcarbamato complex of cerium(III) with dioxygen: Synthesis and X-ray characterization of both the oxidation product and its precursor. *J. Mol. Catal. A Chem.* **2003**, *204–205*, 259–265, doi:10.1016/S1381-1169(03)00307-8.

24. Bayer, U.; Werner, D.; Maichle-Mössmer, C.; Anwander, R. Effective and Reversible Carbon Dioxide Insertion into Cerium Pyrazolates. *Angew. Chem. Int. Ed.* **2020**, *59*, 5830–5836, doi:10.1002/anie.201916483.

25. García-España, E.; Gaviña, P.; Latorre, J.; Soriano, C.; Verdejo, B. CO2 Fixation by Copper(II) Complexes of a Terpyridinophane Aza Receptor. *J. Am. Chem. Soc.* **2004**, *126*, 5082–5083, doi:10.1021/ja039577h.

26. Bramsen, F.; Bond, A.D.; McKenzie, C.J.; Hazell, R.G.; Moubaraki, B.; Murray, K.S. Self-Assembly of the Octanuclear Cluster [Cu8(OH)10(NH2(CH2)2CH3)12]6+ and the One-DimensionalN-Propylcarbamate-Linked Coordination Polymer {[Cu(O2CNH(CH2)2CH3)(NH2(CH2)2CH3)3](ClO4)}n. *Chem. -Eur. J.* **2005**, *11*, 825–831, doi:10.1002/chem.200400555.

27. Belli Dell'Amico, D.; Di Giacomo, A.; Falchi, L.; Labella, L.; Marelli, M.; Evangelisti, C.; Lezzerini, M.; Marchetti, F.; Samaritani, S. A convenient preparation of La2CuO4from molecular precursors. *Polyhedron* **2017**, *123*, 33–38, doi:10.1016/j.poly.2016.11.020.

28. Bedeković, N.; Stilinović, V. Morpholine-N-carboxylate as a ligand in coordination chemistry – Syntheses and structures of three heteroleptic copper(ii) and zinc complexes. *J. Mol. Struct.* **2020**, *1205*, 127627, doi:10.1016/j.molstruc.2019.127627.

29. Pineda, E.M.; Lan, Y.; Fuhr, O.; Wernsdorfer, W.; Ruben, M. Exchange-bias quantum tunnelling in a CO2-based Dy4-single molecule magnet. *Chem. Sci.* **2017**, *8*, 1178–1185, doi:10.1039/c6sc03184f.

30. Zhang, K.; Guo, F.-S.; Wang, Y.-Y. Two {Dy2} single-molecule magnets formed via an in situ reaction by capturing CO 2 from atmosphere under ambient conditions. *Dalton Trans.* **2017**, *46*, 1753–1756, doi:10.1039/C6DT04751C.

31. Baisch, U.; Dell'Amico, D.B.; Calderazzo, F.; Labella, L.; Marchetti, F.; Merigo, A. N,N-dialkylcarbamato lanthanide complexes, a series of isotypical coordination compounds. *Eur. J. Inorg. Chem.* **2004**, 1219–1224, doi:10.1002/ejic.200300649.

32. Zhang, C.; Liu, R.; Zhang, J.; Chen, Z.; Zhou, X. Reactivity of lanthanocene hydroxides toward ketene, isocyanate, lanthanocene alkyl, and triscyclopentadienyllanthanide

complexes. *Inorg. Chem.* **2006**, *45*, 5867–5877, doi:10.1021/ic0602998.

33. Neis, C.; Weyhermüller, T.; Bill, E.; Stucky, S.; Hegetschweiler, K. Carbamates of Polyamines – Versatile Building Blocks for the Construction of Polynuclear Metal Complexes. *Eur. J. Inorg. Chem.* **2008**, 1019–1021, doi:10.1002/ejic.200701320.

34. Jayarathne, U.; Hazari, N.; Bernskoetter, W.H. Selective Iron-Catalyzed N -Formylation of Amines using Dihydrogen and Carbon Dioxide. *ACS Catal.* **2018**, *8*, 1338–1345, doi:10.1021/acscatal.7b03834.

35. Uhl, W.; Willeke, M.; Hepp, A.; Pleschka, D.; Layh, M. A Dimeric Gallium Hydrazide as an Active Lewis Pair - Complexation and Activation of Me 2 GaH and Various Heterocumulenes. *Z. Anorg. Allg. Chem.* **2017**, *643*, 387–397, doi:10.1002/zaac.201600456.

36. Feier-Iova, O.; Linti, G. Synthesis and Structure of a Carbamato-bridgded Digallyl-ferrocenophane – Fixation of Carbon Dioxide with Aminogallanes. *Z. Anorg. Allg. Chem.* **2008**, *634*, 559–564, doi:10.1002/zaac.200700464.

37. Bortoluzzi, M.; Bresciani, G.; Marchetti, F.; Pampaloni, G.; Zacchini, S. Synthesis and structural characterization of mixed halide–N,N-diethylcarbamates of group 4 metals, including a case of unusual tetrahydrofuran activation. *New J. Chem.* **2017**, *41*, 1781–1789, doi:10.1039/C6NJ03489F.

38. Truscott, B.J.; Nelson, D.J.; Slawin, A.M.Z.; Nolan, S.P. CO2 fixation employing an iridium(I)-hydroxide complex. *Chem. Commun.* **2014**, *50*, 286–288, doi:10.1039/C3CC46922K.

39. Dobereiner, G.E.; Wu, J.; Manas, M.G.; Schley, N.D.; Takase, M.K.; Crabtree, R.H.; Hazari, N.; Maseras, F.; Nova, A. Mild, Reversible Reaction of Iridium(III) Amido Complexes with Carbon Dioxide. *Inorg. Chem.* **2012**, *51*, 9683–9693, doi:10.1021/ic300923c.

40. Cristóbal, C.; Hernández, Y.A.; López-Serrano, J.; Paneque, M.; Petronilho, A.; Poveda, M.L.; Salazar, V.; Vattier, F.; Álvarez, E.; Maya, C.; et al. Reactivity Studies of Iridium Pyridylidenes [Tp Me2 Ir(C 6 H 5 ) 2 (C(CH) 3 C(R)NH] (R=H, Me, Ph). *Chem. -Eur. J.* **2013**, *19*, 4003–4020, doi:10.1002/chem.201203818.

41. Kinauer, M.; Diefenbach, M.; Bamberger, H.; Demeshko, S.; Reijerse, E.J.; Volkmann, C.; Würtele, C.; Van Slageren, J.; De Bruin, B.; Holthausen, M.C.; et al. An iridium(iii/iv/v) redox series featuring a terminal imido complex with triplet ground state. *Chem. Sci.* **2018**, *9*, 4325–4332, doi:10.1039/c8sc01113c.

42. Belli Dell'Amico, D.; Biagini, P.; Chiaberge, S.; Falchi, L.; Labella, L.; Lezzerini, M.; Marchetti, F.; Samaritani, S. Partial and exhaustive hydrolysis of lanthanide N,N-dialkylcarbamato complexes. A viable access to lanthanide mixed oxides. *Polyhedron* **2015**, *102*, 452–461, doi:10.1016/j.poly.2015.10.009.

43. Gauld, R.M.; Kennedy, A.R.; McLellan, R.; Barker, J.; Reid, J.; Mulvey, R.E. Diverse outcomes

of CO 2 fixation using alkali metal amides including formation of a heterobimetallic lithium-sodium carbamato-anhydride via lithium-sodium bis-hexamethyldisilazide. *Chem. Commun.* **2019**, *55*, 1478–1481, doi:10.1039/c8cc08308h.

44. Caudle, M.T.; Brennessel, W.W.; Young, V.G. Structural variability and dynamics in carboxylato- and carbamatomagnesium bromides. Relationship to the carboxylate shift. *Inorg. Chem.* **2005**, *44*, 3233–3240, doi:10.1021/ic048442p.

45. Dell′ Amico, D.B.; Calderazzo, F.; Labella, L.; Marchetti, F.; Martini, M.; Mazzoncini, I. N,N-Dimethylcarbamato derivatives of magnesium starting from the metal oxide. *C. R. Chim.* **2004**, *7*, 877–884, doi:10.1016/j.crci.2004.04.006.

46. Belli Dell′Amico, D.; Labella, L.; Marchetti, F.; Mastrorilli, P.; Samaritani, S.; Todisco, S. Oxidation by dioxygen of manganese(II) and iron(II) complexes. *Polyhedron* **2013**, *65*, 275–281, doi:10.1016/j.poly.2013.08.011.

47. Bortoluzzi, M.; Ghini, F.; Hayatifar, M.; Marchetti, F.; Pampaloni, G.; Zacchini, S. Oxido- and sulfidoniobium(V) N,N-diethylcarbamates: Synthesis, characterization and DFT study. *Eur. J. Inorg. Chem.* **2013**, 3112–3118, doi:10.1002/ejic.201300219.

48. Xu, X.P.; Qi, R.P.; Xu, B.; Yao, Y.M.; Nie, K.; Zhang, Y.; Shen, Q. Synthesis, reactivity and structural characterization of lanthanide hydroxides stabilized by a carbon-bridged bis(phenolate) ligand. *Polyhedron* **2009**, *28*, 574–578, doi:10.1016/j.poly.2008.11.031.

49. Lozan, V.; Holldorf, J.; Kersting, B. Preparation and characterization of macrocyclic dinickel complexes coligated by monoalkyl- and dialkylcarbamates. *Inorg. Chim. Acta* **2009**, *362*, 793–798, doi:10.1016/j.ica.2008.03.016.

50. Schmeier, T.J.; Nova, A.; Hazari, N.; Maseras, F. Synthesis of PCP-Supported Nickel Complexes and their Reactivity with Carbon Dioxide. *Chem. -Eur. J.* **2012**, *18*, 6915–6927, doi:10.1002/chem.201103992.

51. Hao, J.; Vabre, B.; Mougang-Soumé, B.; Zargarian, D. Small Molecule Activation by POC sp 3OP-Nickel Complexes. *Chem. -Eur. J.* **2014**, *20*, 12544–12552, doi:10.1002/chem.201402933.

52. Mindiola, D.J.; Waterman, R.; Iluc, V.M.; Cundari, T.R.; Hillhouse, G.L. Carbon–Hydrogen Bond Activation, C–N Bond Coupling, and Cycloaddition Reactivity of a Three-Coordinate Nickel Complex Featuring a Terminal Imido Ligand. *Inorg. Chem.* **2014**, *53*, 13227–13238, doi:10.1021/ic5026153.

53. Mochizuki, K.; Kondou, H.; Ando, K.; Kawasumi, T.; Takahashi, J. Degradation of urea mediated by dinickel(II) complexes with the binucleating ligand N,N′-bis[2-(N,N-dimethyl)aminoethyl]-N,N′-dimethyl-1,3-diamino-2-hydroxypropane (HL). *Inorg. Chim. Acta* **2016**, *441*, 50–56, doi:10.1016/j.ica.2015.10.047.

54. Kim, J.; Park, K.; Lee, Y. Synthesis and characterization of a four-coordinate nickel carbamato

species (MeSiP i Pr 2 )Ni(OC(O)NHMes) generated from the reaction of (MeSiP i Pr 2 )Ni(NHMes) with CO 2. *Inorg. Chim. Acta* **2017**, *460*, 55–62, doi:10.1016/j.ica.2016.08.042.

55. Yao, C.; Chakraborty, P.; Aresu, E.; Li, H.; Guan, C.; Zhou, C.; Liang, L.-C.; Huang, K.-W. Monomeric nickel hydroxide stabilized by a sterically demanding phosphorus–nitrogen PN 3 P-pincer ligand: synthesis, reactivity and catalysis. *Dalton Trans.* **2018**, *47*, 16057–16065, doi:10.1039/C8DT03403F.

56. Kim, J.; Kim, Y.-E.; Park, K.; Lee, Y. A Silyl-Nickel Moiety as a Metal–Ligand Cooperative Site. *Inorg. Chem.* **2019**, *58*, 11534–11545, doi:10.1021/acs.inorgchem.9b01388.

57. Goodner, S.J.; Grünwald, A.; Heinemann, F.W.; Munz, D. Carbon Dioxide Activation by a Palladium Terminal Imido Complex. *Aust. J. Chem.* **2019**, *72*, 900, doi:10.1071/CH19323.

58. Comanescu, C.C.; Iluc, V.M. E-H (E = N, O) bond activation by a nucleophilic palladium carbene. *Polyhedron* **2018**, *143*, 176–183, doi:10.1016/j.poly.2017.10.007.

59. Wilson, J.J.; Lippard, S.J. Synthesis, Characterization, and Cytotoxicity of Platinum(IV) Carbamate Complexes. *Inorg. Chem.* **2011**, *50*, 3103–3115, doi:10.1021/ic2000816.

60. Pichler, V.; Mayr, J.; Heffeter, P.; Dömötör, O.; Enyedy, É.A.; Hermann, G.; Groza, D.; Köllensperger, G.; Galanksi, M.; Berger, W.; et al. Maleimide-functionalised platinum(iv) complexes as a synthetic platform for targeted drug delivery. *Chem. Commun.* **2013**, *49*, 2249, doi:10.1039/c3cc39258a.

61. Mayr, J.; Heffeter, P.; Groza, D.; Galvez, L.; Koellensperger, G.; Roller, A.; Alte, B.; Haider, M.; Berger, W.; Kowol, C.R.; et al. An albumin-based tumor-targeted oxaliplatin prodrug with distinctly improved anticancer activity in vivo. *Chem. Sci.* **2017**, *8*, 2241–2250, doi:10.1039/C6SC03862J.

62. Cuesta, L.; Gerbino, D.C.; Hevia, E.; Morales, D.; Navarro Clemente, M.E.; Pérez, J.; Riera, L.; Riera, V.; Miguel, D.; Del Río, I.; et al. Reactivity of Molybdenum and Rhenium Hydroxo-Carbonyl Complexes toward Organic Electrophiles. *Chem. -Eur. J.* **2004**, *10*, 1765–1777, doi:10.1002/chem.200305577.

63. Zhu, Y.; Smith, D.A.; Herbert, D.E.; Gatard, S.; Ozerov, O. V. C–H and C–O oxidative addition in reactions of aryl carboxylates with a PNP pincer-ligated Rh(I) fragment. *Chem. Commun.* **2012**, *48*, 218–220, doi:10.1039/C1CC15845G.

64. Dell'Amico, D.B.; Calderazzo, F.; Englert, U.; Labella, L.; Marchetti, F.; Specos, M. New N,N-diisopropylcarbamato complexes of ruthenium(II) as catalytic precursors for olefin hydrogenation. *Eur. J. Inorg. Chem.* **2004**, 3938–3945, doi:10.1002/ejic.200400014.

65. Norris, M.R.; Flowers, S.E.; Mathews, A.M.; Cossairt, B.M. H2 Production Mediated by CO2 via Initial Reduction to Formate. *Organometallics* **2016**, *35*, 2778–2781, doi:10.1021/acs.organomet.6b00595.

66. Mathis, C.L.; Geary, J.; Ardon, Y.; Reese, M.S.; Philliber, M.A.; VanderLinden, R.T.; Saouma, C.T. Thermodynamic Analysis of Metal–Ligand Cooperativity of PNP Ru Complexes: Implications for CO 2 Hydrogenation to Methanol and Catalyst Inhibition. *J. Am. Chem. Soc.* **2019**, *141*, 14317–14328, doi:10.1021/jacs.9b06760.

67. Chu, J.; Lu, E.; Liu, Z.; Chen, Y.; Leng, X.; Song, H. Reactivity of a Scandium Terminal Imido Complex Towards Unsaturated Substrates. *Angew. Chem. Int. Ed.* **2011**, *50*, 7677–7680, doi:10.1002/anie.201102267.

68. Harris, L.A.M.; Coles, M.P.; Fulton, J.R. Synthesis and reactivity of tin amide complexes. *Inorg. Chim. Acta* **2011**, *369*, 97–102, doi:10.1016/j.ica.2010.12.009.

69. Stewart, C.A.; Dickie, D.A.; Tang, Y.; Kemp, R.A. Insertion reactions of CO2, OCS, and CS2into the Sn-N bonds of (Me2N)2Sn: NMR and X-ray structural characterization of the products. *Inorg. Chim. Acta* **2011**, *376*, 73–79, doi:10.1016/j.ica.2011.05.036.

70. Armelao, L.; Belli Dellamico, D.; Biagini, P.; Bottaro, G.; Chiaberge, S.; Falvo, P.; Labella, L.; Marchetti, F.; Samaritani, S. Preparation of N,N -dialkylcarbamato lanthanide complexes by extraction of lanthanide ions from aqueous solution into hydrocarbons. *Inorg. Chem.* **2014**, *53*, 4861–4871, doi:10.1021/ic402936z.

71. Mendiratta, A.; Cummins, C.C.; Cotton, F.A.; Ibragimov, S.A.; Murillo, C.A.; Villagrán, D. A Diamagnetic Dititanium(III) Paddlewheel Complex with No Direct Metal−Metal Bond. *Inorg. Chem.* **2006**, *45*, 4328–4330, doi:10.1021/ic0602650.

72. Forte, C.; Hayatifar, M.; Pampaloni, G.; Galletti, A.M.R.; Renili, F.; Zacchini, S. Ethylene polymerization using novel titanium catalytic precursors bearing N,N-dialkylcarbamato ligands. *J. Polym. Sci. Part A Polym. Chem.* **2011**, *49*, 3338–3345, doi:10.1002/pola.24770.

73. Hayatifar, M.; Forte, C.; Pampaloni, G.; Kissin, Y. V.; Maria Raspolli Galletti, A.; Zacchini, S. Titanium complexes bearing carbamato ligands as catalytic precursors for propylene polymerization reactions. *J. Polym. Sci. Part A Polym. Chem.* **2013**, *51*, 4095–4102, doi:10.1002/pola.26816.

74. Boyd, C.L.; Clot, E.; Guiducci, A.E.; Mountford, P. Pendant Arm Functionalized Benzamidinate Titanium Imido Compounds: Experimental and Computational Studies of Their Reactions with CO2. *Organometallics* **2005**, *24*, 2347–2367, doi:10.1021/om049026f.

75. Guiducci, A.E.; Boyd, C.L.; Clot, E.; Mountford, P. Reactions of cyclopentadienyl-amidinate titanium imido compounds with CO2: cycloaddition-extrusion vs. cycloaddition-insertion. *Dalton Trans.* **2009**, 5960, doi:10.1039/b901774g.

76. Higgins Frey, J.A.; Cloke, F.G.N.; Roe, S.M. Synthesis and Reactivity of a Mixed-Sandwich Uranium(IV) Primary Amido Complex. *Organometallics* **2015**, *34*, 2102–2105, doi:10.1021/om501190x.

77. Bart, S.C.; Anthon, C.; Heinemann, F.W.; Bill, E.; Edelstein, N.M.; Meyer, K. Carbon Dioxide Activation with Sterically Pressured Mid- and High-Valent Uranium Complexes. *J. Am. Chem. Soc.* **2008**, *130*, 12536–12546, doi:10.1021/ja804263w.

78. Schmidt, A.-C.; Heinemann, F.W.; Maron, L.; Meyer, K. A Series of Uranium (IV, V, VI) Tritylimido Complexes, Their Molecular and Electronic Structures and Reactivity with CO 2. *Inorg. Chem.* **2014**, *53*, 13142–13153, doi:10.1021/ic5023517.

79. Falcone, M.; Chatelain, L.; Mazzanti, M. Nucleophilic Reactivity of a Nitride-Bridged Diuranium(IV) Complex: CO 2 and CS 2 Functionalization. *Angew. Chem. Int. Ed.* **2016**, *55*, 4074–4078, doi:10.1002/anie.201600158.

80. Webster, C.L.; Langeslay, R.R.; Ziller, J.W.; Evans, W.J. Synthetic Utility of Tetrabutylammonium Salts in Uranium Metallocene Chemistry. *Organometallics* **2016**, *35*, 520–527, doi:10.1021/acs.organomet.5b00942.

81. Falcone, M.; Poon, L.N.; Fadaei Tirani, F.; Mazzanti, M. Reversible Dihydrogen Activation and Hydride Transfer by a Uranium Nitride Complex. *Angew. Chem. Int. Ed.* **2018**, *57*, 3697–3700, doi:10.1002/anie.201800203.

82. Masci, B.; Thuéry, P. A Tetrahomodioxacalix[6]arene as a Ditopic Ligand for Uranyl Ions with Carbonate or Carbamate Bridges. *Supramol. Chem.* **2003**, *15*, 101–108, doi:10.1080/1061027021000023113.

83. Chakraborty, S.; Blacque, O.; Berke, H. Ligand assisted carbon dioxide activation and hydrogenation using molybdenum and tungsten amides. *Dalton Trans.* **2015**, *44*, 6560–6570, doi:10.1039/C5DT00278H.

84. Dell'Amico, D.B.; Calderazzo, F.; Labella, L.; Marchetti, F. A facile synthesis of Zn4(μ4-O)(O 2CNMe2)6. *Inorg. Chim. Acta* **2003**, *350*, 661–664, doi:10.1016/S0020-1693(03)00107-5.

85. McCowan, C.S.; Buss, C.E.; Young, V.G.; McDonnell, R.L.; Caudle, M.T. Chloro(diethylamino)tris(μ-diethylcarbamato)dizinc(II): An example of the generality of the threefold paddlewheel structure in carbamatozinc chemistry. *Acta Crystallogr. Sect. E Struct. Rep. Online* **2004**, *60*, 285–287, doi:10.1107/S1600536804002193.

86. Tang, Y.; Kassel, W.S.; Zakharov, L.N.; Rheingold, A.L.; Kemp, R.A. Insertion reactions of carbon dioxide into Zn-N bonds: Syntheses and structures of tetrameric and dimeric alkylzinc carbamato complexes. *Inorg. Chem.* **2005**, *44*, 359–364, doi:10.1021/ic048830r.

87. Malik, M.A.; O'Brien, P.; Motevalli, M.; Abrahams, I. The adoption of the beryllium acetate structural motif in zinc oxycarbamates, oxythiocarbamates and oxythiophosphinates. *Polyhedron* **2006**, *25*, 241–250, doi:10.1016/j.poly.2005.06.033.

88. Yamaguchi, S.; Takahashi, T.; Wada, A.; Funahashi, Y.; Ozawa, T.; Jitsukawa, K.; Masuda, H. Fixation of CO 2 by Hydroxozinc(II) Complex with Pyridylamino Type Ligand. *Chem. Lett.*

**2007**, *36*, 842–843, doi:10.1246/cl.2007.842.

89. Neuhäuser, C.; Domide, D.; Mautz, J.; Kaifer, E.; Himmel, H.J. Electron density controlled carbamate ligand binding mode: Towards a better understanding of metalloenzyme activity. *Dalton Trans.* **2008**, *4*, 1821–1824, doi:10.1039/b800687n.

90. Domide, D.; Kaifer, E.; Mautz, J.; Walter, O.; Behrens, S.; Himmel, H. Synthesis and Characterisation of Some New Zinc Carbamate Complexes Formed by CO 2 Fixation and Their Use as Precursors for ZnO Particles under Mild Conditions. *Eur. J. Inorg. Chem.* **2008**, 3177–3185, doi:10.1002/ejic.200701308.

91. Haywood, P.F.; Hill, M.R.; Roberts, N.K.; Craig, D.C.; Russell, J.J.; Lamb, R.N. Synthesis and Isomerisation Reactions of Tetranuclear and Octanuclear (Carbamato)zinc Complexes. *Eur. J. Inorg. Chem.* **2008**, *2008*, 2024–2032, doi:10.1002/ejic.200700736.

92. Notni, J.; Schenk, S.; Görls, H.; Breitzke, H.; Anders, E. Formation of a Unique Zinc Carbamate by CO 2 Fixation: Implications for the Reactivity of Tetra-Azamacrocycle Ligated Zn(II) Complexes. *Inorg. Chem.* **2008**, *47*, 1382–1390, doi:10.1021/ic701899u.

93. Domide, D.; Neuhäuser, C.; Kaifer, E.; Wadepohl, H.; Himmel, H.J. Synthesis of trinuclear, dinuclear and mononuclear carbamato-zinc complexes from tetranuclear precursors: A top-down synthetic approach to new carbamates. *Eur. J. Inorg. Chem.* **2009**, 2170–0178, doi:10.1002/ejic.200801136.

94. Kahnes, M.; Görls, H.; Westerhausen, M. Synthesis of Dimeric Methylzinc N,N-Bis(2-pyridylmethyl)carbamate via Addition of CO2 to a Methylzinc Amide. *Z. Anorg. Allg. Chem.* **2011**, *637*, 397–400, doi:10.1002/zaac.201000397.

95. Rodriguez, M.A.; Sava, D.F.; Nenoff, T.M. catena -Poly[zinc-tris(μ-dimethylcarbamato-κ 2 O : O ′)-zinc-μ-(2-phenylbenzimidazolido-κ 2 N : N ′]. *Acta Crystallogr. Sect. E Struct. Rep. Online* **2012**, *68*, m59–m60, doi:10.1107/S1600536811053177.

96. Yin, C.-L.; Hu, Z.-B.; Long, Q.-Q.; Wang, H.-S.; Li, J.; Song, Y.; Zhang, Z.-C.; Zhang, Y.-Q.; Pan, Z.-Q. Single molecule magnet behaviors of Zn 4 Ln 2 (Ln = Dy III , Tb III ) complexes with multidentate organic ligands formed by absorption of CO 2 in air through in situ reactions. *Dalton Trans.* **2019**, *48*, 512–522, doi:10.1039/C8DT03849J.

97. Normand, A.T.; Daniliuc, C.G.; Wibbeling, B.; Kehr, G.; Le Gendre, P.; Erker, G. Phosphido- and Amidozirconocene Cation-Based Frustrated Lewis Pair Chemistry. *J. Am. Chem. Soc.* **2015**, *137*, 10796–10808, doi:10.1021/jacs.5b06551.

98. Johnson, M.W.; Shevick, S.L.; Toste, F.D.; Bergman, R.G. Preparation and reactivity of terminal gold( <scp>i</scp> ) amides and phosphides. *Chem. Sci.* **2013**, *4*, 1023–1027, doi:10.1039/C2SC21519E.

99. Jackson, W.G.; McKeon, J.A.; Balahura, R.J. N

-Methylmonothiocarbamatopentamminecobalt(III): Restricted C−N Bond Rotation and the Acid-Catalyzed O- to S-Bonded Rearrangement. *Inorg. Chem.* **2004**, *43*, 4889–4896, doi:10.1021/ic040047b.

100. Roth, C.E.; Dibenedetto, A.; Aresta, M. Synthesis and Characterization of Chloro- and Alkyliron Complexes with N-Donor Ligands and Their Reactivity towards CO 2. *Eur. J. Inorg. Chem.* **2015**, 5066–5073, doi:10.1002/ejic.201500657.

101. Cámpora, J.; Matas, I.; Palma, P.; Álvarez, E.; Graiff, C.; Tiripicchio, A. Monomeric Alkoxo and Amido Methylnickel(II) Complexes. Synthesis and Heterocumulene Insertion Chemistry. *Organometallics* **2007**, *26*, 3840–3849, doi:10.1021/om7002909.

102. Mousa, A.H.; Bendix, J.; Wendt, O.F. Synthesis, Characterization, and Reactivity of PCN Pincer Nickel Complexes. *Organometallics* **2018**, *37*, 2581–2593, doi:10.1021/acs.organomet.8b00333.

103. Martínez-Prieto, L.M.; Palma, P.; Cámpora, J. Monomeric alkoxide and alkylcarbonate complexes of nickel and palladium stabilized with the iPr PCP pincer ligand: a model for the catalytic carboxylation of alcohols to alkyl carbonates. *Dalton Trans.* **2019**, *48*, 1351–1366, doi:10.1039/C8DT04919J.

104. Seul, J.-M.; Park, S. Palladium(II) p-Tolylamide and Reaction with CO 2 to Generate a Carbamato Derivative. *Bull. Korean Chem. Soc.* **2010**, *31*, 3745–3748, doi:10.5012/bkcs.2010.31.12.3745.

105. Baisch, U.; Schnick, W. Synthese und Kristallstruktur vonbis-1, 3-Dimethoxyethan-trichloro-samarium(III) undtris-N, N-Diisopropylcarbamato-samarium(III). *Z. Anorg. Allg. Chem.* **2003**, *629*, 2073–2078, doi:10.1002/zaac.200300143.
